# Exact p-value calculation for heterotypic clusters of regulatory motifs and its application in computational annotation of *cis*-regulatory modules

**DOI:** 10.1186/1748-7188-2-13

**Published:** 2007-10-10

**Authors:** Valentina Boeva, Julien Clément, Mireille Régnier, Mikhail A Roytberg, Vsevolod J Makeev

**Affiliations:** 1 Institute of Genetics and Selection of Industrial Microorganisms, GosNIIGenetika, 117545 Moscow, Russia; 2 MIGEC, INRIA Rocquencourt, 78153 Le Chesnay, France; 3 GREYC, CNRS UMR 6072, Laboratoire d'informatique, 14032 Caen, France; 4 Institute of Mathematical Problems of Biology, Russian Academy of Sciences, Puschino, Moscow Region, Russia; 5 Puschino State University, Puschino, Moscow Region, Russia; 6 Engelhardt Institute of Molecular Biology, Russian Academy of Sciences, Moscow, Russia

## Abstract

**Background:**

*cis*-Regulatory modules (CRMs) of eukaryotic genes often contain multiple binding sites for transcription factors. The phenomenon that binding sites form clusters in CRMs is exploited in many algorithms to locate CRMs in a genome. This gives rise to the problem of calculating the statistical significance of the event that multiple sites, recognized by different factors, would be found simultaneously in a text of a fixed length. The main difficulty comes from overlapping occurrences of motifs. So far, no tools have been developed allowing the computation of *p*-values for simultaneous occurrences of different motifs which can overlap.

**Results:**

We developed and implemented an algorithm computing the *p*-value that *s *different motifs occur respectively *k*_1_, ..., *k*_*s *_or more times, possibly overlapping, in a random text. Motifs can be represented with a majority of popular motif models, but in all cases, without indels. Zero or first order Markov chains can be adopted as a model for the random text. The computational tool was tested on the set of *cis*-regulatory modules involved in *D. melanogaster *early development, for which there exists an annotation of binding sites for transcription factors. Our test allowed us to correctly identify transcription factors cooperatively/competitively binding to DNA.

**Method:**

The algorithm that precisely computes the probability of simultaneous motif occurrences is inspired by the Aho-Corasick automaton and employs a prefix tree together with a transition function. The algorithm runs with the *O*(*n*|Σ|(*m*|ℋ
 MathType@MTEF@5@5@+=feaafiart1ev1aaatCvAUfKttLearuWrP9MDH5MBPbIqV92AaeXatLxBI9gBaebbnrfifHhDYfgasaacH8akY=wiFfYdH8Gipec8Eeeu0xXdbba9frFj0=OqFfea0dXdd9vqai=hGuQ8kuc9pgc9s8qqaq=dirpe0xb9q8qiLsFr0=vr0=vr0dc8meaabaqaciaacaGaaeqabaqabeGadaaakeaat0uy0HwzTfgDPnwy1egaryqtHrhAL1wy0L2yHvdaiqaacqWFlecsaaa@3762@| + *K*|*σ*|^*K*^) ∏_*i *_*k*_*i*_) time complexity, where *n *is the length of the text, |Σ| is the alphabet size, *m *is the maximal motif length, |ℋ
 MathType@MTEF@5@5@+=feaafiart1ev1aaatCvAUfKttLearuWrP9MDH5MBPbIqV92AaeXatLxBI9gBaebbnrfifHhDYfgasaacH8akY=wiFfYdH8Gipec8Eeeu0xXdbba9frFj0=OqFfea0dXdd9vqai=hGuQ8kuc9pgc9s8qqaq=dirpe0xb9q8qiLsFr0=vr0=vr0dc8meaabaqaciaacaGaaeqabaqabeGadaaakeaat0uy0HwzTfgDPnwy1egaryqtHrhAL1wy0L2yHvdaiqaacqWFlecsaaa@3762@| is the total number of words in motifs, *K *is the order of Markov model, and *k*_*i *_is the number of occurrences of the *i*th motif.

**Conclusion:**

The primary objective of the program is to assess the likelihood that a given DNA segment is CRM regulated with a known set of regulatory factors. In addition, the program can also be used to select the appropriate threshold for PWM scanning. Another application is assessing similarity of different motifs.

**Availability:**

Project web page, stand-alone version and documentation can be found at

## Background

During the past few years, a number of computational tools have been designed [1-3] for locating potential *transcription factor binding sites *(TFBSs) in nucleotide sequences, e.g., in compilations of sequences upstream of putative co-regulated genes. In parallel, experimental approaches were developed [4], which allowed identification of binding motifs for many different transcription factors. Experimental [5] and bioinformatical [6] studies demonstrated that sequences of regulatory DNA that bind transcription factors can exhibit many different types of architecture. In eukaryotes TFBSs found in DNA sequences often form rather dense clusters: this was demonstrated both by experimental [5,7] and computational [8,9] methods. Such clusters can contain sites binding the same factor or several different factors [10]. The *cis*-regulatory module (CRM) in this case contains respectively homotypic or heterotypic clusters of motifs specifically recognized by binding proteins [11].

The particular arrangement of motifs in a homotypic or heterotypic cluster is not random, and it is commonly accepted, that the motif arrangement within a CRM is important for its functionality [12-20]. Bioinformatics studies indicate that antagonistic factors often bind to overlapping sites [21] whereas synergetic factors are often positioned within a fixed distance [20], often close to the multiple of 10.2 bp, the DNA double-helix pitch value [21].

Non-random arrangements of TFBSs within regulatory segments of DNA sequences are exploited in several TFBS identification tools, and it was observed that cooperativity-based discrimination of TFBSs surpasses the performance of models for individual TFBSs [22].

On observing a cluster of TFBSs in some genome segment one can calculate the probability of observing similar site arrangements in a random sequence. This idea of evaluating the statistical significance of heterotypic clusters of sites was implemented in many programs including ClusterDraw [23], ModuleSearcher [24], MCAST [25], eCIS-ANALYST [26], Cister [27], Cluster-Buster [28] and TargetExplorer [29]. At the moment, such programs use empirical procedures like motif counting in biological and simulated sequences to assess the significance of observed site clustering. But it is highly desirable to have a good statistical measure of site clustering, and we believe that the best measure is the *p*-value of obtaining the observed cluster by chance in a random sequence of a Markov or Bernoulli (common name for Markov chain of order 0) type. In the case of heterotypic clusters one needs to take into account possible overlapping occurrences of different motifs, a problem that was considered difficult until now [30]. In the case of homotypic clusters, an approximate statistical scoring function was constructed [8,31]; this approach has been implemented in algorithms like FLYENHANCER [32], SCORE [33], and CLUSTER [34]. However, this approximation performs poorly for highly overlapping TFBSs. One cannot ignore site overlapping if the motifs are fuzzy (highly degenerate), which is often the case for so-called "shadow sites" [31]. In the case of heterotypic clusters, competing factors can bind even to very well determined motifs that overlap.

### Representation of protein binding motifs in nucleotide sequences

Experimental methods on protein binding to DNA usually locate some DNA segment, or word in DNA text, as a probable binding target. Proteins can bind to similar DNA words [4], the whole assembly of which can be called a motif. The simplest motif representation is the enumeration of sequences that can be bound by a transcription factor (TF) [35]. Sometimes, information about binding sites can be found in SELEX [36,37] or Protein Binding Microarray (PBM) experiments [38]. However, it is possible that such experiments do not give the exhaustive list of sequences of binding sites, so one needs to expand the list of putative binding sites using an appropriate criterion, which brings about the problem of the generalization of several known examples.

For instance, several words aligned with mismatches, can be generalized to IUPAC string (like RSTGACTNMNW for AP-1 binding sites [39]) by disregarding correlated substitutions in different motif positions [40]. Another example of generalization is the set of words that can deviate from a consensus word for less than a given number of mismatches.

The most popular way to represent binding sites is a Position Weight Matrix (PWM), which is also called position-specific weight matrix (PSWM) or position-specific scoring matrix (PSSM) [41]. For a text with length *D *over an alphabet Σ with |Σ| symbols, a PWM is a |Σ| × *D *matrix: each row corresponding to a symbol of the alphabet Σ, and each column to a position in the motif. For DNA texts, one has Σ = {*A*, *C*, *G*, *T*}. The PWM score is defined as ∑i=1Lmω(i),i
 MathType@MTEF@5@5@+=feaafiart1ev1aaatCvAUfKttLearuWrP9MDH5MBPbIqV92AaeXatLxBI9gBaebbnrfifHhDYfgasaacH8akY=wiFfYdH8Gipec8Eeeu0xXdbba9frFj0=OqFfea0dXdd9vqai=hGuQ8kuc9pgc9s8qqaq=dirpe0xb9q8qiLsFr0=vr0=vr0dc8meaabaqaciaacaGaaeqabaqabeGadaaakeaadaaeWaqaaiabd2gaTnaaBaaaleaaiiGacqWFjpWDcqGGOaakcqWGPbqAcqGGPaqkcqGGSaalcqWGPbqAaeqaaaqaaiabdMgaPjabg2da9iabigdaXaqaaiabdYeambqdcqGHris5aaaa@3BC0@, where *i *represents a position in the *D*-substring, *ω*(*i*) the symbol at position *i *in the substring, and *m*_*α, i *_the score in row *α*, column *i *of the matrix. So, given a cutoff value, one gets a list of *D*-sequences that score higher than this cutoff; thus representing possible DNA binding sites for the protein.

Any of the three motif representations above can be converted to a list of words. The same is true for many other representations of motifs. In this study, we consider only the motifs that can be represented as a set of words.

### P-value for clusters of motif occurrences, problem formulation

The objective of this work is to develop a statistical criterion to assess clustering of TFBS. Intuitively, a TFBS cluster is a DNA segment simultaneously containing "too many" TFBSs for given factor proteins; such a segment can often operate as a CRM regulated by these TFs. From a formal point of view, the problem we address here is as follows. Let *s *sets of words ℋ1,...,ℋs
 MathType@MTEF@5@5@+=feaafiart1ev1aaatCvAUfKttLearuWrP9MDH5MBPbIqV92AaeXatLxBI9gBaebbnrfifHhDYfgasaacH8akY=wiFfYdH8Gipec8Eeeu0xXdbba9frFj0=OqFfea0dXdd9vqai=hGuQ8kuc9pgc9s8qqaq=dirpe0xb9q8qiLsFr0=vr0=vr0dc8meaabaqaciaacaGaaeqabaqabeGadaaakeaat0uy0HwzTfgDPnwy1egaryqtHrhAL1wy0L2yHvdaiqaacqWFlecsdaWgaaWcbaGaeGymaedabeaakiabcYcaSiabc6caUiabc6caUiabc6caUiabcYcaSiab=TqiinaaBaaaleaacqWGZbWCaeqaaaaa@3F88@ be given. Typically, each set ℋ
 MathType@MTEF@5@5@+=feaafiart1ev1aaatCvAUfKttLearuWrP9MDH5MBPbIqV92AaeXatLxBI9gBaebbnrfifHhDYfgasaacH8akY=wiFfYdH8Gipec8Eeeu0xXdbba9frFj0=OqFfea0dXdd9vqai=hGuQ8kuc9pgc9s8qqaq=dirpe0xb9q8qiLsFr0=vr0=vr0dc8meaabaqaciaacaGaaeqabaqabeGadaaakeaat0uy0HwzTfgDPnwy1egaryqtHrhAL1wy0L2yHvdaiqaacqWFlecsaaa@3762@_*i *_is associated to a TF motif. Given a *s*-tuple of integers (*k*_1_, ..., *k*_*s*_), we compute the corresponding *p*-value, that is the probability to find at least *k*_*i *_occurrences of words from each set ℋ
 MathType@MTEF@5@5@+=feaafiart1ev1aaatCvAUfKttLearuWrP9MDH5MBPbIqV92AaeXatLxBI9gBaebbnrfifHhDYfgasaacH8akY=wiFfYdH8Gipec8Eeeu0xXdbba9frFj0=OqFfea0dXdd9vqai=hGuQ8kuc9pgc9s8qqaq=dirpe0xb9q8qiLsFr0=vr0=vr0dc8meaabaqaciaacaGaaeqabaqabeGadaaakeaat0uy0HwzTfgDPnwy1egaryqtHrhAL1wy0L2yHvdaiqaacqWFlecsaaa@3762@_*i *_in a random text of size *n*. We assume that the texts where motifs are searched are randomly generated by a Bernoulli process or a Markov model of order *K*. If (*k*_1_, ..., *k*_*s*_) occurrences of motifs ℋ1,...,ℋs
 MathType@MTEF@5@5@+=feaafiart1ev1aaatCvAUfKttLearuWrP9MDH5MBPbIqV92AaeXatLxBI9gBaebbnrfifHhDYfgasaacH8akY=wiFfYdH8Gipec8Eeeu0xXdbba9frFj0=OqFfea0dXdd9vqai=hGuQ8kuc9pgc9s8qqaq=dirpe0xb9q8qiLsFr0=vr0=vr0dc8meaabaqaciaacaGaaeqabaqabeGadaaakeaat0uy0HwzTfgDPnwy1egaryqtHrhAL1wy0L2yHvdaiqaacqWFlecsdaWgaaWcbaGaeGymaedabeaakiabcYcaSiabc6caUiabc6caUiabc6caUiabcYcaSiab=TqiinaaBaaaleaacqWGZbWCaeqaaaaa@3F88@ are found in a DNA segment, the *p*-value can be used to infer if such numbers of occurrences could be found by chance.

## Related work

Most previous works address counting problems for one set of several words ℋ
 MathType@MTEF@5@5@+=feaafiart1ev1aaatCvAUfKttLearuWrP9MDH5MBPbIqV92AaeXatLxBI9gBaebbnrfifHhDYfgasaacH8akY=wiFfYdH8Gipec8Eeeu0xXdbba9frFj0=OqFfea0dXdd9vqai=hGuQ8kuc9pgc9s8qqaq=dirpe0xb9q8qiLsFr0=vr0=vr0dc8meaabaqaciaacaGaaeqabaqabeGadaaakeaat0uy0HwzTfgDPnwy1egaryqtHrhAL1wy0L2yHvdaiqaacqWFlecsaaa@3762@. In contrast, in this paper we deal with a separate counting for several sets of several words ℋ1,...,ℋs
 MathType@MTEF@5@5@+=feaafiart1ev1aaatCvAUfKttLearuWrP9MDH5MBPbIqV92AaeXatLxBI9gBaebbnrfifHhDYfgasaacH8akY=wiFfYdH8Gipec8Eeeu0xXdbba9frFj0=OqFfea0dXdd9vqai=hGuQ8kuc9pgc9s8qqaq=dirpe0xb9q8qiLsFr0=vr0=vr0dc8meaabaqaciaacaGaaeqabaqabeGadaaakeaat0uy0HwzTfgDPnwy1egaryqtHrhAL1wy0L2yHvdaiqaacqWFlecsdaWgaaWcbaGaeGymaedabeaakiabcYcaSiabc6caUiabc6caUiabc6caUiabcYcaSiab=TqiinaaBaaaleaacqWGZbWCaeqaaaaa@3F88@, each set ℋ
 MathType@MTEF@5@5@+=feaafiart1ev1aaatCvAUfKttLearuWrP9MDH5MBPbIqV92AaeXatLxBI9gBaebbnrfifHhDYfgasaacH8akY=wiFfYdH8Gipec8Eeeu0xXdbba9frFj0=OqFfea0dXdd9vqai=hGuQ8kuc9pgc9s8qqaq=dirpe0xb9q8qiLsFr0=vr0=vr0dc8meaabaqaciaacaGaaeqabaqabeGadaaakeaat0uy0HwzTfgDPnwy1egaryqtHrhAL1wy0L2yHvdaiqaacqWFlecsaaa@3762@_*j *_represents one TFBS motif.

All methods of solving the problem of *p*-value calculations for multiple occurrences of words from a set ℋ
 MathType@MTEF@5@5@+=feaafiart1ev1aaatCvAUfKttLearuWrP9MDH5MBPbIqV92AaeXatLxBI9gBaebbnrfifHhDYfgasaacH8akY=wiFfYdH8Gipec8Eeeu0xXdbba9frFj0=OqFfea0dXdd9vqai=hGuQ8kuc9pgc9s8qqaq=dirpe0xb9q8qiLsFr0=vr0=vr0dc8meaabaqaciaacaGaaeqabaqabeGadaaakeaat0uy0HwzTfgDPnwy1egaryqtHrhAL1wy0L2yHvdaiqaacqWFlecsaaa@3762@ study some basic languages. Let *L*_*n *_(ℋ
 MathType@MTEF@5@5@+=feaafiart1ev1aaatCvAUfKttLearuWrP9MDH5MBPbIqV92AaeXatLxBI9gBaebbnrfifHhDYfgasaacH8akY=wiFfYdH8Gipec8Eeeu0xXdbba9frFj0=OqFfea0dXdd9vqai=hGuQ8kuc9pgc9s8qqaq=dirpe0xb9q8qiLsFr0=vr0=vr0dc8meaabaqaciaacaGaaeqabaqabeGadaaakeaat0uy0HwzTfgDPnwy1egaryqtHrhAL1wy0L2yHvdaiqaacqWFlecsaaa@3762@; *k*) be the set of texts of length *n *containing at least *k *occurrences of ℋ
 MathType@MTEF@5@5@+=feaafiart1ev1aaatCvAUfKttLearuWrP9MDH5MBPbIqV92AaeXatLxBI9gBaebbnrfifHhDYfgasaacH8akY=wiFfYdH8Gipec8Eeeu0xXdbba9frFj0=OqFfea0dXdd9vqai=hGuQ8kuc9pgc9s8qqaq=dirpe0xb9q8qiLsFr0=vr0=vr0dc8meaabaqaciaacaGaaeqabaqabeGadaaakeaat0uy0HwzTfgDPnwy1egaryqtHrhAL1wy0L2yHvdaiqaacqWFlecsaaa@3762@. The desired *p*-value would therefore be the probability **P **(*L*_*n *_(ℋ
 MathType@MTEF@5@5@+=feaafiart1ev1aaatCvAUfKttLearuWrP9MDH5MBPbIqV92AaeXatLxBI9gBaebbnrfifHhDYfgasaacH8akY=wiFfYdH8Gipec8Eeeu0xXdbba9frFj0=OqFfea0dXdd9vqai=hGuQ8kuc9pgc9s8qqaq=dirpe0xb9q8qiLsFr0=vr0=vr0dc8meaabaqaciaacaGaaeqabaqabeGadaaakeaat0uy0HwzTfgDPnwy1egaryqtHrhAL1wy0L2yHvdaiqaacqWFlecsaaa@3762@; *k*)). Let ℛℋk
 MathType@MTEF@5@5@+=feaafiart1ev1aaatCvAUfKttLearuWrP9MDH5MBPbIqV92AaeXatLxBI9gBaebbnrfifHhDYfgasaacH8akY=wiFfYdH8Gipec8Eeeu0xXdbba9frFj0=OqFfea0dXdd9vqai=hGuQ8kuc9pgc9s8qqaq=dirpe0xb9q8qiLsFr0=vr0=vr0dc8meaabaqaciaacaGaaeqabaqabeGadaaakeaat0uy0HwzTfgDPnwy1egaryqtHrhAL1wy0L2yHvdaiqaacqWFBeIudaqhaaWcbaGae83cHGeabaGaem4AaSgaaaaa@3A01@ be the set of texts of all lengths that contain exactly *k *words of ℋ
 MathType@MTEF@5@5@+=feaafiart1ev1aaatCvAUfKttLearuWrP9MDH5MBPbIqV92AaeXatLxBI9gBaebbnrfifHhDYfgasaacH8akY=wiFfYdH8Gipec8Eeeu0xXdbba9frFj0=OqFfea0dXdd9vqai=hGuQ8kuc9pgc9s8qqaq=dirpe0xb9q8qiLsFr0=vr0=vr0dc8meaabaqaciaacaGaaeqabaqabeGadaaakeaat0uy0HwzTfgDPnwy1egaryqtHrhAL1wy0L2yHvdaiqaacqWFlecsaaa@3762@, the last one occurring as a suffix [42]. For any H_*j *_in ℋ
 MathType@MTEF@5@5@+=feaafiart1ev1aaatCvAUfKttLearuWrP9MDH5MBPbIqV92AaeXatLxBI9gBaebbnrfifHhDYfgasaacH8akY=wiFfYdH8Gipec8Eeeu0xXdbba9frFj0=OqFfea0dXdd9vqai=hGuQ8kuc9pgc9s8qqaq=dirpe0xb9q8qiLsFr0=vr0=vr0dc8meaabaqaciaacaGaaeqabaqabeGadaaakeaat0uy0HwzTfgDPnwy1egaryqtHrhAL1wy0L2yHvdaiqaacqWFlecsaaa@3762@, let ℛHjk
 MathType@MTEF@5@5@+=feaafiart1ev1aaatCvAUfKttLearuWrP9MDH5MBPbIqV92AaeXatLxBI9gBaebbnrfifHhDYfgasaacH8akY=wiFfYdH8Gipec8Eeeu0xXdbba9frFj0=OqFfea0dXdd9vqai=hGuQ8kuc9pgc9s8qqaq=dirpe0xb9q8qiLsFr0=vr0=vr0dc8meaabaqaciaacaGaaeqabaqabeGadaaakeaat0uy0HwzTfgDPnwy1egaryqtHrhAL1wy0L2yHvdaiqaacqWFBeIudaqhaaWcbaGaeeisaG0aaSbaaWqaaiabdQgaQbqabaaaleaacqWGRbWAaaaaaa@3BB4@ be the subset of ℛℋk
 MathType@MTEF@5@5@+=feaafiart1ev1aaatCvAUfKttLearuWrP9MDH5MBPbIqV92AaeXatLxBI9gBaebbnrfifHhDYfgasaacH8akY=wiFfYdH8Gipec8Eeeu0xXdbba9frFj0=OqFfea0dXdd9vqai=hGuQ8kuc9pgc9s8qqaq=dirpe0xb9q8qiLsFr0=vr0=vr0dc8meaabaqaciaacaGaaeqabaqabeGadaaakeaat0uy0HwzTfgDPnwy1egaryqtHrhAL1wy0L2yHvdaiqaacqWFBeIudaqhaaWcbaGae83cHGeabaGaem4AaSgaaaaa@3A01@ where H_*j *_is a suffix. One observes that a text contains at least *k *occurrences if and only if it admits a prefix in ℛℋk=∪Hj∈ℋℛHjk
 MathType@MTEF@5@5@+=feaafiart1ev1aaatCvAUfKttLearuWrP9MDH5MBPbIqV92AaeXatLxBI9gBaebbnrfifHhDYfgasaacH8akY=wiFfYdH8Gipec8Eeeu0xXdbba9frFj0=OqFfea0dXdd9vqai=hGuQ8kuc9pgc9s8qqaq=dirpe0xb9q8qiLsFr0=vr0=vr0dc8meaabaqaciaacaGaaeqabaqabeGadaaakeaat0uy0HwzTfgDPnwy1egaryqtHrhAL1wy0L2yHvdaiqaacqWFBeIudaqhaaWcbaGae83cHGeabaGaem4AaSgaaOGaeyypa0JaeSOkIu1aaSbaaSqaaiabbIeainaaBaaameaacqWGQbGAaeqaaSGaeyicI4Sae83cHGeabeaakiab=TrisnaaDaaaleaacqqGibasdaWgaaadbaGaemOAaOgabeaaaSqaaiabdUgaRbaaaaa@46ED@. One defines rjk
 MathType@MTEF@5@5@+=feaafiart1ev1aaatCvAUfKttLearuWrP9MDH5MBPbIqV92AaeXatLxBI9gBaebbnrfifHhDYfgasaacH8akY=wiFfYdH8Gipec8Eeeu0xXdbba9frFj0=OqFfea0dXdd9vqai=hGuQ8kuc9pgc9s8qqaq=dirpe0xb9q8qiLsFr0=vr0=vr0dc8meaabaqaciaacaGaaeqabaqabeGadaaakeaacqWGYbGCdaqhaaWcbaGaemOAaOgabaGaem4AaSgaaaaa@3102@ (*p*) as the probability that a text of size *p *be in set ℛHjk
 MathType@MTEF@5@5@+=feaafiart1ev1aaatCvAUfKttLearuWrP9MDH5MBPbIqV92AaeXatLxBI9gBaebbnrfifHhDYfgasaacH8akY=wiFfYdH8Gipec8Eeeu0xXdbba9frFj0=OqFfea0dXdd9vqai=hGuQ8kuc9pgc9s8qqaq=dirpe0xb9q8qiLsFr0=vr0=vr0dc8meaabaqaciaacaGaaeqabaqabeGadaaakeaat0uy0HwzTfgDPnwy1egaryqtHrhAL1wy0L2yHvdaiqaacqWFBeIudaqhaaWcbaGaeeisaG0aaSbaaWqaaiabdQgaQbqabaaaleaacqWGRbWAaaaaaa@3BB4@. If no word in ℋ
 MathType@MTEF@5@5@+=feaafiart1ev1aaatCvAUfKttLearuWrP9MDH5MBPbIqV92AaeXatLxBI9gBaebbnrfifHhDYfgasaacH8akY=wiFfYdH8Gipec8Eeeu0xXdbba9frFj0=OqFfea0dXdd9vqai=hGuQ8kuc9pgc9s8qqaq=dirpe0xb9q8qiLsFr0=vr0=vr0dc8meaabaqaciaacaGaaeqabaqabeGadaaakeaat0uy0HwzTfgDPnwy1egaryqtHrhAL1wy0L2yHvdaiqaacqWFlecsaaa@3762@ is a subword of another word in ℋ
 MathType@MTEF@5@5@+=feaafiart1ev1aaatCvAUfKttLearuWrP9MDH5MBPbIqV92AaeXatLxBI9gBaebbnrfifHhDYfgasaacH8akY=wiFfYdH8Gipec8Eeeu0xXdbba9frFj0=OqFfea0dXdd9vqai=hGuQ8kuc9pgc9s8qqaq=dirpe0xb9q8qiLsFr0=vr0=vr0dc8meaabaqaciaacaGaaeqabaqabeGadaaakeaat0uy0HwzTfgDPnwy1egaryqtHrhAL1wy0L2yHvdaiqaacqWFlecsaaa@3762@, the probability **P **(*L*_*n *_(ℋ
 MathType@MTEF@5@5@+=feaafiart1ev1aaatCvAUfKttLearuWrP9MDH5MBPbIqV92AaeXatLxBI9gBaebbnrfifHhDYfgasaacH8akY=wiFfYdH8Gipec8Eeeu0xXdbba9frFj0=OqFfea0dXdd9vqai=hGuQ8kuc9pgc9s8qqaq=dirpe0xb9q8qiLsFr0=vr0=vr0dc8meaabaqaciaacaGaaeqabaqabeGadaaakeaat0uy0HwzTfgDPnwy1egaryqtHrhAL1wy0L2yHvdaiqaacqWFlecsaaa@3762@; *k*)) to find at least *k *occurrences of words from ℋ
 MathType@MTEF@5@5@+=feaafiart1ev1aaatCvAUfKttLearuWrP9MDH5MBPbIqV92AaeXatLxBI9gBaebbnrfifHhDYfgasaacH8akY=wiFfYdH8Gipec8Eeeu0xXdbba9frFj0=OqFfea0dXdd9vqai=hGuQ8kuc9pgc9s8qqaq=dirpe0xb9q8qiLsFr0=vr0=vr0dc8meaabaqaciaacaGaaeqabaqabeGadaaakeaat0uy0HwzTfgDPnwy1egaryqtHrhAL1wy0L2yHvdaiqaacqWFlecsaaa@3762@ in a random text of length *n *satisfies

P(Ln(ℋ;k))=∑p≤n∑Hj∈ℋrjk(p)
 MathType@MTEF@5@5@+=feaafiart1ev1aaatCvAUfKttLearuWrP9MDH5MBPbIqV92AaeXatLxBI9gBaebbnrfifHhDYfgasaacH8akY=wiFfYdH8Gipec8Eeeu0xXdbba9frFj0=OqFfea0dXdd9vqai=hGuQ8kuc9pgc9s8qqaq=dirpe0xb9q8qiLsFr0=vr0=vr0dc8meaabaqaciaacaGaaeqabaqabeGadaaakeaaieqacqWFqbaucqGGOaakcqWGmbatdaWgaaWcbaGaemOBa4gabeaakiabcIcaOmrtHrhAL1wy0L2yHvtyaeHbnfgDOvwBHrxAJfwnaGabaiab+TqiijabcUda7iabdUgaRjabcMcaPiabcMcaPiabg2da9maaqafabaWaaabuaeaacqWGYbGCdaqhaaWcbaGaemOAaOgabaGaem4AaSgaaOGaeiikaGIaemiCaaNaeiykaKcaleaacqqGibasdaWgaaadbaGaemOAaOgabeaaliabgIGiolab+Tqiibqab0GaeyyeIuoaaSqaaiabdchaWjabgsMiJkabd6gaUbqab0GaeyyeIuoaaaa@577F@

Therefore, one tries to compute the sequence of (rjk
 MathType@MTEF@5@5@+=feaafiart1ev1aaatCvAUfKttLearuWrP9MDH5MBPbIqV92AaeXatLxBI9gBaebbnrfifHhDYfgasaacH8akY=wiFfYdH8Gipec8Eeeu0xXdbba9frFj0=OqFfea0dXdd9vqai=hGuQ8kuc9pgc9s8qqaq=dirpe0xb9q8qiLsFr0=vr0=vr0dc8meaabaqaciaacaGaaeqabaqabeGadaaakeaacqWGYbGCdaqhaaWcbaGaemOAaOgabaGaem4AaSgaaaaa@3102@ (*p*)) values.

### Linear induction

In the first class of methods [43-46], one computes, implicitly or explicitly, probabilities **P **(*L*_*n *_(ℋ
 MathType@MTEF@5@5@+=feaafiart1ev1aaatCvAUfKttLearuWrP9MDH5MBPbIqV92AaeXatLxBI9gBaebbnrfifHhDYfgasaacH8akY=wiFfYdH8Gipec8Eeeu0xXdbba9frFj0=OqFfea0dXdd9vqai=hGuQ8kuc9pgc9s8qqaq=dirpe0xb9q8qiLsFr0=vr0=vr0dc8meaabaqaciaacaGaaeqabaqabeGadaaakeaat0uy0HwzTfgDPnwy1egaryqtHrhAL1wy0L2yHvdaiqaacqWFlecsaaa@3762@; *k*)) up to a given text length *n*. Such methods are intrinsically linear in *n*. In [43-46] one relies on a recurrence relation on rjk
 MathType@MTEF@5@5@+=feaafiart1ev1aaatCvAUfKttLearuWrP9MDH5MBPbIqV92AaeXatLxBI9gBaebbnrfifHhDYfgasaacH8akY=wiFfYdH8Gipec8Eeeu0xXdbba9frFj0=OqFfea0dXdd9vqai=hGuQ8kuc9pgc9s8qqaq=dirpe0xb9q8qiLsFr0=vr0=vr0dc8meaabaqaciaacaGaaeqabaqabeGadaaakeaacqWGYbGCdaqhaaWcbaGaemOAaOgabaGaem4AaSgaaaaa@3102@ (*n*) that extends the one originally given in [47]. Typically, one step will cost *O *(|ℋ
 MathType@MTEF@5@5@+=feaafiart1ev1aaatCvAUfKttLearuWrP9MDH5MBPbIqV92AaeXatLxBI9gBaebbnrfifHhDYfgasaacH8akY=wiFfYdH8Gipec8Eeeu0xXdbba9frFj0=OqFfea0dXdd9vqai=hGuQ8kuc9pgc9s8qqaq=dirpe0xb9q8qiLsFr0=vr0=vr0dc8meaabaqaciaacaGaaeqabaqabeGadaaakeaat0uy0HwzTfgDPnwy1egaryqtHrhAL1wy0L2yHvdaiqaacqWFlecsaaa@3762@|*m*), where ℋ
 MathType@MTEF@5@5@+=feaafiart1ev1aaatCvAUfKttLearuWrP9MDH5MBPbIqV92AaeXatLxBI9gBaebbnrfifHhDYfgasaacH8akY=wiFfYdH8Gipec8Eeeu0xXdbba9frFj0=OqFfea0dXdd9vqai=hGuQ8kuc9pgc9s8qqaq=dirpe0xb9q8qiLsFr0=vr0=vr0dc8meaabaqaciaacaGaaeqabaqabeGadaaakeaat0uy0HwzTfgDPnwy1egaryqtHrhAL1wy0L2yHvdaiqaacqWFlecsaaa@3762@ is a set of words of length *m *and |ℋ
 MathType@MTEF@5@5@+=feaafiart1ev1aaatCvAUfKttLearuWrP9MDH5MBPbIqV92AaeXatLxBI9gBaebbnrfifHhDYfgasaacH8akY=wiFfYdH8Gipec8Eeeu0xXdbba9frFj0=OqFfea0dXdd9vqai=hGuQ8kuc9pgc9s8qqaq=dirpe0xb9q8qiLsFr0=vr0=vr0dc8meaabaqaciaacaGaaeqabaqabeGadaaakeaat0uy0HwzTfgDPnwy1egaryqtHrhAL1wy0L2yHvdaiqaacqWFlecsaaa@3762@| is its cardinality. Time complexity is *O *(*n*|ℋ
 MathType@MTEF@5@5@+=feaafiart1ev1aaatCvAUfKttLearuWrP9MDH5MBPbIqV92AaeXatLxBI9gBaebbnrfifHhDYfgasaacH8akY=wiFfYdH8Gipec8Eeeu0xXdbba9frFj0=OqFfea0dXdd9vqai=hGuQ8kuc9pgc9s8qqaq=dirpe0xb9q8qiLsFr0=vr0=vr0dc8meaabaqaciaacaGaaeqabaqabeGadaaakeaat0uy0HwzTfgDPnwy1egaryqtHrhAL1wy0L2yHvdaiqaacqWFlecsaaa@3762@|*m*) and, relying on a combinatorial property, [44] achieves optimal space complexity *O *(|ℋ
 MathType@MTEF@5@5@+=feaafiart1ev1aaatCvAUfKttLearuWrP9MDH5MBPbIqV92AaeXatLxBI9gBaebbnrfifHhDYfgasaacH8akY=wiFfYdH8Gipec8Eeeu0xXdbba9frFj0=OqFfea0dXdd9vqai=hGuQ8kuc9pgc9s8qqaq=dirpe0xb9q8qiLsFr0=vr0=vr0dc8meaabaqaciaacaGaaeqabaqabeGadaaakeaat0uy0HwzTfgDPnwy1egaryqtHrhAL1wy0L2yHvdaiqaacqWFlecsaaa@3762@| log |ℋ
 MathType@MTEF@5@5@+=feaafiart1ev1aaatCvAUfKttLearuWrP9MDH5MBPbIqV92AaeXatLxBI9gBaebbnrfifHhDYfgasaacH8akY=wiFfYdH8Gipec8Eeeu0xXdbba9frFj0=OqFfea0dXdd9vqai=hGuQ8kuc9pgc9s8qqaq=dirpe0xb9q8qiLsFr0=vr0=vr0dc8meaabaqaciaacaGaaeqabaqabeGadaaakeaat0uy0HwzTfgDPnwy1egaryqtHrhAL1wy0L2yHvdaiqaacqWFlecsaaa@3762@|*m*). However the authors of [44] do not consider several motifs occurrences and restrict themselves to the Bernoulli model. The authors of [43] consider the Markov model, still using one motif for TFBS.

### Algebraic Formulae

In a second class of methods [47-52], a preprocessing computes *generating functions*

rjk(z)=∑nrjk(n)zn.
 MathType@MTEF@5@5@+=feaafiart1ev1aaatCvAUfKttLearuWrP9MDH5MBPbIqV92AaeXatLxBI9gBaebbnrfifHhDYfgasaacH8akY=wiFfYdH8Gipec8Eeeu0xXdbba9frFj0=OqFfea0dXdd9vqai=hGuQ8kuc9pgc9s8qqaq=dirpe0xb9q8qiLsFr0=vr0=vr0dc8meaabaqaciaacaGaaeqabaqabeGadaaakeaacqWGYbGCdaqhaaWcbaGaemOAaOgabaGaem4AaSgaaOGaeiikaGIaemOEaONaeiykaKIaeyypa0ZaaabuaeaacqWGYbGCdaqhaaWcbaGaemOAaOgabaGaem4AaSgaaOGaeiikaGIaemOBa4MaeiykaKIaemOEaO3aaWbaaSqabeaacqWGUbGBaaaabaGaemOBa4gabeqdcqGHris5aOGaeiOla4caaa@4432@

In a second step, probabilities **P **(*L*_*n *_(ℋ
 MathType@MTEF@5@5@+=feaafiart1ev1aaatCvAUfKttLearuWrP9MDH5MBPbIqV92AaeXatLxBI9gBaebbnrfifHhDYfgasaacH8akY=wiFfYdH8Gipec8Eeeu0xXdbba9frFj0=OqFfea0dXdd9vqai=hGuQ8kuc9pgc9s8qqaq=dirpe0xb9q8qiLsFr0=vr0=vr0dc8meaabaqaciaacaGaaeqabaqabeGadaaakeaat0uy0HwzTfgDPnwy1egaryqtHrhAL1wy0L2yHvdaiqaacqWFlecsaaa@3762@; *k*)) are either extracted from the generating function or approximated.

In [49,53], rjk
 MathType@MTEF@5@5@+=feaafiart1ev1aaatCvAUfKttLearuWrP9MDH5MBPbIqV92AaeXatLxBI9gBaebbnrfifHhDYfgasaacH8akY=wiFfYdH8Gipec8Eeeu0xXdbba9frFj0=OqFfea0dXdd9vqai=hGuQ8kuc9pgc9s8qqaq=dirpe0xb9q8qiLsFr0=vr0=vr0dc8meaabaqaciaacaGaaeqabaqabeGadaaakeaacqWGYbGCdaqhaaWcbaGaemOAaOgabaGaem4AaSgaaaaa@3102@ (*z*) are the solutions of a system of equations. To derive these equations, the authors build an automaton that recognizes these languages ℛHjk
 MathType@MTEF@5@5@+=feaafiart1ev1aaatCvAUfKttLearuWrP9MDH5MBPbIqV92AaeXatLxBI9gBaebbnrfifHhDYfgasaacH8akY=wiFfYdH8Gipec8Eeeu0xXdbba9frFj0=OqFfea0dXdd9vqai=hGuQ8kuc9pgc9s8qqaq=dirpe0xb9q8qiLsFr0=vr0=vr0dc8meaabaqaciaacaGaaeqabaqabeGadaaakeaat0uy0HwzTfgDPnwy1egaryqtHrhAL1wy0L2yHvdaiqaacqWFBeIudaqhaaWcbaGaeeisaG0aaSbaaWqaaiabdQgaQbqabaaaleaacqWGRbWAaaaaaa@3BB4@ (one can prove that they are regular).

A language approach [50] or an induction [48] leads to a formal expression that depends on the words overlaps. The main drawback is that these methods need to compute the determinant of a matrix of polynomials with a huge dimension, e.g. *O *(|ℋ
 MathType@MTEF@5@5@+=feaafiart1ev1aaatCvAUfKttLearuWrP9MDH5MBPbIqV92AaeXatLxBI9gBaebbnrfifHhDYfgasaacH8akY=wiFfYdH8Gipec8Eeeu0xXdbba9frFj0=OqFfea0dXdd9vqai=hGuQ8kuc9pgc9s8qqaq=dirpe0xb9q8qiLsFr0=vr0=vr0dc8meaabaqaciaacaGaaeqabaqabeGadaaakeaat0uy0HwzTfgDPnwy1egaryqtHrhAL1wy0L2yHvdaiqaacqWFlecsaaa@3762@|). This *O *(|ℋ
 MathType@MTEF@5@5@+=feaafiart1ev1aaatCvAUfKttLearuWrP9MDH5MBPbIqV92AaeXatLxBI9gBaebbnrfifHhDYfgasaacH8akY=wiFfYdH8Gipec8Eeeu0xXdbba9frFj0=OqFfea0dXdd9vqai=hGuQ8kuc9pgc9s8qqaq=dirpe0xb9q8qiLsFr0=vr0=vr0dc8meaabaqaciaacaGaaeqabaqabeGadaaakeaat0uy0HwzTfgDPnwy1egaryqtHrhAL1wy0L2yHvdaiqaacqWFlecsaaa@3762@|^2^) *symbolic computation *may be more expensive than the extraction step or the linear computation above, that involve *arithmetic operations *on real numbers.

When the preprocessing step is achievable, the extraction step is amenable to the solution of a linear recurrence of degree *m*|ℋ
 MathType@MTEF@5@5@+=feaafiart1ev1aaatCvAUfKttLearuWrP9MDH5MBPbIqV92AaeXatLxBI9gBaebbnrfifHhDYfgasaacH8akY=wiFfYdH8Gipec8Eeeu0xXdbba9frFj0=OqFfea0dXdd9vqai=hGuQ8kuc9pgc9s8qqaq=dirpe0xb9q8qiLsFr0=vr0=vr0dc8meaabaqaciaacaGaaeqabaqabeGadaaakeaat0uy0HwzTfgDPnwy1egaryqtHrhAL1wy0L2yHvdaiqaacqWFlecsaaa@3762@|; therefore, its complexity is *O *(*m*|ℋ
 MathType@MTEF@5@5@+=feaafiart1ev1aaatCvAUfKttLearuWrP9MDH5MBPbIqV92AaeXatLxBI9gBaebbnrfifHhDYfgasaacH8akY=wiFfYdH8Gipec8Eeeu0xXdbba9frFj0=OqFfea0dXdd9vqai=hGuQ8kuc9pgc9s8qqaq=dirpe0xb9q8qiLsFr0=vr0=vr0dc8meaabaqaciaacaGaaeqabaqabeGadaaakeaat0uy0HwzTfgDPnwy1egaryqtHrhAL1wy0L2yHvdaiqaacqWFlecsaaa@3762@|*n*) and a classical optimization yields *O *(*m*|ℋ
 MathType@MTEF@5@5@+=feaafiart1ev1aaatCvAUfKttLearuWrP9MDH5MBPbIqV92AaeXatLxBI9gBaebbnrfifHhDYfgasaacH8akY=wiFfYdH8Gipec8Eeeu0xXdbba9frFj0=OqFfea0dXdd9vqai=hGuQ8kuc9pgc9s8qqaq=dirpe0xb9q8qiLsFr0=vr0=vr0dc8meaabaqaciaacaGaaeqabaqabeGadaaakeaat0uy0HwzTfgDPnwy1egaryqtHrhAL1wy0L2yHvdaiqaacqWFlecsaaa@3762@| log *n*). There exists some good implementations that are numerically stable. One may cite the REGEXPCOUNT [54] or EXCEP [55] programs that rely on Fast Fourier Transform.

Finally, approximations are available, the computation of which is constant with respect to *n*, but not to ℋ
 MathType@MTEF@5@5@+=feaafiart1ev1aaatCvAUfKttLearuWrP9MDH5MBPbIqV92AaeXatLxBI9gBaebbnrfifHhDYfgasaacH8akY=wiFfYdH8Gipec8Eeeu0xXdbba9frFj0=OqFfea0dXdd9vqai=hGuQ8kuc9pgc9s8qqaq=dirpe0xb9q8qiLsFr0=vr0=vr0dc8meaabaqaciaacaGaaeqabaqabeGadaaakeaat0uy0HwzTfgDPnwy1egaryqtHrhAL1wy0L2yHvdaiqaacqWFlecsaaa@3762@. One approach is the compound Poisson approximation [56], but this approximation is not precise enough [57]. Asymptotic results can also be derived from the algebraic formulae above [44,58], not needing an explicit expression for rjk
 MathType@MTEF@5@5@+=feaafiart1ev1aaatCvAUfKttLearuWrP9MDH5MBPbIqV92AaeXatLxBI9gBaebbnrfifHhDYfgasaacH8akY=wiFfYdH8Gipec8Eeeu0xXdbba9frFj0=OqFfea0dXdd9vqai=hGuQ8kuc9pgc9s8qqaq=dirpe0xb9q8qiLsFr0=vr0=vr0dc8meaabaqaciaacaGaaeqabaqabeGadaaakeaacqWGYbGCdaqhaaWcbaGaemOAaOgabaGaem4AaSgaaaaa@3102@ (*z*), and therefore avoiding the expensive determinant computation. Time complexity, typically, is the one for computing all possible overlaps, that is approximately *O *(|ℋ
 MathType@MTEF@5@5@+=feaafiart1ev1aaatCvAUfKttLearuWrP9MDH5MBPbIqV92AaeXatLxBI9gBaebbnrfifHhDYfgasaacH8akY=wiFfYdH8Gipec8Eeeu0xXdbba9frFj0=OqFfea0dXdd9vqai=hGuQ8kuc9pgc9s8qqaq=dirpe0xb9q8qiLsFr0=vr0=vr0dc8meaabaqaciaacaGaaeqabaqabeGadaaakeaat0uy0HwzTfgDPnwy1egaryqtHrhAL1wy0L2yHvdaiqaacqWFlecsaaa@3762@|^2^). This yields extremely precise results when the expectation of the number of occurrences, *nP *(H) is very small [59] or close to 1 [51] (the case studied the most often). Case *nP *(H) ~2 is achieved in [60]. Nevertheless, extension to larger values of *k *or multioccurrences and multisets is still open.

## Methods

Here we consider in detail the approach we suggest.

A motif assigned to a TF is a finite set of words ℋ
 MathType@MTEF@5@5@+=feaafiart1ev1aaatCvAUfKttLearuWrP9MDH5MBPbIqV92AaeXatLxBI9gBaebbnrfifHhDYfgasaacH8akY=wiFfYdH8Gipec8Eeeu0xXdbba9frFj0=OqFfea0dXdd9vqai=hGuQ8kuc9pgc9s8qqaq=dirpe0xb9q8qiLsFr0=vr0=vr0dc8meaabaqaciaacaGaaeqabaqabeGadaaakeaat0uy0HwzTfgDPnwy1egaryqtHrhAL1wy0L2yHvdaiqaacqWFlecsaaa@3762@ = (H_1_, ..., H_r_) where each word represents one putative TF binding site in DNA. Note that words in motif can generally be of different lengths. However, no word from ℋ
 MathType@MTEF@5@5@+=feaafiart1ev1aaatCvAUfKttLearuWrP9MDH5MBPbIqV92AaeXatLxBI9gBaebbnrfifHhDYfgasaacH8akY=wiFfYdH8Gipec8Eeeu0xXdbba9frFj0=OqFfea0dXdd9vqai=hGuQ8kuc9pgc9s8qqaq=dirpe0xb9q8qiLsFr0=vr0=vr0dc8meaabaqaciaacaGaaeqabaqabeGadaaakeaat0uy0HwzTfgDPnwy1egaryqtHrhAL1wy0L2yHvdaiqaacqWFlecsaaa@3762@ can contain another word from ℋ
 MathType@MTEF@5@5@+=feaafiart1ev1aaatCvAUfKttLearuWrP9MDH5MBPbIqV92AaeXatLxBI9gBaebbnrfifHhDYfgasaacH8akY=wiFfYdH8Gipec8Eeeu0xXdbba9frFj0=OqFfea0dXdd9vqai=hGuQ8kuc9pgc9s8qqaq=dirpe0xb9q8qiLsFr0=vr0=vr0dc8meaabaqaciaacaGaaeqabaqabeGadaaakeaat0uy0HwzTfgDPnwy1egaryqtHrhAL1wy0L2yHvdaiqaacqWFlecsaaa@3762@ as a substring. We consider, as an occurrence of motif ℋ
 MathType@MTEF@5@5@+=feaafiart1ev1aaatCvAUfKttLearuWrP9MDH5MBPbIqV92AaeXatLxBI9gBaebbnrfifHhDYfgasaacH8akY=wiFfYdH8Gipec8Eeeu0xXdbba9frFj0=OqFfea0dXdd9vqai=hGuQ8kuc9pgc9s8qqaq=dirpe0xb9q8qiLsFr0=vr0=vr0dc8meaabaqaciaacaGaaeqabaqabeGadaaakeaat0uy0HwzTfgDPnwy1egaryqtHrhAL1wy0L2yHvdaiqaacqWFlecsaaa@3762@ in text *T*, any occurrence of any word ℋ
 MathType@MTEF@5@5@+=feaafiart1ev1aaatCvAUfKttLearuWrP9MDH5MBPbIqV92AaeXatLxBI9gBaebbnrfifHhDYfgasaacH8akY=wiFfYdH8Gipec8Eeeu0xXdbba9frFj0=OqFfea0dXdd9vqai=hGuQ8kuc9pgc9s8qqaq=dirpe0xb9q8qiLsFr0=vr0=vr0dc8meaabaqaciaacaGaaeqabaqabeGadaaakeaat0uy0HwzTfgDPnwy1egaryqtHrhAL1wy0L2yHvdaiqaacqWFlecsaaa@3762@_*j *_∈ ℋ
 MathType@MTEF@5@5@+=feaafiart1ev1aaatCvAUfKttLearuWrP9MDH5MBPbIqV92AaeXatLxBI9gBaebbnrfifHhDYfgasaacH8akY=wiFfYdH8Gipec8Eeeu0xXdbba9frFj0=OqFfea0dXdd9vqai=hGuQ8kuc9pgc9s8qqaq=dirpe0xb9q8qiLsFr0=vr0=vr0dc8meaabaqaciaacaGaaeqabaqabeGadaaakeaat0uy0HwzTfgDPnwy1egaryqtHrhAL1wy0L2yHvdaiqaacqWFlecsaaa@3762@ in *T*. Below all texts and words in motifs are sequences on a given alphabet Σ.

Let (ℋ1,...,ℋs
 MathType@MTEF@5@5@+=feaafiart1ev1aaatCvAUfKttLearuWrP9MDH5MBPbIqV92AaeXatLxBI9gBaebbnrfifHhDYfgasaacH8akY=wiFfYdH8Gipec8Eeeu0xXdbba9frFj0=OqFfea0dXdd9vqai=hGuQ8kuc9pgc9s8qqaq=dirpe0xb9q8qiLsFr0=vr0=vr0dc8meaabaqaciaacaGaaeqabaqabeGadaaakeaat0uy0HwzTfgDPnwy1egaryqtHrhAL1wy0L2yHvdaiqaacqWFlecsdaWgaaWcbaGaeGymaedabeaakiabcYcaSiabc6caUiabc6caUiabc6caUiabcYcaSiab=TqiinaaBaaaleaacqWGZbWCaeqaaaaa@3F88@) be *s *different motifs. Our objective is to calculate the probability (*p*-value) that motifs (ℋ1,...,ℋs
 MathType@MTEF@5@5@+=feaafiart1ev1aaatCvAUfKttLearuWrP9MDH5MBPbIqV92AaeXatLxBI9gBaebbnrfifHhDYfgasaacH8akY=wiFfYdH8Gipec8Eeeu0xXdbba9frFj0=OqFfea0dXdd9vqai=hGuQ8kuc9pgc9s8qqaq=dirpe0xb9q8qiLsFr0=vr0=vr0dc8meaabaqaciaacaGaaeqabaqabeGadaaakeaat0uy0HwzTfgDPnwy1egaryqtHrhAL1wy0L2yHvdaiqaacqWFlecsdaWgaaWcbaGaeGymaedabeaakiabcYcaSiabc6caUiabc6caUiabc6caUiabcYcaSiab=TqiinaaBaaaleaacqWGZbWCaeqaaaaa@3F88@) have respectively at least (*k*_1_, ..., *k*_*s*_) possibly overlapping occurrences in a random text *T*_*n*_.

To be more precise, there is a probability distribution defined on the set Σ^*n *^of all texts of length *n *in the alphabet Σ; the most widely used models are random Bernoulli trials and a Markov model of order *K*. Denote as *L*_*n *_(ℋ1,...,ℋs
 MathType@MTEF@5@5@+=feaafiart1ev1aaatCvAUfKttLearuWrP9MDH5MBPbIqV92AaeXatLxBI9gBaebbnrfifHhDYfgasaacH8akY=wiFfYdH8Gipec8Eeeu0xXdbba9frFj0=OqFfea0dXdd9vqai=hGuQ8kuc9pgc9s8qqaq=dirpe0xb9q8qiLsFr0=vr0=vr0dc8meaabaqaciaacaGaaeqabaqabeGadaaakeaat0uy0HwzTfgDPnwy1egaryqtHrhAL1wy0L2yHvdaiqaacqWFlecsdaWgaaWcbaGaeGymaedabeaakiabcYcaSiabc6caUiabc6caUiabc6caUiabcYcaSiab=TqiinaaBaaaleaacqWGZbWCaeqaaaaa@3F88@; *k*_1_, ..., *k*_*s*_) the set of all texts of length *n *containing at least *k*_*i *_possibly overlapping occurrences of each motif ℋ
 MathType@MTEF@5@5@+=feaafiart1ev1aaatCvAUfKttLearuWrP9MDH5MBPbIqV92AaeXatLxBI9gBaebbnrfifHhDYfgasaacH8akY=wiFfYdH8Gipec8Eeeu0xXdbba9frFj0=OqFfea0dXdd9vqai=hGuQ8kuc9pgc9s8qqaq=dirpe0xb9q8qiLsFr0=vr0=vr0dc8meaabaqaciaacaGaaeqabaqabeGadaaakeaat0uy0HwzTfgDPnwy1egaryqtHrhAL1wy0L2yHvdaiqaacqWFlecsaaa@3762@_*i*_; *i *= 1, ..., *s*. Then the desired *p*-value is the probability **P **(*L*_*n *_(ℋ1,...,ℋs
 MathType@MTEF@5@5@+=feaafiart1ev1aaatCvAUfKttLearuWrP9MDH5MBPbIqV92AaeXatLxBI9gBaebbnrfifHhDYfgasaacH8akY=wiFfYdH8Gipec8Eeeu0xXdbba9frFj0=OqFfea0dXdd9vqai=hGuQ8kuc9pgc9s8qqaq=dirpe0xb9q8qiLsFr0=vr0=vr0dc8meaabaqaciaacaGaaeqabaqabeGadaaakeaat0uy0HwzTfgDPnwy1egaryqtHrhAL1wy0L2yHvdaiqaacqWFlecsdaWgaaWcbaGaeGymaedabeaakiabcYcaSiabc6caUiabc6caUiabc6caUiabcYcaSiab=TqiinaaBaaaleaacqWGZbWCaeqaaaaa@3F88@; *k*_1_, ..., *k*_*s*_)) of the set *L*_*n *_(ℋ1,...,ℋs
 MathType@MTEF@5@5@+=feaafiart1ev1aaatCvAUfKttLearuWrP9MDH5MBPbIqV92AaeXatLxBI9gBaebbnrfifHhDYfgasaacH8akY=wiFfYdH8Gipec8Eeeu0xXdbba9frFj0=OqFfea0dXdd9vqai=hGuQ8kuc9pgc9s8qqaq=dirpe0xb9q8qiLsFr0=vr0=vr0dc8meaabaqaciaacaGaaeqabaqabeGadaaakeaat0uy0HwzTfgDPnwy1egaryqtHrhAL1wy0L2yHvdaiqaacqWFlecsdaWgaaWcbaGaeGymaedabeaakiabcYcaSiabc6caUiabc6caUiabc6caUiabcYcaSiab=TqiinaaBaaaleaacqWGZbWCaeqaaaaa@3F88@; *k*_1_, ..., *k*_*s*_) with respect to the given probability distribution on Σ^*n*^.

Our approach to the calculation of this *p*-value is similar to that published in [61], which was used there to calculate seed sensitivity in local alignment search. The approach exploits the fact that the algorithm of Aho and Corasick [62] can be modified to efficiently determine whether a given text belongs to the set *L*_*n *_(ℋ1,...,ℋs
 MathType@MTEF@5@5@+=feaafiart1ev1aaatCvAUfKttLearuWrP9MDH5MBPbIqV92AaeXatLxBI9gBaebbnrfifHhDYfgasaacH8akY=wiFfYdH8Gipec8Eeeu0xXdbba9frFj0=OqFfea0dXdd9vqai=hGuQ8kuc9pgc9s8qqaq=dirpe0xb9q8qiLsFr0=vr0=vr0dc8meaabaqaciaacaGaaeqabaqabeGadaaakeaat0uy0HwzTfgDPnwy1egaryqtHrhAL1wy0L2yHvdaiqaacqWFlecsdaWgaaWcbaGaeGymaedabeaakiabcYcaSiabc6caUiabc6caUiabc6caUiabcYcaSiab=TqiinaaBaaaleaacqWGZbWCaeqaaaaa@3F88@; *k*_1_, ..., *k*_*s*_) or not. Ideas published in [61] and [62] can be adopted to compute the probability **P **(*L*_*n *_(ℋ1,...,ℋs
 MathType@MTEF@5@5@+=feaafiart1ev1aaatCvAUfKttLearuWrP9MDH5MBPbIqV92AaeXatLxBI9gBaebbnrfifHhDYfgasaacH8akY=wiFfYdH8Gipec8Eeeu0xXdbba9frFj0=OqFfea0dXdd9vqai=hGuQ8kuc9pgc9s8qqaq=dirpe0xb9q8qiLsFr0=vr0=vr0dc8meaabaqaciaacaGaaeqabaqabeGadaaakeaat0uy0HwzTfgDPnwy1egaryqtHrhAL1wy0L2yHvdaiqaacqWFlecsdaWgaaWcbaGaeGymaedabeaakiabcYcaSiabc6caUiabc6caUiabc6caUiabcYcaSiab=TqiinaaBaaaleaacqWGZbWCaeqaaaaa@3F88@; *k*_1_, ..., *k*_*s*_)) that the random text *T*_*n *_∈ Σ^*n *^belongs to the set *L*_*n *_(ℋ1,...,ℋs
 MathType@MTEF@5@5@+=feaafiart1ev1aaatCvAUfKttLearuWrP9MDH5MBPbIqV92AaeXatLxBI9gBaebbnrfifHhDYfgasaacH8akY=wiFfYdH8Gipec8Eeeu0xXdbba9frFj0=OqFfea0dXdd9vqai=hGuQ8kuc9pgc9s8qqaq=dirpe0xb9q8qiLsFr0=vr0=vr0dc8meaabaqaciaacaGaaeqabaqabeGadaaakeaat0uy0HwzTfgDPnwy1egaryqtHrhAL1wy0L2yHvdaiqaacqWFlecsdaWgaaWcbaGaeGymaedabeaakiabcYcaSiabc6caUiabc6caUiabc6caUiabcYcaSiab=TqiinaaBaaaleaacqWGZbWCaeqaaaaa@3F88@; *k*_1_, ..., *k*_*s*_).

We start from the simplest case of one motif ℋ
 MathType@MTEF@5@5@+=feaafiart1ev1aaatCvAUfKttLearuWrP9MDH5MBPbIqV92AaeXatLxBI9gBaebbnrfifHhDYfgasaacH8akY=wiFfYdH8Gipec8Eeeu0xXdbba9frFj0=OqFfea0dXdd9vqai=hGuQ8kuc9pgc9s8qqaq=dirpe0xb9q8qiLsFr0=vr0=vr0dc8meaabaqaciaacaGaaeqabaqabeGadaaakeaat0uy0HwzTfgDPnwy1egaryqtHrhAL1wy0L2yHvdaiqaacqWFlecsaaa@3762@ for which we calculate the probability **P **(*L*_*n *_(ℋ
 MathType@MTEF@5@5@+=feaafiart1ev1aaatCvAUfKttLearuWrP9MDH5MBPbIqV92AaeXatLxBI9gBaebbnrfifHhDYfgasaacH8akY=wiFfYdH8Gipec8Eeeu0xXdbba9frFj0=OqFfea0dXdd9vqai=hGuQ8kuc9pgc9s8qqaq=dirpe0xb9q8qiLsFr0=vr0=vr0dc8meaabaqaciaacaGaaeqabaqabeGadaaakeaat0uy0HwzTfgDPnwy1egaryqtHrhAL1wy0L2yHvdaiqaacqWFlecsaaa@3762@; 1)) that text *T*_*n *_contains at least one occurrence of the motif with respect to a Bernoulli probability distribution. More complicated cases (arbitrary number of occurrences; arbitrary number of motifs; Markov distribution) will be discussed in the following sections.

### Construction of Aho-Corasick traversal

Aho and Corasick [62] have proposed the algorithm determining if a given text *T *contains an occurrence of a word from a given set ℋ
 MathType@MTEF@5@5@+=feaafiart1ev1aaatCvAUfKttLearuWrP9MDH5MBPbIqV92AaeXatLxBI9gBaebbnrfifHhDYfgasaacH8akY=wiFfYdH8Gipec8Eeeu0xXdbba9frFj0=OqFfea0dXdd9vqai=hGuQ8kuc9pgc9s8qqaq=dirpe0xb9q8qiLsFr0=vr0=vr0dc8meaabaqaciaacaGaaeqabaqabeGadaaakeaat0uy0HwzTfgDPnwy1egaryqtHrhAL1wy0L2yHvdaiqaacqWFlecsaaa@3762@. The basic data structure is a prefix tree which is a variant of the classical trie T(ℋ)
 MathType@MTEF@5@5@+=feaafiart1ev1aaatCvAUfKttLearuWrP9MDH5MBPbIqV92AaeXatLxBI9gBaebbnrfifHhDYfgasaacH8akY=wiFfYdH8Gipec8Eeeu0xXdbba9frFj0=OqFfea0dXdd9vqai=hGuQ8kuc9pgc9s8qqaq=dirpe0xb9q8qiLsFr0=vr0=vr0dc8meaabaqaciaacaGaaeqabaqabeGadaaakeaat0uy0HwzTfgDPnwy1egaryqtHrhAL1wy0L2yHvdaiqaacqWFtepvcqGGOaakcqWFlecscqGGPaqkaaa@3AF1@[42] that may be built on the set of words ℋ
 MathType@MTEF@5@5@+=feaafiart1ev1aaatCvAUfKttLearuWrP9MDH5MBPbIqV92AaeXatLxBI9gBaebbnrfifHhDYfgasaacH8akY=wiFfYdH8Gipec8Eeeu0xXdbba9frFj0=OqFfea0dXdd9vqai=hGuQ8kuc9pgc9s8qqaq=dirpe0xb9q8qiLsFr0=vr0=vr0dc8meaabaqaciaacaGaaeqabaqabeGadaaakeaat0uy0HwzTfgDPnwy1egaryqtHrhAL1wy0L2yHvdaiqaacqWFlecsaaa@3762@. Let Qℋ
 MathType@MTEF@5@5@+=feaafiart1ev1aaatCvAUfKttLearuWrP9MDH5MBPbIqV92AaeXatLxBI9gBaebbnrfifHhDYfgasaacH8akY=wiFfYdH8Gipec8Eeeu0xXdbba9frFj0=OqFfea0dXdd9vqai=hGuQ8kuc9pgc9s8qqaq=dirpe0xb9q8qiLsFr0=vr0=vr0dc8meaabaqaciaacaGaaeqabaqabeGadaaakeaacqWGrbqudaWgaaWcbaWenfgDOvwBHrxAJfwnHbqeg0uy0HwzTfgDPnwy1aaceaGae83cHGeabeaaaaa@38B9@ denote the set of prefixes of these words. In the following, we identify a word *q *∈ Qℋ
 MathType@MTEF@5@5@+=feaafiart1ev1aaatCvAUfKttLearuWrP9MDH5MBPbIqV92AaeXatLxBI9gBaebbnrfifHhDYfgasaacH8akY=wiFfYdH8Gipec8Eeeu0xXdbba9frFj0=OqFfea0dXdd9vqai=hGuQ8kuc9pgc9s8qqaq=dirpe0xb9q8qiLsFr0=vr0=vr0dc8meaabaqaciaacaGaaeqabaqabeGadaaakeaacqWGrbqudaWgaaWcbaWenfgDOvwBHrxAJfwnHbqeg0uy0HwzTfgDPnwy1aaceaGae83cHGeabeaaaaa@38B9@ with node *Node *(*q*) at the end of the branch labeled by *q*. In particular, the root is identified with the empty string *ε*. The length of a prefix is the depth of *Node *(*q*).

The classic Aho-Corasick algorithm is a tree traversal determined by a *transition function *δ:Qℋ×Σ→Qℋ
 MathType@MTEF@5@5@+=feaafiart1ev1aaatCvAUfKttLearuWrP9MDH5MBPbIqV92AaeXatLxBI9gBaebbnrfifHhDYfgasaacH8akY=wiFfYdH8Gipec8Eeeu0xXdbba9frFj0=OqFfea0dXdd9vqai=hGuQ8kuc9pgc9s8qqaq=dirpe0xb9q8qiLsFr0=vr0=vr0dc8meaabaqaciaacaGaaeqabaqabeGadaaakeaaiiGacqWF0oazcqGG6aGocqWGrbqudaWgaaWcbaWenfgDOvwBHrxAJfwnHbqeg0uy0HwzTfgDPnwy1aaceaGae43cHGeabeaakiabgEna0kabfo6atjabgkziUkabdgfarnaaBaaaleaacqGFlecsaeqaaaaa@4341@ defined as follows. For any pair (*p, a*) in Qℋ
 MathType@MTEF@5@5@+=feaafiart1ev1aaatCvAUfKttLearuWrP9MDH5MBPbIqV92AaeXatLxBI9gBaebbnrfifHhDYfgasaacH8akY=wiFfYdH8Gipec8Eeeu0xXdbba9frFj0=OqFfea0dXdd9vqai=hGuQ8kuc9pgc9s8qqaq=dirpe0xb9q8qiLsFr0=vr0=vr0dc8meaabaqaciaacaGaaeqabaqabeGadaaakeaacqWGrbqudaWgaaWcbaWenfgDOvwBHrxAJfwnHbqeg0uy0HwzTfgDPnwy1aaceaGae83cHGeabeaaaaa@38B9@ × Σ, *δ *(*p, a*) is the largest suffix of concatenation *pa *that belongs to Qℋ
 MathType@MTEF@5@5@+=feaafiart1ev1aaatCvAUfKttLearuWrP9MDH5MBPbIqV92AaeXatLxBI9gBaebbnrfifHhDYfgasaacH8akY=wiFfYdH8Gipec8Eeeu0xXdbba9frFj0=OqFfea0dXdd9vqai=hGuQ8kuc9pgc9s8qqaq=dirpe0xb9q8qiLsFr0=vr0=vr0dc8meaabaqaciaacaGaaeqabaqabeGadaaakeaacqWGrbqudaWgaaWcbaWenfgDOvwBHrxAJfwnHbqeg0uy0HwzTfgDPnwy1aaceaGae83cHGeabeaaaaa@38B9@. Remark that *δ *(*p, a*) = *pa *iff *pa *∈ Qℋ
 MathType@MTEF@5@5@+=feaafiart1ev1aaatCvAUfKttLearuWrP9MDH5MBPbIqV92AaeXatLxBI9gBaebbnrfifHhDYfgasaacH8akY=wiFfYdH8Gipec8Eeeu0xXdbba9frFj0=OqFfea0dXdd9vqai=hGuQ8kuc9pgc9s8qqaq=dirpe0xb9q8qiLsFr0=vr0=vr0dc8meaabaqaciaacaGaaeqabaqabeGadaaakeaacqWGrbqudaWgaaWcbaWenfgDOvwBHrxAJfwnHbqeg0uy0HwzTfgDPnwy1aaceaGae83cHGeabeaaaaa@38B9@.

Given a text *T *read from left to right, let *T *[*i*] denote the letter of *T *at position *i*. Let *q*_*i *_be the largest suffix in text *T*[1] ⋯ *T *[*i*] that belongs to Qℋ
 MathType@MTEF@5@5@+=feaafiart1ev1aaatCvAUfKttLearuWrP9MDH5MBPbIqV92AaeXatLxBI9gBaebbnrfifHhDYfgasaacH8akY=wiFfYdH8Gipec8Eeeu0xXdbba9frFj0=OqFfea0dXdd9vqai=hGuQ8kuc9pgc9s8qqaq=dirpe0xb9q8qiLsFr0=vr0=vr0dc8meaabaqaciaacaGaaeqabaqabeGadaaakeaacqWGrbqudaWgaaWcbaWenfgDOvwBHrxAJfwnHbqeg0uy0HwzTfgDPnwy1aaceaGae83cHGeabeaaaaa@38B9@. The sequence of nodes visited during the traversal are defined by words *q*_*i *_that satisfy the inductive relationship

∀*i *≥ 0, *q*_*i*+1 _= *δ *(*q*_*i*_, *T *[*i *+ 1]),

with the initial condition *q*_0 _= *ε*.

**Figure 1:**
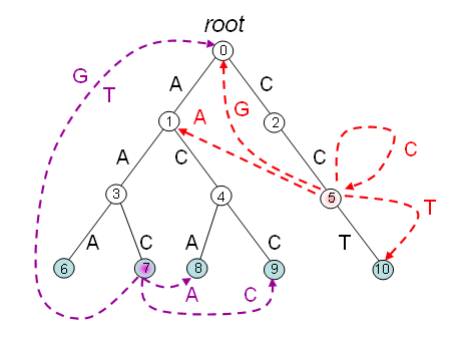
**Tree T(ℋ)
 MathType@MTEF@5@5@+=feaafiart1ev1aaatCvAUfKttLearuWrP9MDH5MBPbIqV92AaeXatLxBI9gBaebbnrfifHhDYfgasaacH8akY=wiFfYdH8Gipec8Eeeu0xXdbba9frFj0=OqFfea0dXdd9vqai=hGuQ8kuc9pgc9s8qqaq=dirpe0xb9q8qiLsFr0=vr0=vr0dc8meaabaqaciaacaGaaeqabaqabeGadaaakeaat0uy0HwzTfgDPnwy1egaryqtHrhAL1wy0L2yHvdaiqaacqWFtepvcqGGOaakcqWFlecscqGGPaqkaaa@3AF1@ for the set ℋ
 MathType@MTEF@5@5@+=feaafiart1ev1aaatCvAUfKttLearuWrP9MDH5MBPbIqV92AaeXatLxBI9gBaebbnrfifHhDYfgasaacH8akY=wiFfYdH8Gipec8Eeeu0xXdbba9frFj0=OqFfea0dXdd9vqai=hGuQ8kuc9pgc9s8qqaq=dirpe0xb9q8qiLsFr0=vr0=vr0dc8meaabaqaciaacaGaaeqabaqabeGadaaakeaat0uy0HwzTfgDPnwy1egaryqtHrhAL1wy0L2yHvdaiqaacqWFlecsaaa@3762@ = {aaa, aac, aca, acc, cct} with dashed links for *δ *function**. Tree T(ℋ)
 MathType@MTEF@5@5@+=feaafiart1ev1aaatCvAUfKttLearuWrP9MDH5MBPbIqV92AaeXatLxBI9gBaebbnrfifHhDYfgasaacH8akY=wiFfYdH8Gipec8Eeeu0xXdbba9frFj0=OqFfea0dXdd9vqai=hGuQ8kuc9pgc9s8qqaq=dirpe0xb9q8qiLsFr0=vr0=vr0dc8meaabaqaciaacaGaaeqabaqabeGadaaakeaat0uy0HwzTfgDPnwy1egaryqtHrhAL1wy0L2yHvdaiqaacqWFtepvcqGGOaakcqWFlecscqGGPaqkaaa@3AF1@ for the set ℋ
 MathType@MTEF@5@5@+=feaafiart1ev1aaatCvAUfKttLearuWrP9MDH5MBPbIqV92AaeXatLxBI9gBaebbnrfifHhDYfgasaacH8akY=wiFfYdH8Gipec8Eeeu0xXdbba9frFj0=OqFfea0dXdd9vqai=hGuQ8kuc9pgc9s8qqaq=dirpe0xb9q8qiLsFr0=vr0=vr0dc8meaabaqaciaacaGaaeqabaqabeGadaaakeaat0uy0HwzTfgDPnwy1egaryqtHrhAL1wy0L2yHvdaiqaacqWFlecsaaa@3762@ = {AAA, AAC, ACA, ACC, CCT}. Dashed colored links represent *δ *function for internal node (5) – in red, and for marked node (7) corresponding to the word AAC ∈ ℋ
 MathType@MTEF@5@5@+=feaafiart1ev1aaatCvAUfKttLearuWrP9MDH5MBPbIqV92AaeXatLxBI9gBaebbnrfifHhDYfgasaacH8akY=wiFfYdH8Gipec8Eeeu0xXdbba9frFj0=OqFfea0dXdd9vqai=hGuQ8kuc9pgc9s8qqaq=dirpe0xb9q8qiLsFr0=vr0=vr0dc8meaabaqaciaacaGaaeqabaqabeGadaaakeaat0uy0HwzTfgDPnwy1egaryqtHrhAL1wy0L2yHvdaiqaacqWFlecsaaa@3762@ – in purple.

**Example: **Let ℋ
 MathType@MTEF@5@5@+=feaafiart1ev1aaatCvAUfKttLearuWrP9MDH5MBPbIqV92AaeXatLxBI9gBaebbnrfifHhDYfgasaacH8akY=wiFfYdH8Gipec8Eeeu0xXdbba9frFj0=OqFfea0dXdd9vqai=hGuQ8kuc9pgc9s8qqaq=dirpe0xb9q8qiLsFr0=vr0=vr0dc8meaabaqaciaacaGaaeqabaqabeGadaaakeaat0uy0HwzTfgDPnwy1egaryqtHrhAL1wy0L2yHvdaiqaacqWFlecsaaa@3762@ be the set {AAA, AAC, ACA, ACA, CCT}. The corresponding tree T(ℋ)
 MathType@MTEF@5@5@+=feaafiart1ev1aaatCvAUfKttLearuWrP9MDH5MBPbIqV92AaeXatLxBI9gBaebbnrfifHhDYfgasaacH8akY=wiFfYdH8Gipec8Eeeu0xXdbba9frFj0=OqFfea0dXdd9vqai=hGuQ8kuc9pgc9s8qqaq=dirpe0xb9q8qiLsFr0=vr0=vr0dc8meaabaqaciaacaGaaeqabaqabeGadaaakeaat0uy0HwzTfgDPnwy1egaryqtHrhAL1wy0L2yHvdaiqaacqWFtepvcqGGOaakcqWFlecscqGGPaqkaaa@3AF1@ is depicted in Figure 1. Values of *δ *function are given in Table 1. Aho-Corasick traversal of tree T(ℋ)
 MathType@MTEF@5@5@+=feaafiart1ev1aaatCvAUfKttLearuWrP9MDH5MBPbIqV92AaeXatLxBI9gBaebbnrfifHhDYfgasaacH8akY=wiFfYdH8Gipec8Eeeu0xXdbba9frFj0=OqFfea0dXdd9vqai=hGuQ8kuc9pgc9s8qqaq=dirpe0xb9q8qiLsFr0=vr0=vr0dc8meaabaqaciaacaGaaeqabaqabeGadaaakeaat0uy0HwzTfgDPnwy1egaryqtHrhAL1wy0L2yHvdaiqaacqWFtepvcqGGOaakcqWFlecscqGGPaqkaaa@3AF1@ according to text *T *= 'ATGCCAACCTT' produces the following sequence of nodes {*q*_*i*_}_*i *≥ 1 _in Qℋ
 MathType@MTEF@5@5@+=feaafiart1ev1aaatCvAUfKttLearuWrP9MDH5MBPbIqV92AaeXatLxBI9gBaebbnrfifHhDYfgasaacH8akY=wiFfYdH8Gipec8Eeeu0xXdbba9frFj0=OqFfea0dXdd9vqai=hGuQ8kuc9pgc9s8qqaq=dirpe0xb9q8qiLsFr0=vr0=vr0dc8meaabaqaciaacaGaaeqabaqabeGadaaakeaacqWGrbqudaWgaaWcbaWenfgDOvwBHrxAJfwnHbqeg0uy0HwzTfgDPnwy1aaceaGae83cHGeabeaaaaa@38B9@ (the numbers of corresponding nodes in Figure 1 are shown in square brackets): A[1], *ε*[0], *ε*[0], C[2], CC[5], A[1], AA[3], AAC[7], ACC[9], CCT[10], *ε*[0].

**Table 1:** Values of *δ *function for the set ℋ
 MathType@MTEF@5@5@+=feaafiart1ev1aaatCvAUfKttLearuWrP9MDH5MBPbIqV92AaeXatLxBI9gBaebbnrfifHhDYfgasaacH8akY=wiFfYdH8Gipec8Eeeu0xXdbba9frFj0=OqFfea0dXdd9vqai=hGuQ8kuc9pgc9s8qqaq=dirpe0xb9q8qiLsFr0=vr0=vr0dc8meaabaqaciaacaGaaeqabaqabeGadaaakeaat0uy0HwzTfgDPnwy1egaryqtHrhAL1wy0L2yHvdaiqaacqWFlecsaaa@3762@ = {aaa, aac, aca, acc, cct}.

*q*\*α*	A	C	G	T
0	1	2	0	0
1	3	4	0	0
2	1	5	0	0
3	6	7	0	0
4	8	9	0	0
5	1	5	0	10
6	6	7	0	0
7	8	9	0	0
8	3	4	0	0
9	1	5	0	10
10	1	2	0	0

Values of *δ *(*q*, *α*) function for *q *∈ *Q *and *α *= *A*, *C*, *G*, *T *constructed for the set ℋ
 MathType@MTEF@5@5@+=feaafiart1ev1aaatCvAUfKttLearuWrP9MDH5MBPbIqV92AaeXatLxBI9gBaebbnrfifHhDYfgasaacH8akY=wiFfYdH8Gipec8Eeeu0xXdbba9frFj0=OqFfea0dXdd9vqai=hGuQ8kuc9pgc9s8qqaq=dirpe0xb9q8qiLsFr0=vr0=vr0dc8meaabaqaciaacaGaaeqabaqabeGadaaakeaat0uy0HwzTfgDPnwy1egaryqtHrhAL1wy0L2yHvdaiqaacqWFlecsaaa@3762@ = {AAA, AAC, ACA, ACC, CCT}.

T(ℋ)
 MathType@MTEF@5@5@+=feaafiart1ev1aaatCvAUfKttLearuWrP9MDH5MBPbIqV92AaeXatLxBI9gBaebbnrfifHhDYfgasaacH8akY=wiFfYdH8Gipec8Eeeu0xXdbba9frFj0=OqFfea0dXdd9vqai=hGuQ8kuc9pgc9s8qqaq=dirpe0xb9q8qiLsFr0=vr0=vr0dc8meaabaqaciaacaGaaeqabaqabeGadaaakeaat0uy0HwzTfgDPnwy1egaryqtHrhAL1wy0L2yHvdaiqaacqWFtepvcqGGOaakcqWFlecscqGGPaqkaaa@3AF1@ and transition function *δ *can be efficiently constructed with an algorithm proposed by Aho and Corasick [62]. Both time and space of the algorithm is proportional to the sum of lengths of all words from ℋ
 MathType@MTEF@5@5@+=feaafiart1ev1aaatCvAUfKttLearuWrP9MDH5MBPbIqV92AaeXatLxBI9gBaebbnrfifHhDYfgasaacH8akY=wiFfYdH8Gipec8Eeeu0xXdbba9frFj0=OqFfea0dXdd9vqai=hGuQ8kuc9pgc9s8qqaq=dirpe0xb9q8qiLsFr0=vr0=vr0dc8meaabaqaciaacaGaaeqabaqabeGadaaakeaat0uy0HwzTfgDPnwy1egaryqtHrhAL1wy0L2yHvdaiqaacqWFlecsaaa@3762@.

The combination of tree T(ℋ)
 MathType@MTEF@5@5@+=feaafiart1ev1aaatCvAUfKttLearuWrP9MDH5MBPbIqV92AaeXatLxBI9gBaebbnrfifHhDYfgasaacH8akY=wiFfYdH8Gipec8Eeeu0xXdbba9frFj0=OqFfea0dXdd9vqai=hGuQ8kuc9pgc9s8qqaq=dirpe0xb9q8qiLsFr0=vr0=vr0dc8meaabaqaciaacaGaaeqabaqabeGadaaakeaat0uy0HwzTfgDPnwy1egaryqtHrhAL1wy0L2yHvdaiqaacqWFtepvcqGGOaakcqWFlecscqGGPaqkaaa@3AF1@ and transition function *δ *allows solving numerous pattern matching problems: search of the first occurrence of a word from a given set, search of all occurrences, word counting, *etc*.

### Bernoulli text model. Probability to find at least one occurrence of a single motif

In this section we consider the simplest case. One computes the *p*-value for a single motif in a text *T*_*n *_of length *n*, assuming that *T*_*n *_is generated by independent Bernoulli random trials over alphabet Σ. The algorithm computes probabilities **P **(*L*_*n *_(ℋ
 MathType@MTEF@5@5@+=feaafiart1ev1aaatCvAUfKttLearuWrP9MDH5MBPbIqV92AaeXatLxBI9gBaebbnrfifHhDYfgasaacH8akY=wiFfYdH8Gipec8Eeeu0xXdbba9frFj0=OqFfea0dXdd9vqai=hGuQ8kuc9pgc9s8qqaq=dirpe0xb9q8qiLsFr0=vr0=vr0dc8meaabaqaciaacaGaaeqabaqabeGadaaakeaat0uy0HwzTfgDPnwy1egaryqtHrhAL1wy0L2yHvdaiqaacqWFlecsaaa@3762@; 1)) by induction on *n*.

To describe the algorithm we divide the set Σ^*i *^of all texts *T*_*i *_of length *i *into classes that do and do not contain occurrences of ℋ
 MathType@MTEF@5@5@+=feaafiart1ev1aaatCvAUfKttLearuWrP9MDH5MBPbIqV92AaeXatLxBI9gBaebbnrfifHhDYfgasaacH8akY=wiFfYdH8Gipec8Eeeu0xXdbba9frFj0=OqFfea0dXdd9vqai=hGuQ8kuc9pgc9s8qqaq=dirpe0xb9q8qiLsFr0=vr0=vr0dc8meaabaqaciaacaGaaeqabaqabeGadaaakeaat0uy0HwzTfgDPnwy1egaryqtHrhAL1wy0L2yHvdaiqaacqWFlecsaaa@3762@.

**Definition 1 ***A text T_*i *_belongs to class C*_*i *_(0; *q*) *iff*

*1. Length of T*_*i *_*is i*,

*2. T*_*i *_*does not contain words from *ℋ
 MathType@MTEF@5@5@+=feaafiart1ev1aaatCvAUfKttLearuWrP9MDH5MBPbIqV92AaeXatLxBI9gBaebbnrfifHhDYfgasaacH8akY=wiFfYdH8Gipec8Eeeu0xXdbba9frFj0=OqFfea0dXdd9vqai=hGuQ8kuc9pgc9s8qqaq=dirpe0xb9q8qiLsFr0=vr0=vr0dc8meaabaqaciaacaGaaeqabaqabeGadaaakeaat0uy0HwzTfgDPnwy1egaryqtHrhAL1wy0L2yHvdaiqaacqWFlecsaaa@3762@,

*3. A traversal AC *(T(ℋ)
 MathType@MTEF@5@5@+=feaafiart1ev1aaatCvAUfKttLearuWrP9MDH5MBPbIqV92AaeXatLxBI9gBaebbnrfifHhDYfgasaacH8akY=wiFfYdH8Gipec8Eeeu0xXdbba9frFj0=OqFfea0dXdd9vqai=hGuQ8kuc9pgc9s8qqaq=dirpe0xb9q8qiLsFr0=vr0=vr0dc8meaabaqaciaacaGaaeqabaqabeGadaaakeaat0uy0HwzTfgDPnwy1egaryqtHrhAL1wy0L2yHvdaiqaacqWFtepvcqGGOaakcqWFlecscqGGPaqkaaa@3AF1@, *T*_*i*_) *ends at node q*.

*A text T*_*i *_*belongs to class G*_*i *_(1) *iff*

*(i) Length of T*_*i *_*is i*,

*(ii) T*_*i *_*does contain at least one occurrence of a word from *ℋ
 MathType@MTEF@5@5@+=feaafiart1ev1aaatCvAUfKttLearuWrP9MDH5MBPbIqV92AaeXatLxBI9gBaebbnrfifHhDYfgasaacH8akY=wiFfYdH8Gipec8Eeeu0xXdbba9frFj0=OqFfea0dXdd9vqai=hGuQ8kuc9pgc9s8qqaq=dirpe0xb9q8qiLsFr0=vr0=vr0dc8meaabaqaciaacaGaaeqabaqabeGadaaakeaat0uy0HwzTfgDPnwy1egaryqtHrhAL1wy0L2yHvdaiqaacqWFlecsaaa@3762@.

For a given number *i *larger than *m*, the union for classes *C*_*i *_(0; *q*), where *q *is in Qℋ\ℋ
 MathType@MTEF@5@5@+=feaafiart1ev1aaatCvAUfKttLearuWrP9MDH5MBPbIqV92AaeXatLxBI9gBaebbnrfifHhDYfgasaacH8akY=wiFfYdH8Gipec8Eeeu0xXdbba9frFj0=OqFfea0dXdd9vqai=hGuQ8kuc9pgc9s8qqaq=dirpe0xb9q8qiLsFr0=vr0=vr0dc8meaabaqaciaacaGaaeqabaqabeGadaaakeaacqWGrbqudaWgaaWcbaWenfgDOvwBHrxAJfwnHbqeg0uy0HwzTfgDPnwy1aaceaGae83cHGeabeaakiabcYfaCjab=Tqiibaa@3AFC@ and the class *G*_*i *_(1) form a partition of the set Σ^*i *^of all texts of length *i*, i.e., any texts of length *i *belongs either to a class *C*_*i *_(0; *q*) for some *q *in Qℋ\ℋ
 MathType@MTEF@5@5@+=feaafiart1ev1aaatCvAUfKttLearuWrP9MDH5MBPbIqV92AaeXatLxBI9gBaebbnrfifHhDYfgasaacH8akY=wiFfYdH8Gipec8Eeeu0xXdbba9frFj0=OqFfea0dXdd9vqai=hGuQ8kuc9pgc9s8qqaq=dirpe0xb9q8qiLsFr0=vr0=vr0dc8meaabaqaciaacaGaaeqabaqabeGadaaakeaacqWGrbqudaWgaaWcbaWenfgDOvwBHrxAJfwnHbqeg0uy0HwzTfgDPnwy1aaceaGae83cHGeabeaakiabcYfaCjab=Tqiibaa@3AFC@, or to a class *G*_*i *_(1). Indeed, condition 3. means that the largest suffix of *T*_*i *_in Qℋ
 MathType@MTEF@5@5@+=feaafiart1ev1aaatCvAUfKttLearuWrP9MDH5MBPbIqV92AaeXatLxBI9gBaebbnrfifHhDYfgasaacH8akY=wiFfYdH8Gipec8Eeeu0xXdbba9frFj0=OqFfea0dXdd9vqai=hGuQ8kuc9pgc9s8qqaq=dirpe0xb9q8qiLsFr0=vr0=vr0dc8meaabaqaciaacaGaaeqabaqabeGadaaakeaacqWGrbqudaWgaaWcbaWenfgDOvwBHrxAJfwnHbqeg0uy0HwzTfgDPnwy1aaceaGae83cHGeabeaaaaa@38B9@ is *q*. It follows from condition 2. that classes *C*_*i *_(*q*; 0) are empty if *q *is in ℋ
 MathType@MTEF@5@5@+=feaafiart1ev1aaatCvAUfKttLearuWrP9MDH5MBPbIqV92AaeXatLxBI9gBaebbnrfifHhDYfgasaacH8akY=wiFfYdH8Gipec8Eeeu0xXdbba9frFj0=OqFfea0dXdd9vqai=hGuQ8kuc9pgc9s8qqaq=dirpe0xb9q8qiLsFr0=vr0=vr0dc8meaabaqaciaacaGaaeqabaqabeGadaaakeaat0uy0HwzTfgDPnwy1egaryqtHrhAL1wy0L2yHvdaiqaacqWFlecsaaa@3762@. A text *T*_*i *_of length *i *is in *G*_*i *_(1) if and only if a node of ℋ
 MathType@MTEF@5@5@+=feaafiart1ev1aaatCvAUfKttLearuWrP9MDH5MBPbIqV92AaeXatLxBI9gBaebbnrfifHhDYfgasaacH8akY=wiFfYdH8Gipec8Eeeu0xXdbba9frFj0=OqFfea0dXdd9vqai=hGuQ8kuc9pgc9s8qqaq=dirpe0xb9q8qiLsFr0=vr0=vr0dc8meaabaqaciaacaGaaeqabaqabeGadaaakeaat0uy0HwzTfgDPnwy1egaryqtHrhAL1wy0L2yHvdaiqaacqWFlecsaaa@3762@ was visited during the traversal.

Let **P **(*C*_*n *_(0; *q*)) and **P **(*G*_*n *_(1)) denote probabilities that a text *T*_*n *_belongs to class *C*_*n *_(0; *q*) and *G*_*n *_(1), respectively. Then, *L*_*n *_(ℋ
 MathType@MTEF@5@5@+=feaafiart1ev1aaatCvAUfKttLearuWrP9MDH5MBPbIqV92AaeXatLxBI9gBaebbnrfifHhDYfgasaacH8akY=wiFfYdH8Gipec8Eeeu0xXdbba9frFj0=OqFfea0dXdd9vqai=hGuQ8kuc9pgc9s8qqaq=dirpe0xb9q8qiLsFr0=vr0=vr0dc8meaabaqaciaacaGaaeqabaqabeGadaaakeaat0uy0HwzTfgDPnwy1egaryqtHrhAL1wy0L2yHvdaiqaacqWFlecsaaa@3762@; 1) = *G*_*n *_(1); therefore the desired *p*-value **P **(*L*_*n *_(ℋ
 MathType@MTEF@5@5@+=feaafiart1ev1aaatCvAUfKttLearuWrP9MDH5MBPbIqV92AaeXatLxBI9gBaebbnrfifHhDYfgasaacH8akY=wiFfYdH8Gipec8Eeeu0xXdbba9frFj0=OqFfea0dXdd9vqai=hGuQ8kuc9pgc9s8qqaq=dirpe0xb9q8qiLsFr0=vr0=vr0dc8meaabaqaciaacaGaaeqabaqabeGadaaakeaat0uy0HwzTfgDPnwy1egaryqtHrhAL1wy0L2yHvdaiqaacqWFlecsaaa@3762@; 1)) is equal to **P **(*G*_*n *_(1)).

The algorithm calculates probabilities **P **(*C*_*i *_(0; *q*)) and **P **(*G*_*i *_(1)) using induction on length *i*. For *i *= 0, these probabilities obviously comply with: **P **(*C*_0 _(0; *ε*)) = 1; **P **(*C*_0 _(0; *q*)) = 0, for any *q *≠ *ε*; **P **(*G*_0 _(1)) = 0.

The values of **P **(*C*_*i*+1 _(0; *q*)) and **P **(*G*_*i*+1 _(1)) are calculated using values of **P **(*C*_*i *_(0; *q*)) and **P **(*G*_*i *_(1)). Therefore, the needed space is proportional to the size of Qℋ
 MathType@MTEF@5@5@+=feaafiart1ev1aaatCvAUfKttLearuWrP9MDH5MBPbIqV92AaeXatLxBI9gBaebbnrfifHhDYfgasaacH8akY=wiFfYdH8Gipec8Eeeu0xXdbba9frFj0=OqFfea0dXdd9vqai=hGuQ8kuc9pgc9s8qqaq=dirpe0xb9q8qiLsFr0=vr0=vr0dc8meaabaqaciaacaGaaeqabaqabeGadaaakeaacqWGrbqudaWgaaWcbaWenfgDOvwBHrxAJfwnHbqeg0uy0HwzTfgDPnwy1aaceaGae83cHGeabeaaaaa@38B9@ (see section *Extensions and complexity *below).

Calculation of values **P **(*C*_*i*+1 _(0; *q*)) and **P **(*G*_*i*+1 _(1)) is based on the following observations. Let *U *be a set of texts of the same length over the alphabet Σ, **P **(*U*) the probability of *U *in the Bernoulli model and *a *a character in Σ. Let *U*·*a *be the set of all possible concatenations, i.e., *U*·*a *= {*xa*|*x *∈ *U*}. And in the case of the Bernoulli model

**P **(*U*·*a*) = **P **(*U*) **P **(*a*).

Then the following relations hold for any *i *∈ {1, ..., *n *- 1} and Σ:

(i) if the text *T*_*i *_contains a word from ℋ
 MathType@MTEF@5@5@+=feaafiart1ev1aaatCvAUfKttLearuWrP9MDH5MBPbIqV92AaeXatLxBI9gBaebbnrfifHhDYfgasaacH8akY=wiFfYdH8Gipec8Eeeu0xXdbba9frFj0=OqFfea0dXdd9vqai=hGuQ8kuc9pgc9s8qqaq=dirpe0xb9q8qiLsFr0=vr0=vr0dc8meaabaqaciaacaGaaeqabaqabeGadaaakeaat0uy0HwzTfgDPnwy1egaryqtHrhAL1wy0L2yHvdaiqaacqWFlecsaaa@3762@ then all its concatenations with characters from Σ would contain a word from ℋ
 MathType@MTEF@5@5@+=feaafiart1ev1aaatCvAUfKttLearuWrP9MDH5MBPbIqV92AaeXatLxBI9gBaebbnrfifHhDYfgasaacH8akY=wiFfYdH8Gipec8Eeeu0xXdbba9frFj0=OqFfea0dXdd9vqai=hGuQ8kuc9pgc9s8qqaq=dirpe0xb9q8qiLsFr0=vr0=vr0dc8meaabaqaciaacaGaaeqabaqabeGadaaakeaat0uy0HwzTfgDPnwy1egaryqtHrhAL1wy0L2yHvdaiqaacqWFlecsaaa@3762@; i.e.,

*G*_*i *_(1)·*a *⊂ *G*_*i*+1 _(1).

(ii) if the text *T*_*i *_does not contain a word from ℋ
 MathType@MTEF@5@5@+=feaafiart1ev1aaatCvAUfKttLearuWrP9MDH5MBPbIqV92AaeXatLxBI9gBaebbnrfifHhDYfgasaacH8akY=wiFfYdH8Gipec8Eeeu0xXdbba9frFj0=OqFfea0dXdd9vqai=hGuQ8kuc9pgc9s8qqaq=dirpe0xb9q8qiLsFr0=vr0=vr0dc8meaabaqaciaacaGaaeqabaqabeGadaaakeaat0uy0HwzTfgDPnwy1egaryqtHrhAL1wy0L2yHvdaiqaacqWFlecsaaa@3762@ and belongs to *C*_*i*+1 _(0; *q*), i.e., ends with *q *∈ Qℋ\ℋ
 MathType@MTEF@5@5@+=feaafiart1ev1aaatCvAUfKttLearuWrP9MDH5MBPbIqV92AaeXatLxBI9gBaebbnrfifHhDYfgasaacH8akY=wiFfYdH8Gipec8Eeeu0xXdbba9frFj0=OqFfea0dXdd9vqai=hGuQ8kuc9pgc9s8qqaq=dirpe0xb9q8qiLsFr0=vr0=vr0dc8meaabaqaciaacaGaaeqabaqabeGadaaakeaacqWGrbqudaWgaaWcbaWenfgDOvwBHrxAJfwnHbqeg0uy0HwzTfgDPnwy1aaceaGae83cHGeabeaakiabcYfaCjab=Tqiibaa@3AFC@, then its concatenation *T*_*i*_·*a *belongs to the class determined by the result of the Aho-Corasick transition function *δ *(*q, a*); i.e.,

if *δ *(*q*, *a*) ∈ ℋ
 MathType@MTEF@5@5@+=feaafiart1ev1aaatCvAUfKttLearuWrP9MDH5MBPbIqV92AaeXatLxBI9gBaebbnrfifHhDYfgasaacH8akY=wiFfYdH8Gipec8Eeeu0xXdbba9frFj0=OqFfea0dXdd9vqai=hGuQ8kuc9pgc9s8qqaq=dirpe0xb9q8qiLsFr0=vr0=vr0dc8meaabaqaciaacaGaaeqabaqabeGadaaakeaat0uy0HwzTfgDPnwy1egaryqtHrhAL1wy0L2yHvdaiqaacqWFlecsaaa@3762@,   then *C*_*i *_(0; *q*)·*a *⊂ *C*_*i*+1 _(0; *δ *(*q, a*))

otherwise   *C*_*i *_(0; *q*) ⊂ *G*_*i*+1 _(1).

Remembering that classes *C*_*i *_(0; *q*) for different *q *and *G*_*i *_(1) form a partition of Σ^*i*^, we obtain the following relation for the texts containing words from ℋ
 MathType@MTEF@5@5@+=feaafiart1ev1aaatCvAUfKttLearuWrP9MDH5MBPbIqV92AaeXatLxBI9gBaebbnrfifHhDYfgasaacH8akY=wiFfYdH8Gipec8Eeeu0xXdbba9frFj0=OqFfea0dXdd9vqai=hGuQ8kuc9pgc9s8qqaq=dirpe0xb9q8qiLsFr0=vr0=vr0dc8meaabaqaciaacaGaaeqabaqabeGadaaakeaat0uy0HwzTfgDPnwy1egaryqtHrhAL1wy0L2yHvdaiqaacqWFlecsaaa@3762@:

Gi+1(1)={∪a∈ΣGi(1)⋅a}∪{∪(q,a);δ(q,a)∈ℋCi(0;q)⋅a}.
 MathType@MTEF@5@5@+=feaafiart1ev1aaatCvAUfKttLearuWrP9MDH5MBPbIqV92AaeXatLxBI9gBaebbnrfifHhDYfgasaacH8akY=wiFfYdH8Gipec8Eeeu0xXdbba9frFj0=OqFfea0dXdd9vqai=hGuQ8kuc9pgc9s8qqaq=dirpe0xb9q8qiLsFr0=vr0=vr0dc8meaabaqaciaacaGaaeqabaqabeGadaaakeaacqWGhbWrdaWgaaWcbaGaemyAaKMaey4kaSIaeGymaedabeaakiabcIcaOiabigdaXiabcMcaPiabg2da9iabcUha7naatafabaGaem4raC0aaSbaaSqaaiabdMgaPbqabaGccqGGOaakcqaIXaqmcqGGPaqkcqGHflY1cqWGHbqycqGG9bqFcqGHQicYcqGG7bWEdaWeqbqaaiabdoeadnaaBaaaleaacqWGPbqAaeqaaOGaeiikaGIaeGimaaJaei4oaSJaemyCaeNaeiykaKIaeyyXICTaemyyaeMaeiyFa0haleaacqGGOaakcqWGXbqCcqGGSaalcqWGHbqycqGGPaqkcqGG7aWoiiGacqWF0oazcqGGOaakcqWGXbqCcqGGSaalcqWGHbqycqGGPaqkcqGHiiIZt0uy0HwzTfgDPnwy1egaryqtHrhAL1wy0L2yHvdaiqaacqGFlecsaeqaniablQIivbGccqGGUaGlaSqaaiabdggaHjabgIGiolabfo6atbqab0GaeSOkIufaaaa@72A7@

Similarly, classes of texts that do not contain words from ℋ
 MathType@MTEF@5@5@+=feaafiart1ev1aaatCvAUfKttLearuWrP9MDH5MBPbIqV92AaeXatLxBI9gBaebbnrfifHhDYfgasaacH8akY=wiFfYdH8Gipec8Eeeu0xXdbba9frFj0=OqFfea0dXdd9vqai=hGuQ8kuc9pgc9s8qqaq=dirpe0xb9q8qiLsFr0=vr0=vr0dc8meaabaqaciaacaGaaeqabaqabeGadaaakeaat0uy0HwzTfgDPnwy1egaryqtHrhAL1wy0L2yHvdaiqaacqWFlecsaaa@3762@ satisfy

∀q′∈Qℋ\ℋ:Ci+1(0;q′)=∪(q,a);δ(q,a)=q′Ci(0;q)⋅a.
 MathType@MTEF@5@5@+=feaafiart1ev1aaatCvAUfKttLearuWrP9MDH5MBPbIqV92AaeXatLxBI9gBaebbnrfifHhDYfgasaacH8akY=wiFfYdH8Gipec8Eeeu0xXdbba9frFj0=OqFfea0dXdd9vqai=hGuQ8kuc9pgc9s8qqaq=dirpe0xb9q8qiLsFr0=vr0=vr0dc8meaabaqaciaacaGaaeqabaqabeGadaaakeaafaqabeqacaaabaGaeyiaIiIafmyCaeNbauaacqGHiiIZcqWGrbqudaWgaaWcbaWenfgDOvwBHrxAJfwnHbqeg0uy0HwzTfgDPnwy1aaceaGae83cHGeabeaakiabcYfaCjab=TqiijabcQda6aqaaiabdoeadnaaBaaaleaacqWGPbqAcqGHRaWkcqaIXaqmaeqaaOGaeiikaGIaeGimaaJaei4oaSJafmyCaeNbauaacqGGPaqkcqGH9aqpdaWeqbqaaiabdoeadnaaBaaaleaacqWGPbqAaeqaaOGaeiikaGIaeGimaaJaei4oaSJaemyCaeNaeiykaKIaeyyXICTaemyyaegaleaacqGGOaakcqWGXbqCcqGGSaalcqWGHbqycqGGPaqkcqGG7aWoiiGacqGF0oazcqGGOaakcqWGXbqCcqGGSaalcqWGHbqycqGGPaqkcqGH9aqpcuWGXbqCgaqbaaqab0GaeSOkIufakiabc6caUaaaaaa@67F3@

Classes *C*_*i *_(0; *q*) for different *q *in Qℋ\ℋ
 MathType@MTEF@5@5@+=feaafiart1ev1aaatCvAUfKttLearuWrP9MDH5MBPbIqV92AaeXatLxBI9gBaebbnrfifHhDYfgasaacH8akY=wiFfYdH8Gipec8Eeeu0xXdbba9frFj0=OqFfea0dXdd9vqai=hGuQ8kuc9pgc9s8qqaq=dirpe0xb9q8qiLsFr0=vr0=vr0dc8meaabaqaciaacaGaaeqabaqabeGadaaakeaacqWGrbqudaWgaaWcbaWenfgDOvwBHrxAJfwnHbqeg0uy0HwzTfgDPnwy1aaceaGae83cHGeabeaakiabcYfaCjab=Tqiibaa@3AFC@ and *G*_*i *_(1) form a partition of Σ^*i*^; classes *C*_*i *_(0; *q*) are empty if *q *is in ℋ
 MathType@MTEF@5@5@+=feaafiart1ev1aaatCvAUfKttLearuWrP9MDH5MBPbIqV92AaeXatLxBI9gBaebbnrfifHhDYfgasaacH8akY=wiFfYdH8Gipec8Eeeu0xXdbba9frFj0=OqFfea0dXdd9vqai=hGuQ8kuc9pgc9s8qqaq=dirpe0xb9q8qiLsFr0=vr0=vr0dc8meaabaqaciaacaGaaeqabaqabeGadaaakeaat0uy0HwzTfgDPnwy1egaryqtHrhAL1wy0L2yHvdaiqaacqWFlecsaaa@3762@. Relations (5) and (6) with the help of (1) yield the recursive expressions for probabilities **P **(*C*_*i+i *_(0; *q*)) and **P **(*G*_*i*+1 _(1)) in the Bernoulli case:

P(Gi+1(1))=P(Gi(1))+∑(q,a):δ(q,a)∈ℋP(Ci(0;q))⋅p(a),
 MathType@MTEF@5@5@+=feaafiart1ev1aaatCvAUfKttLearuWrP9MDH5MBPbIqV92AaeXatLxBI9gBaebbnrfifHhDYfgasaacH8akY=wiFfYdH8Gipec8Eeeu0xXdbba9frFj0=OqFfea0dXdd9vqai=hGuQ8kuc9pgc9s8qqaq=dirpe0xb9q8qiLsFr0=vr0=vr0dc8meaabaqaciaacaGaaeqabaqabeGadaaakeaaieqacqWFqbaucqGGOaakcqWGhbWrdaWgaaWcbaGaemyAaKMaey4kaSIaeGymaedabeaakiabcIcaOiabigdaXiabcMcaPiabcMcaPiabg2da9iab=bfaqjabcIcaOiabdEeahnaaBaaaleaacqWGPbqAaeqaaOGaeiikaGIaeGymaeJaeiykaKIaeiykaKIaey4kaSYaaabuaeaacqWFqbaucqGGOaakcqWGdbWqdaWgaaWcbaGaemyAaKgabeaakiabcIcaOiabicdaWiabcUda7iabdghaXjabcMcaPiabcMcaPiabgwSixlabdchaWjabcIcaOiabdggaHjabcMcaPaWcbaGaeiikaGIaemyCaeNaeiilaWIaemyyaeMaeiykaKIaeiOoaOdcciGae4hTdqMaeiikaGIaemyCaeNaeiilaWIaemyyaeMaeiykaKIaeyicI48enfgDOvwBHrxAJfwnHbqeg0uy0HwzTfgDPnwy1aaceaGae03cHGeabeqdcqGHris5aOGaeiilaWcaaa@6E5E@

P(Ci+1(0;q′))=∑(q,a):δ(q,a)=q′P(Ci(0;q))⋅p(a).
 MathType@MTEF@5@5@+=feaafiart1ev1aaatCvAUfKttLearuWrP9MDH5MBPbIqV92AaeXatLxBI9gBaebbnrfifHhDYfgasaacH8akY=wiFfYdH8Gipec8Eeeu0xXdbba9frFj0=OqFfea0dXdd9vqai=hGuQ8kuc9pgc9s8qqaq=dirpe0xb9q8qiLsFr0=vr0=vr0dc8meaabaqaciaacaGaaeqabaqabeGadaaakeaaieqacqWFqbaucqGGOaakcqWGdbWqdaWgaaWcbaGaemyAaKMaey4kaSIaeGymaedabeaakiabcIcaOiabicdaWiabcUda7iqbdghaXzaafaGaeiykaKIaeiykaKIaeyypa0ZaaabuaeaacqWFqbaucqGGOaakcqWGdbWqdaWgaaWcbaGaemyAaKgabeaakiabcIcaOiabicdaWiabcUda7iabdghaXjabcMcaPiabcMcaPiabgwSixlabdchaWjabcIcaOiabdggaHjabcMcaPaWcbaGaeiikaGIaemyCaeNaeiilaWIaemyyaeMaeiykaKIaeiOoaOdcciGae4hTdqMaeiikaGIaemyCaeNaeiilaWIaemyyaeMaeiykaKIaeyypa0JafmyCaeNbauaaaeqaniabggHiLdGccqGGUaGlaaa@5E0F@

The run-time for each step of the computation of *C*_*i*+1 _(0; *q*) and *G*_*i*+1 _(1) is *O *(|Qℋ
 MathType@MTEF@5@5@+=feaafiart1ev1aaatCvAUfKttLearuWrP9MDH5MBPbIqV92AaeXatLxBI9gBaebbnrfifHhDYfgasaacH8akY=wiFfYdH8Gipec8Eeeu0xXdbba9frFj0=OqFfea0dXdd9vqai=hGuQ8kuc9pgc9s8qqaq=dirpe0xb9q8qiLsFr0=vr0=vr0dc8meaabaqaciaacaGaaeqabaqabeGadaaakeaacqWGrbqudaWgaaWcbaWenfgDOvwBHrxAJfwnHbqeg0uy0HwzTfgDPnwy1aaceaGae83cHGeabeaaaaa@38B9@|·|Σ|); therefore the total time of all *n *stages of *p*-value computation is *O *(|Qℋ
 MathType@MTEF@5@5@+=feaafiart1ev1aaatCvAUfKttLearuWrP9MDH5MBPbIqV92AaeXatLxBI9gBaebbnrfifHhDYfgasaacH8akY=wiFfYdH8Gipec8Eeeu0xXdbba9frFj0=OqFfea0dXdd9vqai=hGuQ8kuc9pgc9s8qqaq=dirpe0xb9q8qiLsFr0=vr0=vr0dc8meaabaqaciaacaGaaeqabaqabeGadaaakeaacqWGrbqudaWgaaWcbaWenfgDOvwBHrxAJfwnHbqeg0uy0HwzTfgDPnwy1aaceaGae83cHGeabeaaaaa@38B9@|·|Σ|·*n*).

The approach described in this section can be readily extended to the case of multiple occurrences of motif ℋ
 MathType@MTEF@5@5@+=feaafiart1ev1aaatCvAUfKttLearuWrP9MDH5MBPbIqV92AaeXatLxBI9gBaebbnrfifHhDYfgasaacH8akY=wiFfYdH8Gipec8Eeeu0xXdbba9frFj0=OqFfea0dXdd9vqai=hGuQ8kuc9pgc9s8qqaq=dirpe0xb9q8qiLsFr0=vr0=vr0dc8meaabaqaciaacaGaaeqabaqabeGadaaakeaat0uy0HwzTfgDPnwy1egaryqtHrhAL1wy0L2yHvdaiqaacqWFlecsaaa@3762@. The detailed procedure can be found in Additional file 1.

### Bernoulli text model. Probability to find multiple occurrences of multiple motifs

DNA transcription is usually regulated with several factors simultaneously interacting with DNA and specifically recognizing different DNA sites. Individual regulatory segment of DNA can contain many binding sites for several factors, often substantially overlapping with each other [5]. This brings about a problem of studying of co-occurring motifs.

Let (ℋ1,...,ℋs
 MathType@MTEF@5@5@+=feaafiart1ev1aaatCvAUfKttLearuWrP9MDH5MBPbIqV92AaeXatLxBI9gBaebbnrfifHhDYfgasaacH8akY=wiFfYdH8Gipec8Eeeu0xXdbba9frFj0=OqFfea0dXdd9vqai=hGuQ8kuc9pgc9s8qqaq=dirpe0xb9q8qiLsFr0=vr0=vr0dc8meaabaqaciaacaGaaeqabaqabeGadaaakeaat0uy0HwzTfgDPnwy1egaryqtHrhAL1wy0L2yHvdaiqaacqWFlecsdaWgaaWcbaGaeGymaedabeaakiabcYcaSiabc6caUiabc6caUiabc6caUiabcYcaSiab=TqiinaaBaaaleaacqWGZbWCaeqaaaaa@3F88@) be *s *different motifs. Our objective is to calculate the probability that motifs (ℋ1,...,ℋs
 MathType@MTEF@5@5@+=feaafiart1ev1aaatCvAUfKttLearuWrP9MDH5MBPbIqV92AaeXatLxBI9gBaebbnrfifHhDYfgasaacH8akY=wiFfYdH8Gipec8Eeeu0xXdbba9frFj0=OqFfea0dXdd9vqai=hGuQ8kuc9pgc9s8qqaq=dirpe0xb9q8qiLsFr0=vr0=vr0dc8meaabaqaciaacaGaaeqabaqabeGadaaakeaat0uy0HwzTfgDPnwy1egaryqtHrhAL1wy0L2yHvdaiqaacqWFlecsdaWgaaWcbaGaeGymaedabeaakiabcYcaSiabc6caUiabc6caUiabc6caUiabcYcaSiab=TqiinaaBaaaleaacqWGZbWCaeqaaaaa@3F88@) have respectively at least (*k*_1_, ..., *k*_*s*_) possibly overlapping occurrences in the random text *T*_*n *_of the length *n*. This *p*-value is the probability **P **(*L*_*n *_(ℋ1,...,ℋs
 MathType@MTEF@5@5@+=feaafiart1ev1aaatCvAUfKttLearuWrP9MDH5MBPbIqV92AaeXatLxBI9gBaebbnrfifHhDYfgasaacH8akY=wiFfYdH8Gipec8Eeeu0xXdbba9frFj0=OqFfea0dXdd9vqai=hGuQ8kuc9pgc9s8qqaq=dirpe0xb9q8qiLsFr0=vr0=vr0dc8meaabaqaciaacaGaaeqabaqabeGadaaakeaat0uy0HwzTfgDPnwy1egaryqtHrhAL1wy0L2yHvdaiqaacqWFlecsdaWgaaWcbaGaeGymaedabeaakiabcYcaSiabc6caUiabc6caUiabc6caUiabcYcaSiab=TqiinaaBaaaleaacqWGZbWCaeqaaaaa@3F88@; *k*_1_, ..., *k*_*s*_)) to obtain text *T*_*n *_belonging to the set of texts *L*_*n *_(ℋ1,...,ℋs
 MathType@MTEF@5@5@+=feaafiart1ev1aaatCvAUfKttLearuWrP9MDH5MBPbIqV92AaeXatLxBI9gBaebbnrfifHhDYfgasaacH8akY=wiFfYdH8Gipec8Eeeu0xXdbba9frFj0=OqFfea0dXdd9vqai=hGuQ8kuc9pgc9s8qqaq=dirpe0xb9q8qiLsFr0=vr0=vr0dc8meaabaqaciaacaGaaeqabaqabeGadaaakeaat0uy0HwzTfgDPnwy1egaryqtHrhAL1wy0L2yHvdaiqaacqWFlecsdaWgaaWcbaGaeGymaedabeaakiabcYcaSiabc6caUiabc6caUiabc6caUiabcYcaSiab=TqiinaaBaaaleaacqWGZbWCaeqaaaaa@3F88@; *k*_1_, ..., *k*_*s*_). In this section, we will suppose that the probability of each text is given by Bernoulli model. The Markov case will be considered in the next subsection. The recursion for multiple occurrences of multiple motifs obtained here is rather tricky. Therefore we suggest the reader to see Additional file 1 where we describe the recursion for the simpler case of multiple occurrences of a single motif

Let us consider the union ℋ
 MathType@MTEF@5@5@+=feaafiart1ev1aaatCvAUfKttLearuWrP9MDH5MBPbIqV92AaeXatLxBI9gBaebbnrfifHhDYfgasaacH8akY=wiFfYdH8Gipec8Eeeu0xXdbba9frFj0=OqFfea0dXdd9vqai=hGuQ8kuc9pgc9s8qqaq=dirpe0xb9q8qiLsFr0=vr0=vr0dc8meaabaqaciaacaGaaeqabaqabeGadaaakeaat0uy0HwzTfgDPnwy1egaryqtHrhAL1wy0L2yHvdaiqaacqWFlecsaaa@3762@ of individual motifs ℋ=ℋ1∪⋯∪ℋs
 MathType@MTEF@5@5@+=feaafiart1ev1aaatCvAUfKttLearuWrP9MDH5MBPbIqV92AaeXatLxBI9gBaebbnrfifHhDYfgasaacH8akY=wiFfYdH8Gipec8Eeeu0xXdbba9frFj0=OqFfea0dXdd9vqai=hGuQ8kuc9pgc9s8qqaq=dirpe0xb9q8qiLsFr0=vr0=vr0dc8meaabaqaciaacaGaaeqabaqabeGadaaakeaat0uy0HwzTfgDPnwy1egaryqtHrhAL1wy0L2yHvdaiqaacqWFlecscqGH9aqpcqWFlecsdaWgaaWcbaGaeGymaedabeaakiabgQIiilabl+UimjabgQIiilab=TqiinaaBaaaleaacqWGZbWCaeqaaaaa@4249@. It contains all words that belong to any of motifs ℋ
 MathType@MTEF@5@5@+=feaafiart1ev1aaatCvAUfKttLearuWrP9MDH5MBPbIqV92AaeXatLxBI9gBaebbnrfifHhDYfgasaacH8akY=wiFfYdH8Gipec8Eeeu0xXdbba9frFj0=OqFfea0dXdd9vqai=hGuQ8kuc9pgc9s8qqaq=dirpe0xb9q8qiLsFr0=vr0=vr0dc8meaabaqaciaacaGaaeqabaqabeGadaaakeaat0uy0HwzTfgDPnwy1egaryqtHrhAL1wy0L2yHvdaiqaacqWFlecsaaa@3762@_*i*_. The tree T(ℋ)
 MathType@MTEF@5@5@+=feaafiart1ev1aaatCvAUfKttLearuWrP9MDH5MBPbIqV92AaeXatLxBI9gBaebbnrfifHhDYfgasaacH8akY=wiFfYdH8Gipec8Eeeu0xXdbba9frFj0=OqFfea0dXdd9vqai=hGuQ8kuc9pgc9s8qqaq=dirpe0xb9q8qiLsFr0=vr0=vr0dc8meaabaqaciaacaGaaeqabaqabeGadaaakeaat0uy0HwzTfgDPnwy1egaryqtHrhAL1wy0L2yHvdaiqaacqWFtepvcqGGOaakcqWFlecscqGGPaqkaaa@3AF1@ is constructed for the overall set ℋ
 MathType@MTEF@5@5@+=feaafiart1ev1aaatCvAUfKttLearuWrP9MDH5MBPbIqV92AaeXatLxBI9gBaebbnrfifHhDYfgasaacH8akY=wiFfYdH8Gipec8Eeeu0xXdbba9frFj0=OqFfea0dXdd9vqai=hGuQ8kuc9pgc9s8qqaq=dirpe0xb9q8qiLsFr0=vr0=vr0dc8meaabaqaciaacaGaaeqabaqabeGadaaakeaat0uy0HwzTfgDPnwy1egaryqtHrhAL1wy0L2yHvdaiqaacqWFlecsaaa@3762@, its nodes Qℋ
 MathType@MTEF@5@5@+=feaafiart1ev1aaatCvAUfKttLearuWrP9MDH5MBPbIqV92AaeXatLxBI9gBaebbnrfifHhDYfgasaacH8akY=wiFfYdH8Gipec8Eeeu0xXdbba9frFj0=OqFfea0dXdd9vqai=hGuQ8kuc9pgc9s8qqaq=dirpe0xb9q8qiLsFr0=vr0=vr0dc8meaabaqaciaacaGaaeqabaqabeGadaaakeaacqWGrbqudaWgaaWcbaWenfgDOvwBHrxAJfwnHbqeg0uy0HwzTfgDPnwy1aaceaGae83cHGeabeaaaaa@38B9@ contain all possible prefixes of all motifs from (ℋ1,...,ℋs
 MathType@MTEF@5@5@+=feaafiart1ev1aaatCvAUfKttLearuWrP9MDH5MBPbIqV92AaeXatLxBI9gBaebbnrfifHhDYfgasaacH8akY=wiFfYdH8Gipec8Eeeu0xXdbba9frFj0=OqFfea0dXdd9vqai=hGuQ8kuc9pgc9s8qqaq=dirpe0xb9q8qiLsFr0=vr0=vr0dc8meaabaqaciaacaGaaeqabaqabeGadaaakeaat0uy0HwzTfgDPnwy1egaryqtHrhAL1wy0L2yHvdaiqaacqWFlecsdaWgaaWcbaGaeGymaedabeaakiabcYcaSiabc6caUiabc6caUiabc6caUiabcYcaSiab=TqiinaaBaaaleaacqWGZbWCaeqaaaaa@3F88@). A node of the tree *q *∈ Qℋ
 MathType@MTEF@5@5@+=feaafiart1ev1aaatCvAUfKttLearuWrP9MDH5MBPbIqV92AaeXatLxBI9gBaebbnrfifHhDYfgasaacH8akY=wiFfYdH8Gipec8Eeeu0xXdbba9frFj0=OqFfea0dXdd9vqai=hGuQ8kuc9pgc9s8qqaq=dirpe0xb9q8qiLsFr0=vr0=vr0dc8meaabaqaciaacaGaaeqabaqabeGadaaakeaacqWGrbqudaWgaaWcbaWenfgDOvwBHrxAJfwnHbqeg0uy0HwzTfgDPnwy1aaceaGae83cHGeabeaaaaa@38B9@ can belong to some motif ℋ
 MathType@MTEF@5@5@+=feaafiart1ev1aaatCvAUfKttLearuWrP9MDH5MBPbIqV92AaeXatLxBI9gBaebbnrfifHhDYfgasaacH8akY=wiFfYdH8Gipec8Eeeu0xXdbba9frFj0=OqFfea0dXdd9vqai=hGuQ8kuc9pgc9s8qqaq=dirpe0xb9q8qiLsFr0=vr0=vr0dc8meaabaqaciaacaGaaeqabaqabeGadaaakeaat0uy0HwzTfgDPnwy1egaryqtHrhAL1wy0L2yHvdaiqaacqWFlecsaaa@3762@_*k *_or simultaneously to several different motifs from {ℋ
 MathType@MTEF@5@5@+=feaafiart1ev1aaatCvAUfKttLearuWrP9MDH5MBPbIqV92AaeXatLxBI9gBaebbnrfifHhDYfgasaacH8akY=wiFfYdH8Gipec8Eeeu0xXdbba9frFj0=OqFfea0dXdd9vqai=hGuQ8kuc9pgc9s8qqaq=dirpe0xb9q8qiLsFr0=vr0=vr0dc8meaabaqaciaacaGaaeqabaqabeGadaaakeaat0uy0HwzTfgDPnwy1egaryqtHrhAL1wy0L2yHvdaiqaacqWFlecsaaa@3762@_*j*_}_1≤*j*≤*s*_. Let each node *q *∈ Qℋ
 MathType@MTEF@5@5@+=feaafiart1ev1aaatCvAUfKttLearuWrP9MDH5MBPbIqV92AaeXatLxBI9gBaebbnrfifHhDYfgasaacH8akY=wiFfYdH8Gipec8Eeeu0xXdbba9frFj0=OqFfea0dXdd9vqai=hGuQ8kuc9pgc9s8qqaq=dirpe0xb9q8qiLsFr0=vr0=vr0dc8meaabaqaciaacaGaaeqabaqabeGadaaakeaacqWGrbqudaWgaaWcbaWenfgDOvwBHrxAJfwnHbqeg0uy0HwzTfgDPnwy1aaceaGae83cHGeabeaaaaa@38B9@ be marked with numbers *j *of motifs ℋ
 MathType@MTEF@5@5@+=feaafiart1ev1aaatCvAUfKttLearuWrP9MDH5MBPbIqV92AaeXatLxBI9gBaebbnrfifHhDYfgasaacH8akY=wiFfYdH8Gipec8Eeeu0xXdbba9frFj0=OqFfea0dXdd9vqai=hGuQ8kuc9pgc9s8qqaq=dirpe0xb9q8qiLsFr0=vr0=vr0dc8meaabaqaciaacaGaaeqabaqabeGadaaakeaat0uy0HwzTfgDPnwy1egaryqtHrhAL1wy0L2yHvdaiqaacqWFlecsaaa@3762@_*j *_to which it belongs. Nodes, corresponding to proper prefixes of ℋ
 MathType@MTEF@5@5@+=feaafiart1ev1aaatCvAUfKttLearuWrP9MDH5MBPbIqV92AaeXatLxBI9gBaebbnrfifHhDYfgasaacH8akY=wiFfYdH8Gipec8Eeeu0xXdbba9frFj0=OqFfea0dXdd9vqai=hGuQ8kuc9pgc9s8qqaq=dirpe0xb9q8qiLsFr0=vr0=vr0dc8meaabaqaciaacaGaaeqabaqabeGadaaakeaat0uy0HwzTfgDPnwy1egaryqtHrhAL1wy0L2yHvdaiqaacqWFlecsaaa@3762@, remain unmarked. The transition function δ:Qℋ×Σ→Qℋ
 MathType@MTEF@5@5@+=feaafiart1ev1aaatCvAUfKttLearuWrP9MDH5MBPbIqV92AaeXatLxBI9gBaebbnrfifHhDYfgasaacH8akY=wiFfYdH8Gipec8Eeeu0xXdbba9frFj0=OqFfea0dXdd9vqai=hGuQ8kuc9pgc9s8qqaq=dirpe0xb9q8qiLsFr0=vr0=vr0dc8meaabaqaciaacaGaaeqabaqabeGadaaakeaaiiGacqWF0oazcqGG6aGocqWGrbqudaWgaaWcbaWenfgDOvwBHrxAJfwnHbqeg0uy0HwzTfgDPnwy1aaceaGae43cHGeabeaakiabgEna0kabfo6atjabgkziUkabdgfarnaaBaaaleaacqGFlecsaeqaaaaa@4341@ is defined as it was defined in the case of a single motif for the unified motif ℋ
 MathType@MTEF@5@5@+=feaafiart1ev1aaatCvAUfKttLearuWrP9MDH5MBPbIqV92AaeXatLxBI9gBaebbnrfifHhDYfgasaacH8akY=wiFfYdH8Gipec8Eeeu0xXdbba9frFj0=OqFfea0dXdd9vqai=hGuQ8kuc9pgc9s8qqaq=dirpe0xb9q8qiLsFr0=vr0=vr0dc8meaabaqaciaacaGaaeqabaqabeGadaaakeaat0uy0HwzTfgDPnwy1egaryqtHrhAL1wy0L2yHvdaiqaacqWFlecsaaa@3762@.

All texts *T*_*n *_of length *n *are classified into classes depending on occurrences of different ℋ
 MathType@MTEF@5@5@+=feaafiart1ev1aaatCvAUfKttLearuWrP9MDH5MBPbIqV92AaeXatLxBI9gBaebbnrfifHhDYfgasaacH8akY=wiFfYdH8Gipec8Eeeu0xXdbba9frFj0=OqFfea0dXdd9vqai=hGuQ8kuc9pgc9s8qqaq=dirpe0xb9q8qiLsFr0=vr0=vr0dc8meaabaqaciaacaGaaeqabaqabeGadaaakeaat0uy0HwzTfgDPnwy1egaryqtHrhAL1wy0L2yHvdaiqaacqWFlecsaaa@3762@_*j*_. In this case it is difficult to introduce the target class *G*, since when the target number of occurrences *k*_*i *_is attained for some motif ℋ
 MathType@MTEF@5@5@+=feaafiart1ev1aaatCvAUfKttLearuWrP9MDH5MBPbIqV92AaeXatLxBI9gBaebbnrfifHhDYfgasaacH8akY=wiFfYdH8Gipec8Eeeu0xXdbba9frFj0=OqFfea0dXdd9vqai=hGuQ8kuc9pgc9s8qqaq=dirpe0xb9q8qiLsFr0=vr0=vr0dc8meaabaqaciaacaGaaeqabaqabeGadaaakeaat0uy0HwzTfgDPnwy1egaryqtHrhAL1wy0L2yHvdaiqaacqWFlecsaaa@3762@_*i*_, the corresponding value *k*_*j *_may not yet be attained for another motif ℋ
 MathType@MTEF@5@5@+=feaafiart1ev1aaatCvAUfKttLearuWrP9MDH5MBPbIqV92AaeXatLxBI9gBaebbnrfifHhDYfgasaacH8akY=wiFfYdH8Gipec8Eeeu0xXdbba9frFj0=OqFfea0dXdd9vqai=hGuQ8kuc9pgc9s8qqaq=dirpe0xb9q8qiLsFr0=vr0=vr0dc8meaabaqaciaacaGaaeqabaqabeGadaaakeaat0uy0HwzTfgDPnwy1egaryqtHrhAL1wy0L2yHvdaiqaacqWFlecsaaa@3762@_*j*_. Therefore we need to introduce the occurrence index of a set of motifs.

**Definition 2 ***Let the target number of occurrences of motif *ℋ
 MathType@MTEF@5@5@+=feaafiart1ev1aaatCvAUfKttLearuWrP9MDH5MBPbIqV92AaeXatLxBI9gBaebbnrfifHhDYfgasaacH8akY=wiFfYdH8Gipec8Eeeu0xXdbba9frFj0=OqFfea0dXdd9vqai=hGuQ8kuc9pgc9s8qqaq=dirpe0xb9q8qiLsFr0=vr0=vr0dc8meaabaqaciaacaGaaeqabaqabeGadaaakeaat0uy0HwzTfgDPnwy1egaryqtHrhAL1wy0L2yHvdaiqaacqWFlecsaaa@3762@_*i *_*be k*_*i*_. *Then, the occurrence index *Λ(k1,...,ks)
 MathType@MTEF@5@5@+=feaafiart1ev1aaatCvAUfKttLearuWrP9MDH5MBPbIqV92AaeXatLxBI9gBaebbnrfifHhDYfgasaacH8akY=wiFfYdH8Gipec8Eeeu0xXdbba9frFj0=OqFfea0dXdd9vqai=hGuQ8kuc9pgc9s8qqaq=dirpe0xb9q8qiLsFr0=vr0=vr0dc8meaabaqaciaacaGaaeqabaqabeGadaaakeaacqqHBoatdaWgaaWcbaGaeiikaGIaem4AaS2aaSbaaWqaaiabigdaXaqabaWccqGGSaalcqGGUaGlcqGGUaGlcqGGUaGlcqGGSaalcqWGRbWAdaWgaaadbaGaem4CamhabeaaliabcMcaPaqabaaaaa@39F8@ (*l*_1_, ..., *l*_*s*_) *of a set of motifs *(ℋ
 MathType@MTEF@5@5@+=feaafiart1ev1aaatCvAUfKttLearuWrP9MDH5MBPbIqV92AaeXatLxBI9gBaebbnrfifHhDYfgasaacH8akY=wiFfYdH8Gipec8Eeeu0xXdbba9frFj0=OqFfea0dXdd9vqai=hGuQ8kuc9pgc9s8qqaq=dirpe0xb9q8qiLsFr0=vr0=vr0dc8meaabaqaciaacaGaaeqabaqabeGadaaakeaat0uy0HwzTfgDPnwy1egaryqtHrhAL1wy0L2yHvdaiqaacqWFlecsaaa@3762@) *in the text T*_*n *_*containing l*_*i *_*possibly overlapping occurrences of each *ℋ
 MathType@MTEF@5@5@+=feaafiart1ev1aaatCvAUfKttLearuWrP9MDH5MBPbIqV92AaeXatLxBI9gBaebbnrfifHhDYfgasaacH8akY=wiFfYdH8Gipec8Eeeu0xXdbba9frFj0=OqFfea0dXdd9vqai=hGuQ8kuc9pgc9s8qqaq=dirpe0xb9q8qiLsFr0=vr0=vr0dc8meaabaqaciaacaGaaeqabaqabeGadaaakeaat0uy0HwzTfgDPnwy1egaryqtHrhAL1wy0L2yHvdaiqaacqWFlecsaaa@3762@_*i *_*is an s-vector the ith component of which can be calculated as follows*:

[Λ(k1,...,ks)(l1,...,ls)]i=λi={liifli≤ki,kiifli>ki.
 MathType@MTEF@5@5@+=feaafiart1ev1aaatCvAUfKttLearuWrP9MDH5MBPbIqV92AaeXatLxBI9gBaebbnrfifHhDYfgasaacH8akY=wiFfYdH8Gipec8Eeeu0xXdbba9frFj0=OqFfea0dXdd9vqai=hGuQ8kuc9pgc9s8qqaq=dirpe0xb9q8qiLsFr0=vr0=vr0dc8meaabaqaciaacaGaaeqabaqabeGadaaakeaacqGGBbWwcqqHBoatdaWgaaWcbaGaeiikaGIaem4AaS2aaSbaaWqaaiabigdaXaqabaWccqGGSaalcqGGUaGlcqGGUaGlcqGGUaGlcqGGSaalcqWGRbWAdaWgaaadbaGaem4CamhabeaaliabcMcaPaqabaGccqGGOaakcqWGSbaBdaWgaaWcbaGaeGymaedabeaakiabcYcaSiabc6caUiabc6caUiabc6caUiabcYcaSiabdYgaSnaaBaaaleaacqWGZbWCaeqaaOGaeiykaKIaeiyxa01aaSbaaSqaaiabdMgaPbqabaGccqGH9aqpiiGacqWF7oaBdaWgaaWcbaGaemyAaKgabeaakiabg2da9maaceqabaqbaeaabiWaaaqaaiabdYgaSnaaBaaaleaacqWGPbqAaeqaaaGcbaGaemyAaKMaemOzaygabaGaemiBaW2aaSbaaSqaaiabdMgaPbqabaGccqGHKjYOcqWGRbWAdaWgaaWcbaGaemyAaKgabeaakiabcYcaSaqaaiabdUgaRnaaBaaaleaacqWGPbqAaeqaaaGcbaGaemyAaKMaemOzaygabaGaemiBaW2aaSbaaSqaaiabdMgaPbqabaGccqGH+aGpcqWGRbWAdaWgaaWcbaGaemyAaKgabeaakiabc6caUaaaaiaawUhaaaaa@6BCA@

**Definition 3 ***A text T*_*i *_*belongs to class C*_*i *_(*λ*_1_, ..., *λ*_*s*_; *q*), 0 ≤ *λ*_*i *_≤ *k*_*i *_*iff*

*1. Length of T*_*i *_*equals i*,

*2. The occurrence index of motifs *(ℋ1,...,ℋs
 MathType@MTEF@5@5@+=feaafiart1ev1aaatCvAUfKttLearuWrP9MDH5MBPbIqV92AaeXatLxBI9gBaebbnrfifHhDYfgasaacH8akY=wiFfYdH8Gipec8Eeeu0xXdbba9frFj0=OqFfea0dXdd9vqai=hGuQ8kuc9pgc9s8qqaq=dirpe0xb9q8qiLsFr0=vr0=vr0dc8meaabaqaciaacaGaaeqabaqabeGadaaakeaat0uy0HwzTfgDPnwy1egaryqtHrhAL1wy0L2yHvdaiqaacqWFlecsdaWgaaWcbaGaeGymaedabeaakiabcYcaSiabc6caUiabc6caUiabc6caUiabcYcaSiab=TqiinaaBaaaleaacqWGZbWCaeqaaaaa@3F88@) *in text T*_*i *_*is equal to *(*λ*_1_, ..., *λ*_*s*_),

*3. A traversal AC *(T(ℋ)
 MathType@MTEF@5@5@+=feaafiart1ev1aaatCvAUfKttLearuWrP9MDH5MBPbIqV92AaeXatLxBI9gBaebbnrfifHhDYfgasaacH8akY=wiFfYdH8Gipec8Eeeu0xXdbba9frFj0=OqFfea0dXdd9vqai=hGuQ8kuc9pgc9s8qqaq=dirpe0xb9q8qiLsFr0=vr0=vr0dc8meaabaqaciaacaGaaeqabaqabeGadaaakeaat0uy0HwzTfgDPnwy1egaryqtHrhAL1wy0L2yHvdaiqaacqWFtepvcqGGOaakcqWFlecscqGGPaqkaaa@3AF1@, *T*_*i*_) *ends in node q*.

*A text T*_*i *_*belongs to class G*_*i *_(*k*_1_, ..., *k*_*s*_) *if it belongs to the union of classes*

Gi(k1,...,ks)=∪q∈ℋCi(k1,...,ks;q).
 MathType@MTEF@5@5@+=feaafiart1ev1aaatCvAUfKttLearuWrP9MDH5MBPbIqV92AaeXatLxBI9gBaebbnrfifHhDYfgasaacH8akY=wiFfYdH8Gipec8Eeeu0xXdbba9frFj0=OqFfea0dXdd9vqai=hGuQ8kuc9pgc9s8qqaq=dirpe0xb9q8qiLsFr0=vr0=vr0dc8meaabaqaciaacaGaaeqabaqabeGadaaakeaacqWGhbWrdaWgaaWcbaGaemyAaKgabeaakiabcIcaOiabdUgaRnaaBaaaleaacqaIXaqmaeqaaOGaeiilaWIaeiOla4IaeiOla4IaeiOla4IaeiilaWIaem4AaS2aaSbaaSqaaiabdohaZbqabaGccqGGPaqkcqGH9aqpdaWeqbqaaiabdoeadnaaBaaaleaacqWGPbqAaeqaaOGaeiikaGIaem4AaS2aaSbaaSqaaiabigdaXaqabaGccqGGSaalcqGGUaGlcqGGUaGlcqGGUaGlcqGGSaalcqWGRbWAdaWgaaWcbaGaem4CamhabeaakiabcUda7iabdghaXjabcMcaPaWcbaGaemyCaeNaeyicI48enfgDOvwBHrxAJfwnHbqeg0uy0HwzTfgDPnwy1aaceaGae83cHGeabeqdcqWIQisvaOGaeiOla4caaa@5CF8@

The desired *p*-value **P **(*L*_*n *_(ℋ1,...,ℋs
 MathType@MTEF@5@5@+=feaafiart1ev1aaatCvAUfKttLearuWrP9MDH5MBPbIqV92AaeXatLxBI9gBaebbnrfifHhDYfgasaacH8akY=wiFfYdH8Gipec8Eeeu0xXdbba9frFj0=OqFfea0dXdd9vqai=hGuQ8kuc9pgc9s8qqaq=dirpe0xb9q8qiLsFr0=vr0=vr0dc8meaabaqaciaacaGaaeqabaqabeGadaaakeaat0uy0HwzTfgDPnwy1egaryqtHrhAL1wy0L2yHvdaiqaacqWFlecsdaWgaaWcbaGaeGymaedabeaakiabcYcaSiabc6caUiabc6caUiabc6caUiabcYcaSiab=TqiinaaBaaaleaacqWGZbWCaeqaaaaa@3F88@; *k*_1_, ..., *k*_*s*_)) is equal to **P **(*G*_*n *_(*k*_1_, ..., *k*_*s*_)). The value is calculated iteratively. Again, we have a sum over all possible tree nodes *q *and symbols *a*. Now, *q'*, the image of the transition function *δ *(*q, a*) can belong simultaneously to several motifs {ℋ
 MathType@MTEF@5@5@+=feaafiart1ev1aaatCvAUfKttLearuWrP9MDH5MBPbIqV92AaeXatLxBI9gBaebbnrfifHhDYfgasaacH8akY=wiFfYdH8Gipec8Eeeu0xXdbba9frFj0=OqFfea0dXdd9vqai=hGuQ8kuc9pgc9s8qqaq=dirpe0xb9q8qiLsFr0=vr0=vr0dc8meaabaqaciaacaGaaeqabaqabeGadaaakeaat0uy0HwzTfgDPnwy1egaryqtHrhAL1wy0L2yHvdaiqaacqWFlecsaaa@3762@_*j*_}_1≤*j*≤*s*_. Thus, the resulting probability **P **(*C*_*i*+1 _(*λ*_1_, ..., *λ*_*s*_; *q'*)) that text *T*_*i*+1 _belongs to class *C*_*i*+1 _(*λ*_1_, ..., *λ*_*s*_; *q'*) calculates as

P(Ci+1(λ1,...,λs;q′))=∑(q,a):δ(q,a)=q′∑(r1,...,rs)∈JP(Ci(r1,...,rs;q))⋅p(a)
 MathType@MTEF@5@5@+=feaafiart1ev1aaatCvAUfKttLearuWrP9MDH5MBPbIqV92AaeXatLxBI9gBaebbnrfifHhDYfgasaacH8akY=wiFfYdH8Gipec8Eeeu0xXdbba9frFj0=OqFfea0dXdd9vqai=hGuQ8kuc9pgc9s8qqaq=dirpe0xb9q8qiLsFr0=vr0=vr0dc8meaabaqaciaacaGaaeqabaqabeGadaaakeaaieqacqWFqbaucqGGOaakcqWGdbWqdaWgaaWcbaGaemyAaKMaey4kaSIaeGymaedabeaakiabcIcaOGGaciab+T7aSnaaBaaaleaacqaIXaqmaeqaaOGaeiilaWIaeiOla4IaeiOla4IaeiOla4IaeiilaWIae43UdW2aaSbaaSqaaiabdohaZbqabaGccqGG7aWocuWGXbqCgaqbaiabcMcaPiabcMcaPiabg2da9maaqafabaWaaabuaeaacqWFqbaucqGGOaakcqWGdbWqdaWgaaWcbaGaemyAaKgabeaakiabcIcaOiabdkhaYnaaBaaaleaacqaIXaqmaeqaaOGaeiilaWIaeiOla4IaeiOla4IaeiOla4IaeiilaWIaemOCai3aaSbaaSqaaiabdohaZbqabaGccqGG7aWocqWGXbqCcqGGPaqkcqGGPaqkcqGHflY1cqWGWbaCcqGGOaakcqWGHbqycqGGPaqkaSqaaiabcIcaOiabdkhaYnaaBaaameaacqaIXaqmaeqaaSGaeiilaWIaeiOla4IaeiOla4IaeiOla4IaeiilaWIaemOCai3aaSbaaWqaaiabdohaZbqabaWccqGGPaqkcqGHiiIZcqWFkbGsaeqaniabggHiLdaaleaacqGGOaakcqWGXbqCcqGGSaalcqWGHbqycqGGPaqkcqGG6aGocqGF0oazcqGGOaakcqWGXbqCcqGGSaalcqWGHbqycqGGPaqkcqGH9aqpcuWGXbqCgaqbaaqab0GaeyyeIuoaaaa@8070@

where the summation in the second sum is performed over all allowed *s*-tuples of indexes (*r*_1_, ..., *r*_*s*_) which together make the set of *s*-tuples **J**. A *s*-tuple of indexes (*r*_1_, ..., *r*_*s*_) belongs to **J **if it complies with the following conditions:

1. if *q' *∉ ℋ
 MathType@MTEF@5@5@+=feaafiart1ev1aaatCvAUfKttLearuWrP9MDH5MBPbIqV92AaeXatLxBI9gBaebbnrfifHhDYfgasaacH8akY=wiFfYdH8Gipec8Eeeu0xXdbba9frFj0=OqFfea0dXdd9vqai=hGuQ8kuc9pgc9s8qqaq=dirpe0xb9q8qiLsFr0=vr0=vr0dc8meaabaqaciaacaGaaeqabaqabeGadaaakeaat0uy0HwzTfgDPnwy1egaryqtHrhAL1wy0L2yHvdaiqaacqWFlecsaaa@3762@_*j *_then *r*_*j *_= *λ*_*j*_,

2. if *q' *ℋ
 MathType@MTEF@5@5@+=feaafiart1ev1aaatCvAUfKttLearuWrP9MDH5MBPbIqV92AaeXatLxBI9gBaebbnrfifHhDYfgasaacH8akY=wiFfYdH8Gipec8Eeeu0xXdbba9frFj0=OqFfea0dXdd9vqai=hGuQ8kuc9pgc9s8qqaq=dirpe0xb9q8qiLsFr0=vr0=vr0dc8meaabaqaciaacaGaaeqabaqabeGadaaakeaat0uy0HwzTfgDPnwy1egaryqtHrhAL1wy0L2yHvdaiqaacqWFlecsaaa@3762@_*j *_and *λ*_*j *_<*k*_*j *_then *r*_*j *_= *λ*_*j *_- 1,

3. if *q' *∈ ℋ
 MathType@MTEF@5@5@+=feaafiart1ev1aaatCvAUfKttLearuWrP9MDH5MBPbIqV92AaeXatLxBI9gBaebbnrfifHhDYfgasaacH8akY=wiFfYdH8Gipec8Eeeu0xXdbba9frFj0=OqFfea0dXdd9vqai=hGuQ8kuc9pgc9s8qqaq=dirpe0xb9q8qiLsFr0=vr0=vr0dc8meaabaqaciaacaGaaeqabaqabeGadaaakeaat0uy0HwzTfgDPnwy1egaryqtHrhAL1wy0L2yHvdaiqaacqWFlecsaaa@3762@_*j *_and *λ*_*j *_= *k*_*j *_then *r*_*j *_= *k*_*j *_or *r*_*j *_= *k*_*j *_- 1.

### Implementation details

Our basic data structure is the prefix tree; we use its standard representation [42] [see also Additional files 2 and 3 for *Tree construction from PWM motif representation*]. Each tree node *q *∈ Qℋ
 MathType@MTEF@5@5@+=feaafiart1ev1aaatCvAUfKttLearuWrP9MDH5MBPbIqV92AaeXatLxBI9gBaebbnrfifHhDYfgasaacH8akY=wiFfYdH8Gipec8Eeeu0xXdbba9frFj0=OqFfea0dXdd9vqai=hGuQ8kuc9pgc9s8qqaq=dirpe0xb9q8qiLsFr0=vr0=vr0dc8meaabaqaciaacaGaaeqabaqabeGadaaakeaacqWGrbqudaWgaaWcbaWenfgDOvwBHrxAJfwnHbqeg0uy0HwzTfgDPnwy1aaceaGae83cHGeabeaaaaa@38B9@ is supplied with several additional variables.

At stage (*i *+ 1) of probability computation the values **P **(*C*_*i*+1 _(*λ*_1_, ..., *λ*_*s*_; *q*)) become computed from the values **P **(*C*_*i *_(*λ*_1_, ..., *λ*_*s*_; *q*)) obtained at the previous stage of induction. Therefore, at stage (*i *+ 1), one no longer needs the values calculated at stage (*i *- 1). Thus, each node is supplied with two *k*_1 _× ⋯ × *k*_*s*_-arrays of real values **C**_**0 **_and **C**_**1 **_for storing **P **(*C*_*i *_(*λ*_1_, ..., *λ*_*s*_; *q*)) and **P **(*C*_*i*+1 _(*λ*_1_, ..., *λ*_*s*_; *q*)) for different *λ*_*j*_. **C**_**0 **_is used to store probabilities for even text lengths while **C**_**1 **_for odd.

In implementation the calculation of values **P **(*C*_*i*+1 _(*λ*_1_, ..., *λ*_*s*_; *q'*)) from **P **(*C*_*i *_(*λ*_1_, ..., *λ*_*s*_; *q*)) for all *q'*, *q *∈ Qℋ
 MathType@MTEF@5@5@+=feaafiart1ev1aaatCvAUfKttLearuWrP9MDH5MBPbIqV92AaeXatLxBI9gBaebbnrfifHhDYfgasaacH8akY=wiFfYdH8Gipec8Eeeu0xXdbba9frFj0=OqFfea0dXdd9vqai=hGuQ8kuc9pgc9s8qqaq=dirpe0xb9q8qiLsFr0=vr0=vr0dc8meaabaqaciaacaGaaeqabaqabeGadaaakeaacqWGrbqudaWgaaWcbaWenfgDOvwBHrxAJfwnHbqeg0uy0HwzTfgDPnwy1aaceaGae83cHGeabeaaaaa@38B9@ and (*λ*_1_, ..., *λ*_*s*_): 0 ≤ *λ*_*j *_≤ *k*_*j*_, 1 ≤ *j *≤ *s*, is performed in the parallel way. Initially we set all the values **P **(*C*_*i*+1 _(*λ*_1_, ..., *λ*_*s*_; *q'*)) to 0. Then we look over all tuples (*r*_1_, ..., *r*_*s*_; *q*), where *q *∈ Qℋ
 MathType@MTEF@5@5@+=feaafiart1ev1aaatCvAUfKttLearuWrP9MDH5MBPbIqV92AaeXatLxBI9gBaebbnrfifHhDYfgasaacH8akY=wiFfYdH8Gipec8Eeeu0xXdbba9frFj0=OqFfea0dXdd9vqai=hGuQ8kuc9pgc9s8qqaq=dirpe0xb9q8qiLsFr0=vr0=vr0dc8meaabaqaciaacaGaaeqabaqabeGadaaakeaacqWGrbqudaWgaaWcbaWenfgDOvwBHrxAJfwnHbqeg0uy0HwzTfgDPnwy1aaceaGae83cHGeabeaaaaa@38B9@ and (*r*_1_, ..., *r*_*s*_): 0 ≤ *r*_*j *_≤ *k*_*j*_, 1 ≤ *j *≤ *s*. For each tuple (*r*_1_, ..., *r*_*s*_; *q*) and all letters *a *∈ Σ we find the prefix *q' *= *δ *(*q, a*) and the value **P **(*C*_*i *_(*r*_1_, ..., *r*_*s*_; *q*))·*p*(*a*). Then we add **P **(*C*_*i *_(*r*_1_, ..., *r*_*s*_; *q*))·*p*(*a*) to the value **P **(*C*_*i*+1 _(*λ*_1_, ..., *λ*_*s*_; *q'*)) where (*λ*_1_, ..., *λ*_*s*_; *q'*) meet the conditions inverse to those of formula (11):

1. if *q' *∉ ℋ
 MathType@MTEF@5@5@+=feaafiart1ev1aaatCvAUfKttLearuWrP9MDH5MBPbIqV92AaeXatLxBI9gBaebbnrfifHhDYfgasaacH8akY=wiFfYdH8Gipec8Eeeu0xXdbba9frFj0=OqFfea0dXdd9vqai=hGuQ8kuc9pgc9s8qqaq=dirpe0xb9q8qiLsFr0=vr0=vr0dc8meaabaqaciaacaGaaeqabaqabeGadaaakeaat0uy0HwzTfgDPnwy1egaryqtHrhAL1wy0L2yHvdaiqaacqWFlecsaaa@3762@_*j *_then *λ*_*j *_= *r*_*j*_,

2. if *q' *∈ ℋ
 MathType@MTEF@5@5@+=feaafiart1ev1aaatCvAUfKttLearuWrP9MDH5MBPbIqV92AaeXatLxBI9gBaebbnrfifHhDYfgasaacH8akY=wiFfYdH8Gipec8Eeeu0xXdbba9frFj0=OqFfea0dXdd9vqai=hGuQ8kuc9pgc9s8qqaq=dirpe0xb9q8qiLsFr0=vr0=vr0dc8meaabaqaciaacaGaaeqabaqabeGadaaakeaat0uy0HwzTfgDPnwy1egaryqtHrhAL1wy0L2yHvdaiqaacqWFlecsaaa@3762@_*j *_and *r*_*j *_<*k*_*j *_then *λ*_*j *_= *r*_*j *_+ 1,

3. if *q' *∈ ℋ
 MathType@MTEF@5@5@+=feaafiart1ev1aaatCvAUfKttLearuWrP9MDH5MBPbIqV92AaeXatLxBI9gBaebbnrfifHhDYfgasaacH8akY=wiFfYdH8Gipec8Eeeu0xXdbba9frFj0=OqFfea0dXdd9vqai=hGuQ8kuc9pgc9s8qqaq=dirpe0xb9q8qiLsFr0=vr0=vr0dc8meaabaqaciaacaGaaeqabaqabeGadaaakeaat0uy0HwzTfgDPnwy1egaryqtHrhAL1wy0L2yHvdaiqaacqWFlecsaaa@3762@_*j *_and *r*_*j *_= *k*_*j *_then *λ*_*j *_= *r*_*j*_.

At the stage *i *= *n *the desired *p*-value is the sum

P(Gn(k1,...,ks))=∑q∈ℋP(Cn(k1,...,ks;q)).
 MathType@MTEF@5@5@+=feaafiart1ev1aaatCvAUfKttLearuWrP9MDH5MBPbIqV92AaeXatLxBI9gBaebbnrfifHhDYfgasaacH8akY=wiFfYdH8Gipec8Eeeu0xXdbba9frFj0=OqFfea0dXdd9vqai=hGuQ8kuc9pgc9s8qqaq=dirpe0xb9q8qiLsFr0=vr0=vr0dc8meaabaqaciaacaGaaeqabaqabeGadaaakeaaieqacqWFqbaucqGGOaakcqWGhbWrdaWgaaWcbaGaemOBa4gabeaakiabcIcaOiabdUgaRnaaBaaaleaacqaIXaqmaeqaaOGaeiilaWIaeiOla4IaeiOla4IaeiOla4IaeiilaWIaem4AaS2aaSbaaSqaaiabdohaZbqabaGccqGGPaqkcqGGPaqkcqGH9aqpdaaeqbqaaiab=bfaqjabcIcaOiabdoeadnaaBaaaleaacqWGUbGBaeqaaOGaeiikaGIaem4AaS2aaSbaaSqaaiabigdaXaqabaGccqGGSaalcqGGUaGlcqGGUaGlcqGGUaGlcqGGSaalcqWGRbWAdaWgaaWcbaGaem4CamhabeaakiabcUda7iabdghaXjabcMcaPiabcMcaPiabc6caUaWcbaGaemyCaeNaeyicI48enfgDOvwBHrxAJfwnHbqeg0uy0HwzTfgDPnwy1aaceaGae43cHGeabeqdcqGHris5aaaa@6328@

### Markov text model

Tree approach and the recursion (11) can be readily extended to calculate *p*-values of motif occurrences in random texts generated by the Markov model of order *K*. Given the order *K *of the Markov model, the probability *p*(*a*) in (11) depends on *K *previous letters. Thus, if the length |*q*| of the prefix *q *is less than *K*, one cannot calculate *p*(*a*) knowing only the prefix *q*. To overcome this we divide each class *C*_*i *_(*r*_1_, ..., *r*_*s*_; *q*), where |*q*| = *d *<*min *(*K*, *i*) into subclasses *C*_*i *_(*r*_1_, ..., *r*_*s*_; *q*, *w*); each subclass corresponds to a word *w *of length *min *(*K*, *i*) - *d*. Then, a text *T*_*i *_of length *i *belongs to class *C*_*i *_(*r*_1_, ..., *r*_*s*_; *q*, *w*) if the suffix of *T*_*i *_of length *min *(*K*, *i*) equals to *w*·*q*.

**Figure 2:**
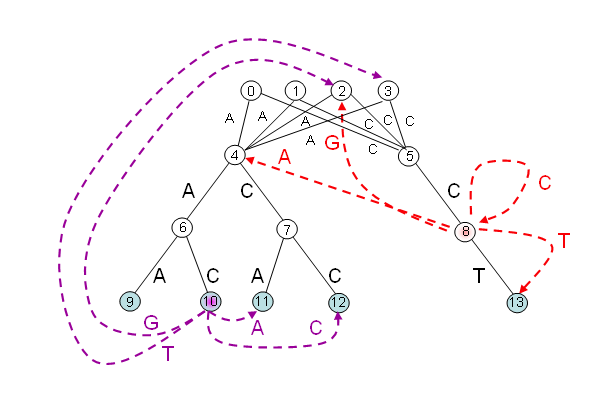
**Tree T(ℋ)
 MathType@MTEF@5@5@+=feaafiart1ev1aaatCvAUfKttLearuWrP9MDH5MBPbIqV92AaeXatLxBI9gBaebbnrfifHhDYfgasaacH8akY=wiFfYdH8Gipec8Eeeu0xXdbba9frFj0=OqFfea0dXdd9vqai=hGuQ8kuc9pgc9s8qqaq=dirpe0xb9q8qiLsFr0=vr0=vr0dc8meaabaqaciaacaGaaeqabaqabeGadaaakeaat0uy0HwzTfgDPnwy1egaryqtHrhAL1wy0L2yHvdaiqaacqWFtepvcqGGOaakcqWFlecscqGGPaqkaaa@3AF1@ for the set ℋ
 MathType@MTEF@5@5@+=feaafiart1ev1aaatCvAUfKttLearuWrP9MDH5MBPbIqV92AaeXatLxBI9gBaebbnrfifHhDYfgasaacH8akY=wiFfYdH8Gipec8Eeeu0xXdbba9frFj0=OqFfea0dXdd9vqai=hGuQ8kuc9pgc9s8qqaq=dirpe0xb9q8qiLsFr0=vr0=vr0dc8meaabaqaciaacaGaaeqabaqabeGadaaakeaat0uy0HwzTfgDPnwy1egaryqtHrhAL1wy0L2yHvdaiqaacqWFlecsaaa@3762@ = {aaa, aac, aca, acc, cct} with dashed links for *δ *function under Markov(1) model**. Tree T(ℋ)
 MathType@MTEF@5@5@+=feaafiart1ev1aaatCvAUfKttLearuWrP9MDH5MBPbIqV92AaeXatLxBI9gBaebbnrfifHhDYfgasaacH8akY=wiFfYdH8Gipec8Eeeu0xXdbba9frFj0=OqFfea0dXdd9vqai=hGuQ8kuc9pgc9s8qqaq=dirpe0xb9q8qiLsFr0=vr0=vr0dc8meaabaqaciaacaGaaeqabaqabeGadaaakeaat0uy0HwzTfgDPnwy1egaryqtHrhAL1wy0L2yHvdaiqaacqWFtepvcqGGOaakcqWFlecscqGGPaqkaaa@3AF1@ for the set ℋ
 MathType@MTEF@5@5@+=feaafiart1ev1aaatCvAUfKttLearuWrP9MDH5MBPbIqV92AaeXatLxBI9gBaebbnrfifHhDYfgasaacH8akY=wiFfYdH8Gipec8Eeeu0xXdbba9frFj0=OqFfea0dXdd9vqai=hGuQ8kuc9pgc9s8qqaq=dirpe0xb9q8qiLsFr0=vr0=vr0dc8meaabaqaciaacaGaaeqabaqabeGadaaakeaat0uy0HwzTfgDPnwy1egaryqtHrhAL1wy0L2yHvdaiqaacqWFlecsaaa@3762@ = {AAA, AAC, ACA, ACC, CCT} under Markov model of order 1. Dashed colored links represent *δ *function for internal node (8) – in red, and for marked node (10) corresponding to the word AAC ∈ ℋ
 MathType@MTEF@5@5@+=feaafiart1ev1aaatCvAUfKttLearuWrP9MDH5MBPbIqV92AaeXatLxBI9gBaebbnrfifHhDYfgasaacH8akY=wiFfYdH8Gipec8Eeeu0xXdbba9frFj0=OqFfea0dXdd9vqai=hGuQ8kuc9pgc9s8qqaq=dirpe0xb9q8qiLsFr0=vr0=vr0dc8meaabaqaciaacaGaaeqabaqabeGadaaakeaat0uy0HwzTfgDPnwy1egaryqtHrhAL1wy0L2yHvdaiqaacqWFlecsaaa@3762@ – in purple.

Figure 2 gives an example for Markov model of order *K *= 1. The tree is constructed for the set ℋ
 MathType@MTEF@5@5@+=feaafiart1ev1aaatCvAUfKttLearuWrP9MDH5MBPbIqV92AaeXatLxBI9gBaebbnrfifHhDYfgasaacH8akY=wiFfYdH8Gipec8Eeeu0xXdbba9frFj0=OqFfea0dXdd9vqai=hGuQ8kuc9pgc9s8qqaq=dirpe0xb9q8qiLsFr0=vr0=vr0dc8meaabaqaciaacaGaaeqabaqabeGadaaakeaat0uy0HwzTfgDPnwy1egaryqtHrhAL1wy0L2yHvdaiqaacqWFlecsaaa@3762@ = {AAA, AAC, ACA, ACC, CCT}. The text *T *= ATGCCAACCTT produces the following sequence of nodes {*q*_*i*_}_*i*≥1 _(the numbers of the corresponding nodes in Figure 2 are shown in square brackets): A[4], (*ε, T*)[3], (*ε, G*)[2], C[5], CC[8], A[4], AA[6], AAC[10], ACC[12], CCT[13], (*ε, T*)[3].

The recursive equations for probabilities **P **(*L*_*n *_(ℋ
 MathType@MTEF@5@5@+=feaafiart1ev1aaatCvAUfKttLearuWrP9MDH5MBPbIqV92AaeXatLxBI9gBaebbnrfifHhDYfgasaacH8akY=wiFfYdH8Gipec8Eeeu0xXdbba9frFj0=OqFfea0dXdd9vqai=hGuQ8kuc9pgc9s8qqaq=dirpe0xb9q8qiLsFr0=vr0=vr0dc8meaabaqaciaacaGaaeqabaqabeGadaaakeaat0uy0HwzTfgDPnwy1egaryqtHrhAL1wy0L2yHvdaiqaacqWFlecsaaa@3762@; 1)), **P **(*L*_*n *_(ℋ
 MathType@MTEF@5@5@+=feaafiart1ev1aaatCvAUfKttLearuWrP9MDH5MBPbIqV92AaeXatLxBI9gBaebbnrfifHhDYfgasaacH8akY=wiFfYdH8Gipec8Eeeu0xXdbba9frFj0=OqFfea0dXdd9vqai=hGuQ8kuc9pgc9s8qqaq=dirpe0xb9q8qiLsFr0=vr0=vr0dc8meaabaqaciaacaGaaeqabaqabeGadaaakeaat0uy0HwzTfgDPnwy1egaryqtHrhAL1wy0L2yHvdaiqaacqWFlecsaaa@3762@; *k*)), and **P **(*L*_*n *_(ℋ1,...,ℋs
 MathType@MTEF@5@5@+=feaafiart1ev1aaatCvAUfKttLearuWrP9MDH5MBPbIqV92AaeXatLxBI9gBaebbnrfifHhDYfgasaacH8akY=wiFfYdH8Gipec8Eeeu0xXdbba9frFj0=OqFfea0dXdd9vqai=hGuQ8kuc9pgc9s8qqaq=dirpe0xb9q8qiLsFr0=vr0=vr0dc8meaabaqaciaacaGaaeqabaqabeGadaaakeaat0uy0HwzTfgDPnwy1egaryqtHrhAL1wy0L2yHvdaiqaacqWFlecsdaWgaaWcbaGaeGymaedabeaakiabcYcaSiabc6caUiabc6caUiabc6caUiabcYcaSiab=TqiinaaBaaaleaacqWGZbWCaeqaaaaa@3F88@; *k*_1_, ..., *k*_*s*_)) can be obtained from the corresponding formulae (7-8), (11–13) and (16) by substituting probabilities *p*(*a*) with *p*(*a*|*t*[1] ⋯ *t *[*K*]), where

t[1]⋯t[K]={w⋅qif 0≤d<K,K-suffix of qotherwise.
 MathType@MTEF@5@5@+=feaafiart1ev1aaatCvAUfKttLearuWrP9MDH5MBPbIqV92AaeXatLxBI9gBaebbnrfifHhDYfgasaacH8akY=wiFfYdH8Gipec8Eeeu0xXdbba9frFj0=OqFfea0dXdd9vqai=hGuQ8kuc9pgc9s8qqaq=dirpe0xb9q8qiLsFr0=vr0=vr0dc8meaabaqaciaacaGaaeqabaqabeGadaaakeaacqWG0baDcqGGBbWwcqaIXaqmcqGGDbqxcqWIVlctcqWG0baDcqGGBbWwcqWGlbWscqGGDbqxcqGH9aqpdaGabeqaauaabaqaciaaaeaacqWG3bWDcqGHflY1cqWGXbqCaeaacqqGPbqAcqqGMbGzcqqGGaaicqaIWaamcqGHKjYOcqWGKbazcqGH8aapcqWGlbWscqGGSaalaeaacqWGlbWscqqGTaqlcqqGZbWCcqqG1bqDcqqGMbGzcqqGMbGzcqqGPbqAcqqG4baEcqqGGaaicqqGVbWBcqqGMbGzcqqGGaaicqWGXbqCaeaacqqGVbWBcqqG0baDcqqGObaAcqqGLbqzcqqGYbGCcqqG3bWDcqqGPbqAcqqGZbWCcqqGLbqzcqGGUaGlaaaacaGL7baaaaa@67AD@

The Markov extension is currently implemented for *K *= 1.

### Complexity

To resume, the computation of **P **(*L*_*n *_(ℋ
 MathType@MTEF@5@5@+=feaafiart1ev1aaatCvAUfKttLearuWrP9MDH5MBPbIqV92AaeXatLxBI9gBaebbnrfifHhDYfgasaacH8akY=wiFfYdH8Gipec8Eeeu0xXdbba9frFj0=OqFfea0dXdd9vqai=hGuQ8kuc9pgc9s8qqaq=dirpe0xb9q8qiLsFr0=vr0=vr0dc8meaabaqaciaacaGaaeqabaqabeGadaaakeaat0uy0HwzTfgDPnwy1egaryqtHrhAL1wy0L2yHvdaiqaacqWFlecsaaa@3762@; *k*)) for one set ℋ
 MathType@MTEF@5@5@+=feaafiart1ev1aaatCvAUfKttLearuWrP9MDH5MBPbIqV92AaeXatLxBI9gBaebbnrfifHhDYfgasaacH8akY=wiFfYdH8Gipec8Eeeu0xXdbba9frFj0=OqFfea0dXdd9vqai=hGuQ8kuc9pgc9s8qqaq=dirpe0xb9q8qiLsFr0=vr0=vr0dc8meaabaqaciaacaGaaeqabaqabeGadaaakeaat0uy0HwzTfgDPnwy1egaryqtHrhAL1wy0L2yHvdaiqaacqWFlecsaaa@3762@ requires a computation of (P(Ci(l,q)))0≤l<k,q∈Qℋ
 MathType@MTEF@5@5@+=feaafiart1ev1aaatCvAUfKttLearuWrP9MDH5MBPbIqV92AaeXatLxBI9gBaebbnrfifHhDYfgasaacH8akY=wiFfYdH8Gipec8Eeeu0xXdbba9frFj0=OqFfea0dXdd9vqai=hGuQ8kuc9pgc9s8qqaq=dirpe0xb9q8qiLsFr0=vr0=vr0dc8meaabaqaciaacaGaaeqabaqabeGadaaakeaadaqadaqaaGqabiab=bfaqjabcIcaOiabdoeadnaaBaaaleaacqWGPbqAaeqaaOGaeiikaGIaemiBaWMaeiilaWIaemyCaeNaeiykaKIaeiykaKcacaGLOaGaayzkaaWaaSbaaSqaaiabicdaWiabgsMiJkabdYgaSjabgYda8iabdUgaRjabcYcaSiabdghaXjabgIGiolabdgfarnaaBaaameaat0uy0HwzTfgDPnwy1egaryqtHrhAL1wy0L2yHvdaiqaacqGFlecsaeqaaaWcbeaaaaa@4F8E@ for *i *≤ *n*. For each iteration, the time complexity is *O *(*k*|Qℋ
 MathType@MTEF@5@5@+=feaafiart1ev1aaatCvAUfKttLearuWrP9MDH5MBPbIqV92AaeXatLxBI9gBaebbnrfifHhDYfgasaacH8akY=wiFfYdH8Gipec8Eeeu0xXdbba9frFj0=OqFfea0dXdd9vqai=hGuQ8kuc9pgc9s8qqaq=dirpe0xb9q8qiLsFr0=vr0=vr0dc8meaabaqaciaacaGaaeqabaqabeGadaaakeaacqWGrbqudaWgaaWcbaWenfgDOvwBHrxAJfwnHbqeg0uy0HwzTfgDPnwy1aaceaGae83cHGeabeaaaaa@38B9@| |Σ|), where |Σ| is the size of the alphabet. One traverses the tree *n *times. As |Qℋ
 MathType@MTEF@5@5@+=feaafiart1ev1aaatCvAUfKttLearuWrP9MDH5MBPbIqV92AaeXatLxBI9gBaebbnrfifHhDYfgasaacH8akY=wiFfYdH8Gipec8Eeeu0xXdbba9frFj0=OqFfea0dXdd9vqai=hGuQ8kuc9pgc9s8qqaq=dirpe0xb9q8qiLsFr0=vr0=vr0dc8meaabaqaciaacaGaaeqabaqabeGadaaakeaacqWGrbqudaWgaaWcbaWenfgDOvwBHrxAJfwnHbqeg0uy0HwzTfgDPnwy1aaceaGae83cHGeabeaaaaa@38B9@| is upper bounded by (*m*|ℋ
 MathType@MTEF@5@5@+=feaafiart1ev1aaatCvAUfKttLearuWrP9MDH5MBPbIqV92AaeXatLxBI9gBaebbnrfifHhDYfgasaacH8akY=wiFfYdH8Gipec8Eeeu0xXdbba9frFj0=OqFfea0dXdd9vqai=hGuQ8kuc9pgc9s8qqaq=dirpe0xb9q8qiLsFr0=vr0=vr0dc8meaabaqaciaacaGaaeqabaqabeGadaaakeaat0uy0HwzTfgDPnwy1egaryqtHrhAL1wy0L2yHvdaiqaacqWFlecsaaa@3762@|), where *m *is the maximal length of word in ℋ
 MathType@MTEF@5@5@+=feaafiart1ev1aaatCvAUfKttLearuWrP9MDH5MBPbIqV92AaeXatLxBI9gBaebbnrfifHhDYfgasaacH8akY=wiFfYdH8Gipec8Eeeu0xXdbba9frFj0=OqFfea0dXdd9vqai=hGuQ8kuc9pgc9s8qqaq=dirpe0xb9q8qiLsFr0=vr0=vr0dc8meaabaqaciaacaGaaeqabaqabeGadaaakeaat0uy0HwzTfgDPnwy1egaryqtHrhAL1wy0L2yHvdaiqaacqWFlecsaaa@3762@, this yields the overall *O *(*nkm*|ℋ
 MathType@MTEF@5@5@+=feaafiart1ev1aaatCvAUfKttLearuWrP9MDH5MBPbIqV92AaeXatLxBI9gBaebbnrfifHhDYfgasaacH8akY=wiFfYdH8Gipec8Eeeu0xXdbba9frFj0=OqFfea0dXdd9vqai=hGuQ8kuc9pgc9s8qqaq=dirpe0xb9q8qiLsFr0=vr0=vr0dc8meaabaqaciaacaGaaeqabaqabeGadaaakeaat0uy0HwzTfgDPnwy1egaryqtHrhAL1wy0L2yHvdaiqaacqWFlecsaaa@3762@||Σ|) time complexity and a *O *(*km*|ℋ
 MathType@MTEF@5@5@+=feaafiart1ev1aaatCvAUfKttLearuWrP9MDH5MBPbIqV92AaeXatLxBI9gBaebbnrfifHhDYfgasaacH8akY=wiFfYdH8Gipec8Eeeu0xXdbba9frFj0=OqFfea0dXdd9vqai=hGuQ8kuc9pgc9s8qqaq=dirpe0xb9q8qiLsFr0=vr0=vr0dc8meaabaqaciaacaGaaeqabaqabeGadaaakeaat0uy0HwzTfgDPnwy1egaryqtHrhAL1wy0L2yHvdaiqaacqWFlecsaaa@3762@|) space complexity.

When several sets are involved, the number of nodes in the tree T(ℋ1∪⋯∪ℋs)
 MathType@MTEF@5@5@+=feaafiart1ev1aaatCvAUfKttLearuWrP9MDH5MBPbIqV92AaeXatLxBI9gBaebbnrfifHhDYfgasaacH8akY=wiFfYdH8Gipec8Eeeu0xXdbba9frFj0=OqFfea0dXdd9vqai=hGuQ8kuc9pgc9s8qqaq=dirpe0xb9q8qiLsFr0=vr0=vr0dc8meaabaqaciaacaGaaeqabaqabeGadaaakeaat0uy0HwzTfgDPnwy1egaryqtHrhAL1wy0L2yHvdaiqaacqWFtepvcqGGOaakcqWFlecsdaWgaaWcbaGaeGymaedabeaakiabgQIiilabl+UimjabgQIiilab=TqiinaaBaaaleaacqWGZbWCaeqaaOGaeiykaKcaaa@43E3@ becomes *O *(*m*|ℋ
 MathType@MTEF@5@5@+=feaafiart1ev1aaatCvAUfKttLearuWrP9MDH5MBPbIqV92AaeXatLxBI9gBaebbnrfifHhDYfgasaacH8akY=wiFfYdH8Gipec8Eeeu0xXdbba9frFj0=OqFfea0dXdd9vqai=hGuQ8kuc9pgc9s8qqaq=dirpe0xb9q8qiLsFr0=vr0=vr0dc8meaabaqaciaacaGaaeqabaqabeGadaaakeaat0uy0HwzTfgDPnwy1egaryqtHrhAL1wy0L2yHvdaiqaacqWFlecsaaa@3762@|) with *m *equal to the maximal length of word in ℋ=ℋ1∪⋯∪ℋs
 MathType@MTEF@5@5@+=feaafiart1ev1aaatCvAUfKttLearuWrP9MDH5MBPbIqV92AaeXatLxBI9gBaebbnrfifHhDYfgasaacH8akY=wiFfYdH8Gipec8Eeeu0xXdbba9frFj0=OqFfea0dXdd9vqai=hGuQ8kuc9pgc9s8qqaq=dirpe0xb9q8qiLsFr0=vr0=vr0dc8meaabaqaciaacaGaaeqabaqabeGadaaakeaat0uy0HwzTfgDPnwy1egaryqtHrhAL1wy0L2yHvdaiqaacqWFlecscqGH9aqpcqWFlecsdaWgaaWcbaGaeGymaedabeaakiabgQIiilabl+UimjabgQIiilab=TqiinaaBaaaleaacqWGZbWCaeqaaaaa@4249@. Additional memory in each node is ∏_*i *_*k*_*i*_. Therefore, the time complexity is *O *(*nm*|Σ|∏_*i *_*k*_*i*_|ℋ
 MathType@MTEF@5@5@+=feaafiart1ev1aaatCvAUfKttLearuWrP9MDH5MBPbIqV92AaeXatLxBI9gBaebbnrfifHhDYfgasaacH8akY=wiFfYdH8Gipec8Eeeu0xXdbba9frFj0=OqFfea0dXdd9vqai=hGuQ8kuc9pgc9s8qqaq=dirpe0xb9q8qiLsFr0=vr0=vr0dc8meaabaqaciaacaGaaeqabaqabeGadaaakeaat0uy0HwzTfgDPnwy1egaryqtHrhAL1wy0L2yHvdaiqaacqWFlecsaaa@3762@|) and the space complexity is *O *(*m *∏_*i *_*k*_*i *_|ℋ
 MathType@MTEF@5@5@+=feaafiart1ev1aaatCvAUfKttLearuWrP9MDH5MBPbIqV92AaeXatLxBI9gBaebbnrfifHhDYfgasaacH8akY=wiFfYdH8Gipec8Eeeu0xXdbba9frFj0=OqFfea0dXdd9vqai=hGuQ8kuc9pgc9s8qqaq=dirpe0xb9q8qiLsFr0=vr0=vr0dc8meaabaqaciaacaGaaeqabaqabeGadaaakeaat0uy0HwzTfgDPnwy1egaryqtHrhAL1wy0L2yHvdaiqaacqWFlecsaaa@3762@|). In the Markov model of order *K*, one memorizes |Σ|^*K - d *^predecessors for each node at depth *d*, 0 = *d *<*K*. In other words, the number of classes becomes (*m*|ℋ
 MathType@MTEF@5@5@+=feaafiart1ev1aaatCvAUfKttLearuWrP9MDH5MBPbIqV92AaeXatLxBI9gBaebbnrfifHhDYfgasaacH8akY=wiFfYdH8Gipec8Eeeu0xXdbba9frFj0=OqFfea0dXdd9vqai=hGuQ8kuc9pgc9s8qqaq=dirpe0xb9q8qiLsFr0=vr0=vr0dc8meaabaqaciaacaGaaeqabaqabeGadaaakeaat0uy0HwzTfgDPnwy1egaryqtHrhAL1wy0L2yHvdaiqaacqWFlecsaaa@3762@| + *K*|Σ|^*K*^). Therefore, the space memory is *O *((*m*|ℋ
 MathType@MTEF@5@5@+=feaafiart1ev1aaatCvAUfKttLearuWrP9MDH5MBPbIqV92AaeXatLxBI9gBaebbnrfifHhDYfgasaacH8akY=wiFfYdH8Gipec8Eeeu0xXdbba9frFj0=OqFfea0dXdd9vqai=hGuQ8kuc9pgc9s8qqaq=dirpe0xb9q8qiLsFr0=vr0=vr0dc8meaabaqaciaacaGaaeqabaqabeGadaaakeaat0uy0HwzTfgDPnwy1egaryqtHrhAL1wy0L2yHvdaiqaacqWFlecsaaa@3762@| + *K *|Σ|^*K*^) ∏_*i *_*k*_*i*_) and the running time is *O *(*n*|Σ|(*m*|ℋ
 MathType@MTEF@5@5@+=feaafiart1ev1aaatCvAUfKttLearuWrP9MDH5MBPbIqV92AaeXatLxBI9gBaebbnrfifHhDYfgasaacH8akY=wiFfYdH8Gipec8Eeeu0xXdbba9frFj0=OqFfea0dXdd9vqai=hGuQ8kuc9pgc9s8qqaq=dirpe0xb9q8qiLsFr0=vr0=vr0dc8meaabaqaciaacaGaaeqabaqabeGadaaakeaat0uy0HwzTfgDPnwy1egaryqtHrhAL1wy0L2yHvdaiqaacqWFlecsaaa@3762@| + *K *|Σ|^*K *^)∏_*i *_*k*_*i*_). This *additive *increment compares favorably to simple induction methods [45,53] that introduce a *multiplicative O *(*K*|Σ|^*K*^) factor in time and space complexity for the Markov(*K*) model.

## Results and discussion

We developed an algorithm for precise calculation of the *p*-value for multiple occurrences of multiple motifs with possible overlaps. The running time is linear in the text length and depends on the alphabet size, the maximal motif length, the number of words in the motifs, and the number of occurrences of each motif. The algorithm was implemented in the AHOPRO software. Below we give examples of how *p*-values can be used for studying gene regulation *in silico*, particularly for selecting optimal cutoff values for motifs represented by PWMs. In the subsection *'Comparison with simulation and approximation methods' *we compare our *p*-value computations with the result of Monte Carlo simulations and the Poisson approximation. Our results confirm the accuracy of our algorithm and show in what cases the Poisson approximation [8,11] cannot be employed. In the subsection *'Optimal cutoffs'*, we apply AHOPRO to choose an appropriate cutoff score for Position Weights Matrices. In the subsection *'Assessment of gene regulation'*, we show how AHOPRO can be used for studying regulatory regions containing heterotypic clusters of TFBSs to distinguish genes that are regulated by given transcription factors from those that are not.

As a model example, we use in this section data published in [34] on regulatory clusters in *D. melanogaster*. This compilation includes information on

(i) known binding motifs for transcription factors,

(ii) known CRM regions, and

(iii) known regulatory interactions.

### Comparison with simulation and approximation methods

In our first example we use the *even-skipped stripe 2 *enhancer (*eve2*) [63] of length 728 bp that is known to contain binding sites for TFs *bicoid, kruppel *and *hunchback*. Below we compare *p*-values calculated by the AHOPRO program and those calculated using compound Poisson approximation with *p*-values computed through Monte Carlo simulations.

#### AhoPro and Monte Carlo comparisons

Table 2 displays results of comparison of *p*-values calculated with AHOPRO and with Monte Carlo simulation assuming the Bernoulli model M0. The corresponding results for the first order Markov model M1 are displayed in Table 3. Letters probabilities for M0 and the transition matrix for M1 were evaluated from *eve2 *sequence. We used the PWM cutoff values taken from [34], i.e., 5.3, 5.0, and 6.2 for *bicoid, kruppel*, and *hunchback *respectively. With these threshold values in sequence *eve*2 we have found 3, 4, and 2 occurrences of motifs of each type respectively. In Tables 2 and 3 we listed the *p*-values, i.e, the probabilities to find no less than the observed number of occurrences of motifs in a random text of length *L*, where *L *is the length of *eve2 *enhancer. The number of Monte Carlo simulations was set to 10^6 ^everywhere, except for the triplet (*bcd&kr&hb*), where we did 10^7 ^simulations. The probability to find the observed number of occurrences of (*bcd&kr&hb*) simultaneously in the same simulated sequence is extremely low; thus we increased the number of simulations so that the product of the probability by the number of simulations be greater than 1.

**Table 2:** Comparison of *p*-values calculated by the AHOPRO program, by Monte Carlo simulations and by compound Poisson distribution formula under the M0 model

MOTIF, CUTOFF	OCC.	AHOPRO	MONTE CARLO	POISSON	AHOPRO/MC	AHOPRO/POISSON
*bcd*, 5.3	3	0.012	0.012	0.010	1.00	1.10
*kr*, 5.0	4	0.0044	0.0044	0.0033	1.01	1.34
*hb*, 6.2	2	0.013	0.013	0.012	0.99	1.04
*bcd & kr*	3&4	0.00025	0.00026	3.6E-05	0.99	7.10
*bcd & kr & hb*	3&4&2	6.54E-06	5.8E-06	4.34E-07	1.13	7.13

Comparison of *p*-values calculated for the Markov(0) model by the AHOPRO program with p-values calculated by Monte Carlo simulations and by Poisson formula for motifs of *D. melanogaster *developmental transcription factors *bicoid, kruppel *and *hunchback*.

**Table 3:** Comparison of *p*-values calculated by the AHOPRO program, by Monte Carlo simulation and by compound Poisson distribution formula under the M1 model

MOTIF, CUTOFF	OCC.	AHOPRO	MONTE CARLO	POISSON	AHOPRO/MC	AHOPRO/POISSON
*bcd*, 5.3	3	0.013	0.014	0.012	0.998	1.11
*kr*, 5.0	4	0.011	0.011	0.008	1.01	1.43
*hb*, 6.2	2	0.14	0.14	0.11	0.9987	1.25
*bcd & kr*	3&4	0.00051	0.00051	9.62E-05	0.9991	5.34
*bcd & kr & hb*	3&4&2	6.9E-05	6.97E-05	1.08E-05	0.9889	6.36

Comparison of *p*-values calculated by the AHOPRO program for the Markov(1) model with those calculated by Monte Carlo simulations and by Poisson formula for motifs of *D. melanogaster *developmental transcription factors *bicoid, kruppel*, and *hunchback*.

The results of comparison of the AHOPRO computation with those obtained from simulated random sequences presented in Tables 2 and 3 confirm the accuracy of our algorithm.

#### Poisson approximation

In practical application, compound Poisson distribution [64] is widely used to assess *p*-values of multiple motif occurrences [2,8,34,65]. Here we apply it to compute the probability to observe the given number of motif occurrences when the probabilities of individual words are calculated adopting the M0 or M1 models described above. The results of the comparison given in corresponding columns in Tables 2 and 3 show that the *p*-value calculated using Poisson approximation can be significantly underestimated. This happens most probably because the Poisson approximation does not take into account possible overlaps between motif occurrences and considers motif occurrences as independent. The error increases when the *p*-value is calculated for simultaneous occurrences of several factors, as it is done in the last two rows. In this case, the Poisson approximation *p*-value for a combination of several TFs is calculated as a product of *p*-values calculated independently for each TF. Actually, the motif occurrences can overlap especially when the motifs resemble each other, thus there is no independence, which brings about the error.

### Optimal cutoffs

Below, we use AHOPRO to determine the optimal cutoff values for PWMs of regulatory factors, given the sequences of regulatory region assumedly interacting with the factors. The distribution of occurrences of TF binding sites in corresponding experimentally confirmed regulatory regions is strongly biased [34]. In CRMs binding sites often tend to occur in clusters, which is not the case for random sequences.

Different cutoff values correspond to different numbers of putative binding sites of different quality. The higher the cutoff value, the closer the motif occurrences are to the consensus and the smaller the number of motif occurrences. Therefore, for a given factor it is reasonable to select a cutoff value that minimizes the probability of finding in the random sequence the number of motif occurrences observed in the sequence of the regulatory region.

**Figure 3:**
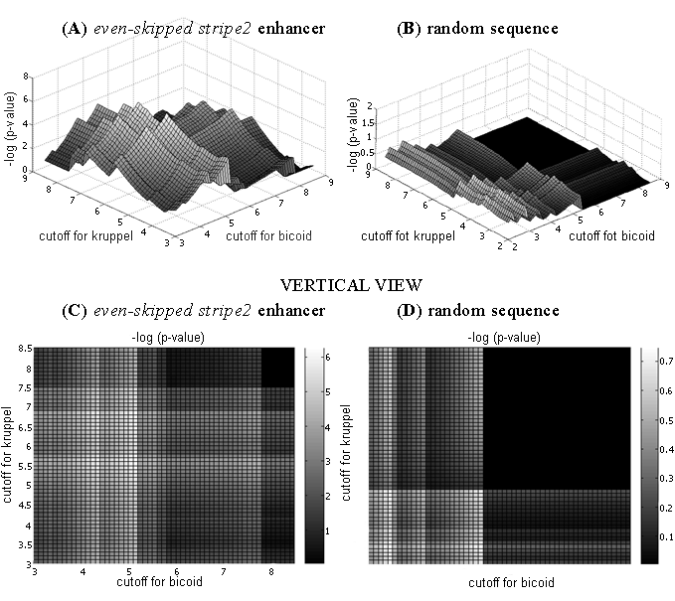
**P-value distribution for *eve2 *and random sequences**. Distribution of log_10 _(*Pvalue*) calculated for the M1 model as a function of cutoff values for PWMs for BICOID and KRUPPEL in the *even-skipped stripe 2 *enhancer (A), in a random sequence (B). View from above: eve2 sequence (C), random sequence (D).

As an example, we considered again transcription factors *bicoid, kruppel*, which are known to regulate the *even-skipped stripe 2 *(*eve2*) enhancer. To select the optimal cutoff value we used the following procedure: first, in the sequence of *eve2 *we counted occurrences of motifs with a score greater than the cutoff with cutoff values varied from 3 to 8.5. Therefore, each pair of cutoff values (*S*_1_, *S*_2_) corresponded to (*k*_1_, *k*_2_) occurrences for motifs of *bicoid *and *kruppel *respectively. For each pair (*k*_1_, *k*_2_), we computed *p*-value *P*_*n *_(*k*_1 _(*S*_1_), *k*_2 _(*S*_2_)), which is denoted below as *P *(*S*_1_, *S*_2_). That is the probability to obtain at least *k*_1 _occurrences of *bicoid*, with scores greater than *S*_1_, and at least *k*_2 _occurrences of *kruppel*, with scores greater than *S*_2_. In Figure 3, a 3D-surface is shown, where (*x, y, z*) corresponds to (*S*_1_, *S*_2_, - log_10 _*P *(*S*_1_, *S*_2_)), the cutoff value for *bicoid *motif, the cutoff value for *kruppel *motif and -logarithm of the corresponding *p*-value calculated for the M1 model respectively. The view to the surface from the above is shown in Figure 3C. The maximal value for – log_10 _*P *(*S*_1_, *S*_2_), 6.3044, is attained when the *bicoid *cutoff is equal to *S*_1 _= 5.1 and the *kruppel *cutoff is equal to *S*_2 _= 5.6. With such cutoff values in the sequence of the *eve2 *enhancer there are *k*_1 _= 6 and *k*_2 _= 4 occurrences of *bicoid *and *kruppel *motifs defined by corresponding PWMs. We believe that the sites that are found with this optimal *p*-value are the best candidates for functional TF binding sites.

For comparison, we simulated random sequences with the same length as the *eve2 *enhancer and the same dinucleotide probabilities. In most of simulated sequences, for the cutoff values for *bicoid *and *kruppel *equal to (*S*_1_, *S*_2_) = (5.1, 5.6) we found no more than one occurrence of each motif. The average number of occurrences is 0.54 for *bicoid *and 0.31 for *kruppel*. The average *p*-value is 0.633. We took one of the random sequences and compared *p*-values calculated for various cutoff values in this random sequence (Figures 3B, 3D) and in the real biological sequence of the *eve2 *enhancer (Figures 3A, 3C). One can see that there are two major differences between *p*-value distributions in really regulated sequences and in the random sequence. First, *p*-values in the random sequence are much greater than those in the enhancer sequence. In particular, maximal – log(*pvalue*) for this random sequence is about 1.02 which is 6.17 times smaller than maximal – log(*pvalue*) for the enhancer sequence (see also Table 4). Second, the shapes of *p*-value distributions are different. For the enhancer sequence, there are only few distinct peaks (4.3, 5.6),(4.3, 6.8), (5.1, 5.6), (5.1, 6.8) whereas for the random sequence we see ridges between (2.2, 2.0) and (2.2, 4.8), and (2.8, 2.0) and (2.8, 4.8). As we expected, it is impossible to choose the appropriate cutoff for PWMs of factors from the random sequence data (Figures 3B and 3D).

**Table 4:** Comparison of *p*-values and cutoff for different sets of DNA sequences

regulatory regions bicoid regulated	minimal pvalue	Cut-off	regulatory regions not regulated by bicoid	minimal pvalue	Cut-off	random seq.	minimal pvalue	Cut-off
Btd crm	3.24E-05	3.4	Gt p. enh.	0.023	2.7	seq. 1	0.16	2.6
Hb P2	4.13E-05	3.7	Hb upstream enh.	0.053	4.4	seq. 2	0.12	1.7
Kni cis element	0.01	5.3	Eve stripe 4+6 enh.	0.41	3.6	seq. 3	0.25	1.2
Kr CD-1 enh.	0.0001	5.1	Eve stripe 3+7 enh.	0.58	2.5	seq. 4	0.065	1.6
Otd early enh.	0.024	5	Ftz upstream enh.	0.037	5.8	seq. 5	0.11	1
Sal blastoder. enh.	8.62E-04	6.5	Ftz	0.28	3.3	seq. 6	0.0087	3.8
Tll PD enh.	0.26	4.2	Ubx PBX enh.	0.196	6.7	seq. 7	0.024	2.9
Tll AD+PD enh.	0.025	8.1	Ubx BXD enh.	0.698	4.6	seq. 8	0.17	3.4
Eve stripe 2 enh.	4.04E-05	5.1	Ubx BX enh. (BRE)	0.05	7.5	seq. 9	0.092	2.8
Eve stripe 1 enh.	8.09E-06	5.2	Ems upstream enh.	0.276	4.4	seq. 10	0.052	3.6
Eve stripe 5 enh.	0.27	3.8	En stripe enh. (intr. 1)	0.049	5	seq. 11	0.13	1.7

Median	8.62E-04	5.1	Median	0.196	4.4	Median	0.1128	2.6

Comparison of minimal *p*-values and best found cutoffs for bicoid PWM calculated (i) in regulatory regions which are regulated by *bicoid*, (ii) in regulatory regions which are not regulated by *bicoid*, and (iii) in random sequences of the same length and with the same dinucleotide distribution as in the *even-skipped stripe 2 *enhancer.

We also would like to address the choice between the M0 and M1 models. We observed, that in almost all cases the *p*-value calculated for the M0 model is smaller than the *p*-value calculated for the M1 model. This can probably be explained by the fact that using the M1 model we take into account more information about the real sequence than in the M0 model. Nevertheless, the difference is not crucial; for instance, the greatest value of the ratio between *p*-values calculated adopting the M0 and M1 for *bicoid *and *kruppel *is about 3.62 for the *eve2 *enhancer. So, the M0 model can be equally used in practical applications.

### Assessment of gene regulation

Enhancers may contain clusters of TF binding sites for gene regulators. In such cases, *p*-value computation can be used to distinguish genes that are regulated by a given transcription factor from those that are not. To illustrate this, we took PWM for TF *bicoid *and calculated *p*-values for different cutoff values in various sets of sequences:

- regulatory regions which are regulated by *bicoid*, the positive set;

- regulatory regions which are not regulated by *bicoid*, the negative set;

- random sequences of the same length as *eve2 *enhancer and with the same dinucleotide distribution, the random set.

Minimal *p*-value and the corresponding cutoff value for 11 sequences in each set are presented in Table 4. Comparing the *p*-values we observed that *p*-values calculated for the positive set generally were significantly smaller than those, calculated for the negative and for the random sets.

The median for the *p*-value in the positive set is equal to 8.62E-04. But there are some exceptions, for instance, the *tailless PD *enhancer with a minimal *p*-value that is equal to 0.26 and the *even-skipped stripe 5 *enhancer with the minimal *p*-value that is equal to 0.27. Despite the fact that these genes are reported to be regulated by *bicoid *and that there are experimentally confirmed individual *bicoid *binding sites in these sequences, these sequences do not contain clusters of *bicoid *binding sites.

Most *p*-values calculated for the negative set, (second set in Table 4), are significantly higher than *p*-values calculated for the positive set. But we observed rather small *p*-values for sequences of the *giant posterior *enhancer (0.023), the *hunchback *upstream enhancer (0.053), the *fushi tarazu *upstream enhancer (0.037), the *ultrabithorax BX *enhancer (0.05), and the *engrailed *stripe enhancer (0.049). We believe that this can be explained by the fact that these regions contain clusters of binding sites of regulatory factors with motifs that are similar to the *bicoid *motif. Indeed, it was experimentally shown that TF *kruppel *regulates the *giant *posterior enhancer, TF *tailless *regulates the *hunchback *upstream enhancer and the *ultrabithorax BX *enhancer, and TF *fushi tarazu *regulates the *fushi tarazu *upstream enhancer, the *ultrabithorax BX *enhancer and the *engrailed *stripe enhancer. All these motifs of *kruppel, tailless *and *fushi tarazu *exhibit some similarity to the *bicoid *motif. This observation shows the necessity to use some sort of conditional *p*-values in order to distinguish between the true *bicoid *clusters and the clusters of weak *bicoid *sites induced by presence of the clusters of other TF sites [67]. Moreover, the apparent false positive hit (*p*-value = 0.05, cutoff = 7.5) in a region that was not reported to be regulated by *bicoid *seems to be related to the real *bicoid *binding, although not necessarily functional.

For the random set, i.e., sequences simulated with the same dinucleotide probabilities as in the *even-skipped stripe 2 *enhancer, we observe a rather broad range of minimal *p*-values, from 0.0087 for the 6th sample to 0.25 for the 3rd sample. It shows that the predictive power of this approach is limited to the case of regulatory sequences containing clusters of motifs.

## Conclusion

In this work we have developed an algorithm inspired by the Aho-Corasick pattern matching algorithm that allows precise calculation of the probability to find given motif conformation in a random text. It was implemented in the AHOPRO software for the Bernoulli model and the Markov model of order 1 of random sequences. There would be no difficulty in extending our approach for Markov models of order *k*, *k *> 1. We compared probabilities computed with AHOPRO with those computed by compound Poisson distribution and showed that in the case of multiple occurrences of multiple motifs the Poisson approximation often substantially underestimate the *p*-value.

As we have demonstrated, the statistical significance of multiple motif occurrence in the text can be efficiently calculated with a simple algorithm. This can give an independent criteria to improve the results of site extraction algorithms, which still performs rather poorly. P-values or E-values are used in such programs as BLAST and make quantities to which practicing biologists are used to. Thus, adopting this measure to motif extraction (for a single or multiple motif occurrences) would greatly help the users who use motif extraction analysis as a preliminary stage for experiments in the lab. On the other hand, our algorithm is not connected with a particular motif extraction program, and uses a most general motif representation, the list of the allowed words [35], as input. Thus, it can be used when the results of several motif extraction algorithms are compared, for instance in the interpretation of ChIP-chip experiments [5]. In addition, our algorithm AHOPRO can easily be extended to amino acid sequences and applied in identification of protein domain signatures.

## Authors' contributions

VM initiated the study by pointing at the biological problem. JC suggested the initial idea of using Aho-Corasick structure. The final version of the algorithm was developed in discussions between JC, VB, MR and MAR. JC and VB developed the implementation. VB obtained results on simulated and biological sequences. VB designed the web site. MR, MAR, VB and VM participated in manuscript writing. MR and VM coordinated the study. All authors read and approved the final manuscript.

## Supplementary Material

Additional file 1 Bernoulli text model. Probability to find multiple occurrences of a single motif. The detailed description of the algorithm for the *p*-value calculation in the case of multiple occurrences of a single motif. Click here for file

Additional file 2 Tree construction from PWM motif representation. The brief description of the procedure of the prefix tree construction from PWM motif representation. Click here for file

Additional file 3 Tree construction from PWM motif representation. Steps of the prefix tree construction for a PWM and a given cut-off. Click here for file
