# Supplementary material for: Exact p-value calculation for heterotypic clusters of regulatory motifs and its application in computational annotation of cis-regulatory modules
Source: Algorithms Mol Biol. 2007 Oct 10;2:13. doi: 10.1186/1748-7188-2-13 (PMC2174486; doi:10.1186/1748-7188-2-13)
Supplement: Additional file 1 — Bernoulli text model. Probability to find multiple occurrences of a single motif. The detailed description of the algorithm for the p-value calculation in the case of multiple occurrences of a single motif. [file 1748-7188-2-13-S1.pdf]

Additional file 1: Exact p-value calculation for heterotypic clusters of regulatory motifs and its use in computational annotation of cis-regulatory modules

Valentina Boeva*[1,2], Julien Clément[3] , Mireille Régnier[2] , Mikhail A. Roytberg[4,5] and Vsevolod J. Makeev[1,6]

[1]Institute of Genetics and Selection of Industrial Microorganisms, GosNIIGenetika, 117545 Moscow, Russia
[2]INRIA Rocquencourt, 78153 Le Chesnay, France
[3]GREYC, CNRS UMR 6072, Laboratoire d'informatique, 14032 Caen, France
[4]Institute of Mathematical Problems of Biology, Russian Academy of Sciences, Puschino, Moscow Region, Russia
[5]Puschino State University, Puschino, Moscow Region, Russia
[6]Engelhardt Institute of Molecular Biology, Russian Academy of Sciences, Moscow, Russia

Email: Valentina Boeva*- valeyo@imb.ac.ru; Julien Clément - Julien.Clement@info.unicaen.fr; Mireille Régnier - Mireille.Regnier@inria.fr; Mikhail A. Roytberg - mroytberg@impb.psn.ru; Vsevolod J. Makeev - makeev@genetika.ru;

*Corresponding author

Bernoulli text model. Probability to find multiple occurrences of a single motif

Here the problem is to calculate the p-value of finding of at least k possibly overlapping occurrences of a motif $\mathcal{H}$ in the random text T_n generated as a Bernoulli random model over Σ.

Again, all texts T_i are divided into classes containing different number of occurrences of $\mathcal{H}$.

Definition 1 *A text T_i belongs to the class $C_i(l, q)$, $0 \le l \le k-1$ iff*

1. *Length of T_i equals i,*
2. *T_i contains exactly l possibly overlapping words from $\mathcal{H}$;*
3. *A traversal $AC\,(\mathcal{T}(\mathcal{H}), T_i)$ ends in node q.*

A text T_i belongs to the class $G_i(k)$ if and only if the length of T_i equals i and T_i contains at least k possibly overlapping occurrences of words from $\mathcal{H}$.

The idea of the algorithm in this case is based on two following observations. First, let a traversal $AC\,(\mathcal{T}(\mathcal{H}), T_i)$ end at node q; $T_i \in C_i(l, q)$, where $0 \le l \le k-1$ and $\delta(q, a) \notin \mathcal{H}$. Then the number of occurrences does not change after the transition from T_i to $T_i a$. Second, if text T_i, of length j has a prefix

from $G_i(k)$, $j > i$, then T_i belongs to $G_j(k)$. Similarly to the previous sections, one can consider probabilities $\mathbf{P}(C_i(l,q))$ and $\mathbf{P}(G_i(k))$; the desired p-value $\mathbf{P}(L_n(k,\mathcal{H}))$ is equal to $\mathbf{P}(G_n(k))$.

The initial values are: $\mathbf{P}(C_0(0,\epsilon)) = 1$; $\mathbf{P}(C_0(l,q)) = 0$ for any $q \neq \epsilon$, and any l: $0 \le l \le k-1$; $\mathbf{P}(G_0(k)) = 0$.

Expression for languages (5, from the main text) becomes transformed to:

$$G_{i+1}(k) = \{\bigcup_{a\in\Sigma} G_i(k)\cdot a\} \cup \{\bigcup_{(q,a):\delta(q,a)\in\mathcal{H}} C_i(k-1,q)\cdot a\} \ . \tag{1}$$

It is complemented by a series of equations for different l, $1 \le l \le k-1$:

$$C_{i+1}(l,q') = \begin{cases} \cup_{(q,a):\delta(q,a)=q'} C_i(l,q)\cdot a & \text{if } q' \notin \mathcal{H} \\ \cup_{(q,a):\delta(q,a)=q'} C_i(l-1,q)\cdot a & \text{if } q' \in \mathcal{H} \end{cases} \tag{2}$$

for $l = 0$ we have:

$$\forall q' \in Q_{\mathcal{H}} \setminus \mathcal{H} : C_{i+1}(0,q') = \bigcup_{(q,a):\delta(q,a)=q'} C_i(0,q)\cdot a \ . \tag{3}$$

Remark that in contrast to (6, from the main text), q and q' may here belong to $\mathcal{H}$.

The series of equations for probabilities below steadily follow from Equations (1), (2) and (3).

$$\mathbf{P}(G_{i+1}(k)) = \mathbf{P}(G_i(k)) + \sum_{(q,a):\delta(q,a)\in\mathcal{H}} \mathbf{P}(C_i(k-1,q))\cdot p(a), \tag{4}$$

$$\mathbf{P}(C_{i+1}(l,q')) = \begin{cases} \sum_{(q,a):\delta(q,a)=q'} \mathbf{P}(C_i(l,q))\cdot p(a) & \text{if } q' \notin \mathcal{H} \\ \sum_{(q,a):\delta(q,a)=q'} \mathbf{P}(C_i(l-1,q))\cdot p(a) & \text{if } q' \in \mathcal{H} \end{cases} \tag{5}$$

$$\forall q' \notin \mathcal{H} : \mathbf{P}(C_{i+1}(0,q')) = \sum_{(q,a):\delta(q,a)=q'} \mathbf{P}(C_i(0,q))\cdot p(a) \ . \tag{6}$$

The running time of the computation of $G_i(k)$ and $C_{i+1}(l,q)$ for any l, $0 \le l \le k-1$, is $O(|Q_{\mathcal{H}}|\cdot|\Sigma|)$; therefore the total time of all n stages of p-value computation is $O(|Q_{\mathcal{H}}|\cdot|\Sigma|\cdot nk)$.
