# Supplementary material for: Exact p-value calculation for heterotypic clusters of regulatory motifs and its application in computational annotation of cis-regulatory modules
Source: Algorithms Mol Biol. 2007 Oct 10;2:13. doi: 10.1186/1748-7188-2-13 (PMC2174486; doi:10.1186/1748-7188-2-13)
Supplement: Additional file 2 — Tree construction from PWM motif representation. The brief description of the procedure of the prefix tree construction from PWM motif representation. [file 1748-7188-2-13-S2.pdf]

# Additional file 2: Exact p-value calculation for heterotypic clusters of regulatory motifs and its use in computational annotation of *cis*-regulatory modules

Valentina Boeva*[1,2], Julien Clément[3] , Mireille Régnier[2] , Mikhail A. Roytberg[4,5] and Vsevolod J. Makeev[1,6]

[1]Institute of Genetics and Selection of Industrial Microorganisms, GosNIIGenetika, 117545 Moscow, Russia
[2]INRIA Rocquencourt, 78153 Le Chesnay, France
[3]GREYC, CNRS UMR 6072, Laboratoire d'informatique, 14032 Caen, France
[4]Institute of Mathematical Problems of Biology, Russian Academy of Sciences, Puschino, Moscow Region, Russia
[5]Puschino State University, Puschino, Moscow Region, Russia
[6]Engelhardt Institute of Molecular Biology, Russian Academy of Sciences, Moscow, Russia

Email: Valentina Boeva*- valeyo@imb.ac.ru; Julien Clément - Julien.Clement@info.unicaen.fr; Mireille Régnier - Mireille.Regnier@inria.fr; Mikhail A. Roytberg - mroytberg@impb.psn.ru; Vsevolod J. Makeev - makeev@genetika.ru;

*Corresponding author

### Tree construction from PWM motif representation

Until now we defined any motif as a list of allowed words. In practical application, a motif is often defined by a Position Weight Matrix (PWM). PWM is a $|\Sigma| \times L$-matrix, where $|\Sigma|$ is the alphabet size and $L$ is the motif length. In this case, each word has a score calculated as a sum of matrix elements corresponding to letters at different positions in the word (recall subsection *Representation of protein binding motifs in nucleotide sequences*). Motif includes all words with PWM scores higher than some given threshold. This list of high scoring words can be very large. Here it becomes important that our algorithm actually employs a tree, rather than a list of words *per se*. Actually it is possible to by-pass testing of all words one after another if they score above the threshold. For high thresholds only a small fraction of all words remains, and the tree for this set can be efficiently constructed directly from the PWM and the threshold [1].

First, let us transform the initial PWM $M = \|m_{i,j}\|_{|\Sigma|}^{L}$. One defines the transformed matrix $\tilde{M} = \|\tilde{m}_{i,j}\|_{|\Sigma|}^{L}$ as

$$\tilde{m}_{i,j} = \max_{\beta} m_{\beta,j} - m_{i,j}. \tag{1}$$

Then, for a sequence $W$ of length $L$ the transformed score $\tilde{S}$ writes as

$$\tilde{S} = \sum_{j=1}^{L} \max_{\beta} m_{\beta,j} - S,$$

where $S$ is the score under initial matrix $M$.

The set of words scoring higher than a threshold $T$ with the PWM $M$ is the set of words scoring *lower* than the threshold

$$\tilde{T} = \sum_{j=1}^{L} \max_{\beta} m_{\beta,j} - T, \qquad (2)$$

with the transformed matrix $\tilde{M}$. Thus, scores becomes substituted for nonnegative penalties. Since the score for the word is calculated as a sum over all word positions, if the prefix of a word gets a total penalty greater than the threshold, the entire word would never obtain the total penalty greater than the threshold independently from the remaining word suffix, and thus would not contribute to the sought set.

The tree construction is started from the root, and corresponds to the extension of the motif from left to right. All possible letters are subject to concatenation. However, one adds an edge corresponding to a letter only if the resulting prefix scores lower than $\tilde{T}$ with $\tilde{M}$ [see Figure in Additional File 3].

## References


1. Boeva V, Clément J, Régnier M, Vandenbogaert M: **Assessing the significance of Sets of Words**. In *CPM'05*, *Volume 3537 of* Lecture Notes in Computer Science, Springer-Verlag 2005:358–370. [Proc. CPM'05, Jeju Island, Korea].


### Additional Figure 1 - Tree construction from PWM motif representation.

Given a Position Weight $4 \times 3$ Matrix $M$ and a cutoff value $T$ we recalculate $\tilde{M}$ and $\tilde{T}$ using formulae (1) and (2). Then at each step $(i)_{1 \le i \le 3}$ we keep nodes corresponding to words that score lower than $\tilde{T}$ with matrix $\tilde{M}$.
