# Supplementary figures and images for: Exact p-value calculation for heterotypic clusters of regulatory motifs and its application in computational annotation of cis-regulatory modules
Source: Algorithms Mol Biol. 2007 Oct 10;2:13. doi: 10.1186/1748-7188-2-13 (PMC2174486; doi:10.1186/1748-7188-2-13)

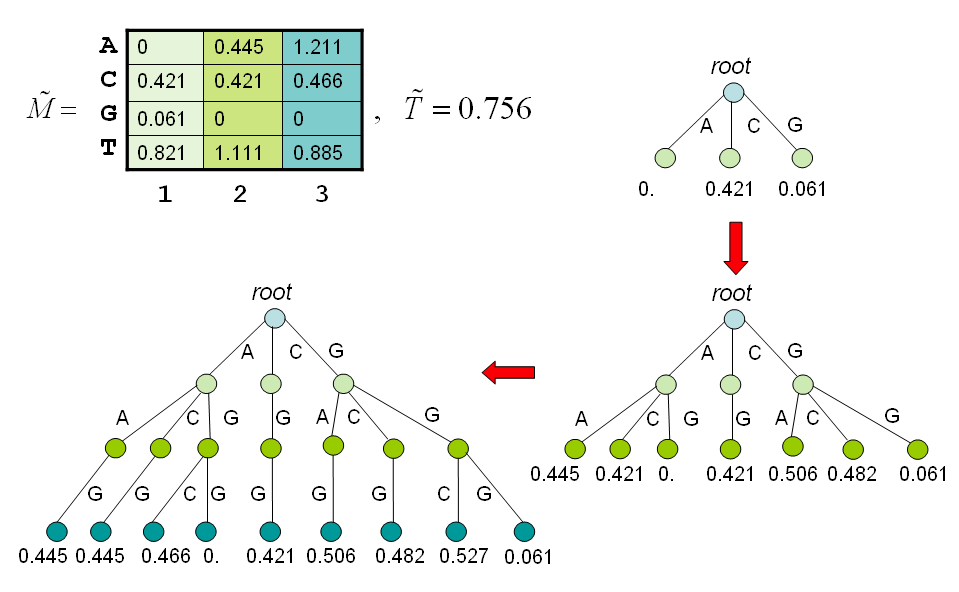

Supplement: Additional file 3 — Tree construction from PWM motif representation. Steps of the prefix tree construction for a PWM and a given cut-off. [file 1748-7188-2-13-S3.bmp]
